# The Genus *Alternanthera*: Phytochemical and Ethnopharmacological Perspectives

**DOI:** 10.3389/fphar.2022.769111

**Published:** 2022-04-11

**Authors:** Rajeev K. Singla, Vivek Dhir, Reecha Madaan, Deepak Kumar, Simranjit Singh Bola, Monika Bansal, Suresh Kumar, Ankit Kumar Dubey, Shailja Singla, Bairong Shen

**Affiliations:** ^1^ Institutes for Systems Genetics, Frontiers Science Center for Disease-related Molecular Network, West China Hospital, Sichuan University, Chengdu, China; ^2^ iGlobal Research and Publishing Foundation, New Delhi, India; ^3^ Chitkara College of Pharmacy, Chitkara University Punjab, Rajpura, India; ^4^ Department of Health and Family Welfare, Civil Hospital, Rampura Phul, India; ^5^ Akal College of Pharmacy and Technical Education, Mastuana Sahib, Sangrur, India; ^6^ Department of Pharmaceutical Sciences and Drug Research, Punjabi University, Patiala, India; ^7^ Institute of Scholars, Bengaluru, India

**Keywords:** alternanthera, anticancer, antidiabetic, antimicrobial, flavonoids, triterpenoid saponins, natural products (NP)

## Abstract

**Ethnopharmacological relevance:** The genus *Alternanthera* (*Amaranthaceae*) comprises 139 species including 14 species used traditionally for the treatment of various ailments such as hypertension, pain, inflammation, diabetes, cancer, microbial and mental disorders.

**Aim of the review:** To search research gaps through critical assessment of pharmacological activities not performed to validate traditional claims of various species of *Alternanthera.* This review will aid natural product researchers in identifying *Alternanthera* species with therapeutic potential for future investigation.

**Materials and methods:** Scattered raw data on ethnopharmacological, morphological, phytochemical, pharmacological, toxicological, and clinical studies of various species of the genus *Alternanthera* have been compiled utilizing search engines like SciFinder, Google Scholar, PubMed, Science Direct, and Open J-Gate for 100 years up to April 2021.

**Results:** Few species of *Alternanthera* genus have been exhaustively investigated phytochemically, and about 129 chemical constituents related to different classes such as flavonoids, steroids, saponins, alkaloids, triterpenoids, glycosides, and phenolic compounds have been isolated from 9 species. Anticancer, antioxidant, antibacterial, CNS depressive, antidiabetic, analgesic, anti-inflammatory, and immunomodulator effects have been explored in the twelve species of the genus. A toxicity study has been conducted on 3 species and a clinical study on 2 species.

**Conclusions:** The available literature on pharmacological studies of *Alternanthera* species reveals that few species have been selected based on ethnobotanical surveys for scientific validation of their traditional claims. But most of these studies have been conducted on uncharacterized and non-standardized crude extracts. A roadmap of research needs to be developed for the isolation of new bioactive compounds from *Alternanthera* species, which can emerge out as clinically potential medicines.

## Introduction

**FIGURE 1:**
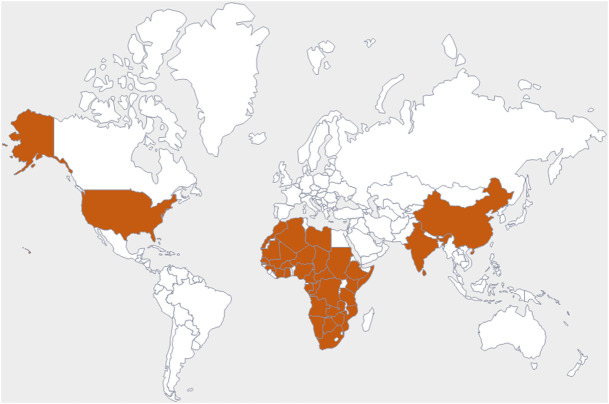
Commonly observed geographical distribution of *Alternanthera* species, indicated in dark orange.

The family *Amaranthaceae* comprises 65 genera and about 850 species (Hundiwale et al., 2012; Chandrashekhar, 2019). These species are mainly distributed in tropical regions of the United States of America, Africa, and India. Amongst 65 genera and 850 species, only 17 genera and 50 species have been recorded to be found in India. The plants from this family include herbs, shrubs, and universal weeds. The genus *Alternanthera*, a significant delegate of the family *Amaranthaceae* was coined by by Forsskal in 1775. The genus *Alternanthera* comprises roughly 139 species which are distributed in India, China, Sri Lanka, the United States of America, and Africa (Figure 1). Though not complete and exhaustive, but phytochemical characterization was found to be reported that of *Alternanthera sessilis* (L.) R.Br. ex DC., *Alternanthera philoxeroides* (Mart.) Griseb., *Alternanthera brasiliana* (L.) Kuntze, *Alternanthera hirtula* (Mart.) R.E.Fr., *Alternanthera praelonga* A.St.-Hil., *Alternanthera littoralis* P.Beauv., *Alternanthera bettzickiana* (Regel) G.Nicholson, and *Alternanthera pungens* Kunth (Table 1 with complete details).

**TABLE 1:** Chemical constituents isolated from genus *Alternanthera*.

S.No	Name	Structure	Source	Plant part	References
	Benzopyran				
1	3,3′-(Propane-2,2diyl)-bis-3,4,5,6,7,8-hexahydro-1H-isochromene	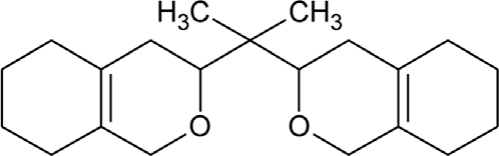	*Alternanthera sessilis* (L.) R.Br. ex DC.	Leaves	Sundar et al. (2019)
	Flavonoids				
	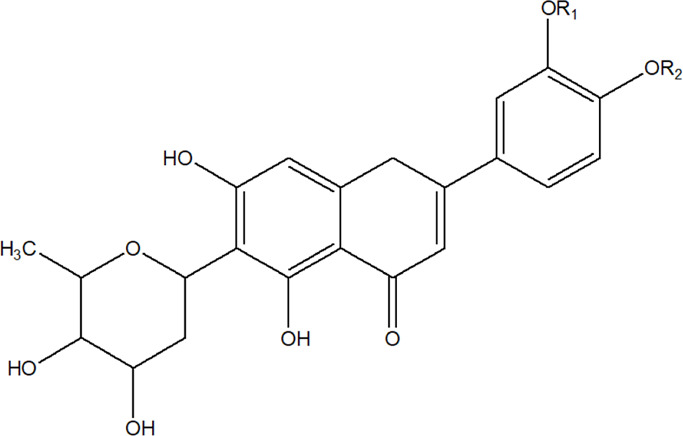				
2	Luteolin-6-C-*β*-D-boivinopyranosyl-3′-*O*-*β*-D-glucopyranoside	R_1_ = Glu; R_2_ = H	*Alternanthera philoxeroides* (Mart.) Griseb	Not specified	Li et al. (2016)
3	Chrysoeriol-6-C-*β*-D-boivinopyranosyl-4′-*O*-*β*-D-glucopyranoside	R_1_ = CH_3_; R_2_ = Glu	*Alternanthera philoxeroides* (Mart.) Griseb	Not specified	Li et al. (2016)
4	Luteolin-6-C-*β*-D-boivinopyranosyl-4′-*O*-*β*-D-glucopyranoside	R_1_ = H; R_2_ = Glu	*Alternanthera philoxeroides* (Mart.) Griseb	Not specified	Li et al. (2016)
5	Luteolin-6-C-*β*-D-boivinopyranoside or Alternanthin B or Demethyl-torosaflavone B	R_1_ = H; R_2_ = H	*Alternanthera philoxeroides* (Mart.) Griseb	Aerial parts	Khamphukdee et al. (2018)
6	Chrysoeriol-6-C-*β*-D-boivinopyranoside or Alternanthin A	R_1_ = CH_3_; R_2_ = H	*Alternanthera philoxeroides* (Mart.) Griseb	Aerial parts	Zhou et al. (1988)
					Fan, (2008)
					Li et al. (2016)
					Khamphukdee et al. (2018)
7	Chrysoeriol 6-C-β-boivinopyranosyl-7-O-β-glucopyranoside	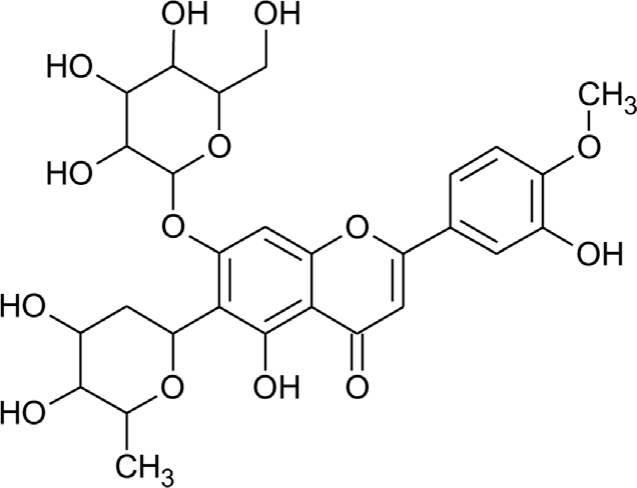	*Alternanthera philoxeroides* (Mart.) Griseb	Aerial parts	Fan, (2008)
	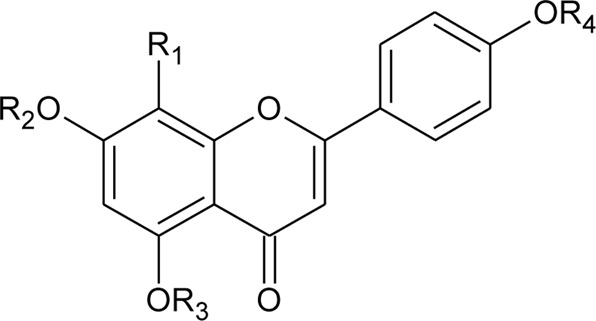				
8	2′′-*O*-Ramnosylvitexin	R_1_ = Glucoslyl (1→6) ramnoside; R_2_ = R_3_ = R_4_ = H	*Alternanthera brasiliana* (L.) Kuntze	Aerial parts	Araujo et al. (2014)
9	4′,5,7-trimethoxy-2′′-O-ramnosylvitexin	R_1_ = Glucoslyl (1→6) ramnoside; R_2_ = R_3_ = R_4_ = CH_3_	*Alternanthera brasiliana* (L.) Kuntze	Aerial parts	Araujo et al. (2014)
10	Ligustroflavone	R_1_ = H; R_2_ = Glucoslyl (2→1) ramnoside, (6→1) ramnoside; R_3_ = R_4_ = H	*Alternanthera brasiliana* (L.) Kuntze	Aerial parts	Araujo et al. (2014)
11	Vitexin or Apigenin-8-C-glucoside	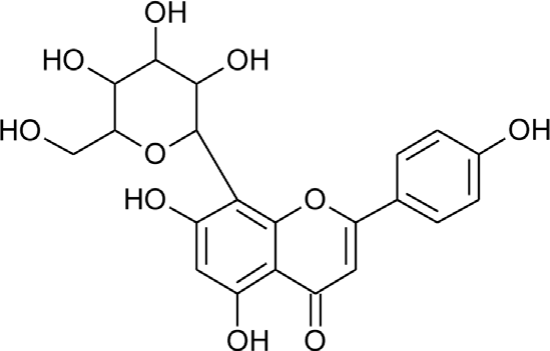	*Alternanthera brasiliana* (L.) Kuntze, *Alternanthera sessilis* (L.) R.Br. ex DC., *Alternanthera philoxeroides* (Mart.) Griseb., *Alternanthera hirtula* (Mart.) R.E.Fr., *Alternanthera praelonga* A.St.-Hil., *Alternanthera littoralis* P.Beauv	Aerial parts; Leaves	Salvador and Dias, (2004)
					Correa et al. (2016)
					Deladino et al. (2017)
	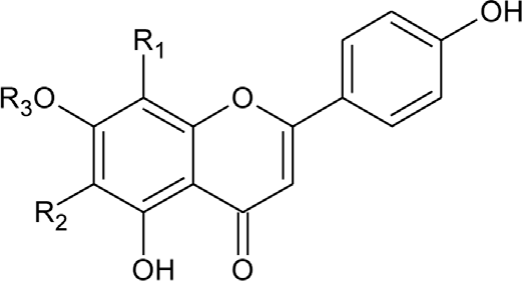 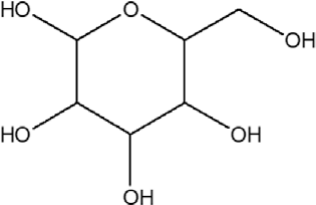				
	(*β*-D-glucopyranosyl)				
12	7-*O*-*β*-D-glucopyranosyl-6-*C*-*β*- D-glucopyranosyl-apigenin	R_1_ = H; R_2_ = R_3_ = *β*-D-glucopyranosyl	*Alternanthera bettzickiana* (Regel) G.Nicholson	Flower	Petrus et al. (2014b)
13	6-*C*-*β*- D-glucopyranosyl-apigenin	R_1_ = R_3_ = H; R_2_ = *β*-D-glucopyranosyl	*Alternanthera bettzickiana* (Regel) G.Nicholson	Flower	Petrus et al. (2014b)
14	8-*C*-*β*- D-glucopyranosyl-apigenin	R_1_ = *β*-D-glucopyranosyl; R_2_ = R_3_ = H	*Alternanthera bettzickiana* (Regel) G.Nicholson	Flower	Petrus et al. (2014b)
15	5,7,4′-trihydroxyflavone	R_1_ = R_2_ = R_3_ = H	*Alternanthera bettzickiana* (Regel) G.Nicholson	Flowers	Petrus et al. (2014a)
16	Isovitexin	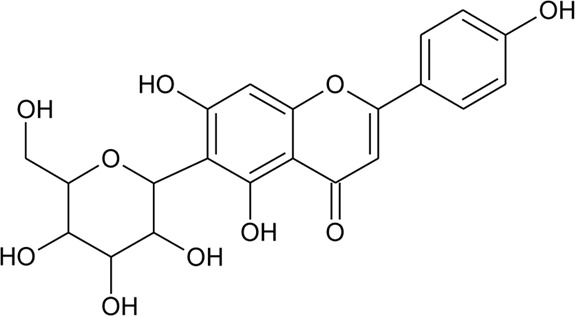	*Alternanthera littoralis* P.Beauv	Aerial parts	Salvador and Dias, (2004)
	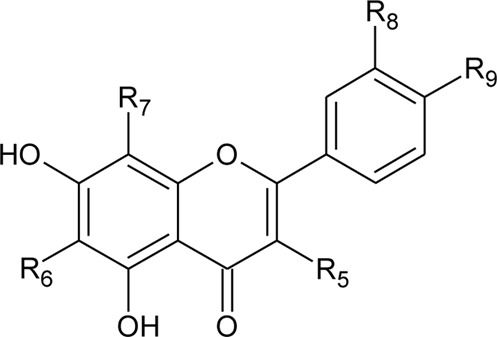				
17	Kaempferol	R_5_ = R_9_ = OH; R_6_ = R_7_ = R_8_ = H	*Alternanthera brasiliana* (L.) Kuntze, *Alternanthera littoralis* P.Beauv., *Alternanthera sessilis* (L.) R.Br. ex DC.	Aerial parts; Leaves; Whole Plant	Salvador and Dias, (2004)
					Salvador et al. (2006)
					Salvador et al. (2009)
					Deladino et al. (2017)
18	Quercetin-3-methyl ether	R_5_ = OCH_3_; R_6_ = R_7_ = H; R_8_ = R_9_ = OH	*Alternanthera littoralis* P.Beauv.; *Alternanthera sessilis* (L.) R.Br. ex DC.	Aerial parts	Salvador and Dias, (2004)
					Souza et al. (2007)
					Salvador et al. (2009)
19	Quercetin	R_5_ = R_8_ = R_9_ = OH; R_6_ = R_7_ = H	*Alternanthera brasiliana* (L.) Kuntze, *Alternanthera littoralis* P.Beauv.; *Alternanthera sessilis* (L.) R.Br. ex DC.; *Alternanthera hirtula* (Mart.) R.E.Fr.; *Alternanthera philoxeroides* (Mart.) Griseb	Aerial parts; Whole plant	Salvador and Dias, (2004)
					Salvador et al. (2006)
					Souza et al. (2007)
					Fan, (2008)
					Salvador et al. (2009)
					Correa et al. (2016)
					Deladino et al. (2017)
					Vani et al. (2018)
					Zhang et al. (2018)
20	Luteolin	R_5_ = R_6_ = R_7_ = H; R_8_ = R_9_ = OH	*Alternanthera philoxeroides* (Mart.) Griseb	Aerial parts	Fan, (2008)
21	2″-*O*-*α*-L-rhamnopyranosyl vitexin	R_5_ = R_6_ = R_8_ = H; R_7_ = C-Glu′′′→2′′ Rha (d); R_9_ = OH	*Alternanthera brasiliana* (L.) Kuntze, *Alternanthera littoralis* P.Beauv.; *Alternanthera sessilis* (L.) R.Br. ex DC.	Aerial parts; whole plant	Salvador and Dias, (2004)
					Salvador et al. (2006)
					Souza et al. (2007)
					Salvador et al. (2009)
					Deladino et al. (2017)
22	2″-*O*-*β*-D-glucopyranosyl vitexin	R_5_ = R_6_ = R_8_ = H; R_7_ = C-Glu′′′→2′′ Glu (d); R_9_ = OH	*Alternanthera brasiliana* (L.) Kuntze, *Alternanthera littoralis* P.Beauv.; *Alternanthera sessilis* (L.) R.Br. ex DC.	Aerial parts; whole plant	Salvador and Dias, (2004)
					Salvador et al. (2006)
					Souza et al. (2007)
					Salvador et al. (2009)
					Deladino et al. (2017)
23	Acacetin 8-c-[*α*-L-rhamnopyranoyl-(1→2)-*β*-D-glucopyranoside]	R_5_ = R_6_ = R_8_ = H; R_7_ = C-Glu′′′→2′′ Rha (d); R_9_ = OCH_3_	*Alternanthera littoralis* P.Beauv.*, Alternanthera sessilis* (L.) R.Br. ex DC.	Aerial parts; whole plant	Salvador et al. (2006)
					Souza et al. (2007)
					Salvador et al. (2009)
24	Quercetin 3-*O*-*α*-L-rhamnosyl-(1→6)-*β*-D-glucopyranoside	R_5_ = d; R_6_ = H; R_7_ = H; R_8_ = OH; R_9_ = OH	*Alternanthera littoralis* P.Beauv	Aerial parts	Souza et al. (2007)
25	Isorhamnetin 3-*O*-*α*-L-rhamnosyl-(1→6)-*β*-D-glucopyranoside	R_5_ = d; R_6_ = H; R_7_ = H; R_8_ = OH; R_9_ = OCH_3_	*Alternanthera littoralis* P.Beauv	Aerial parts	Souza et al. (2007)
	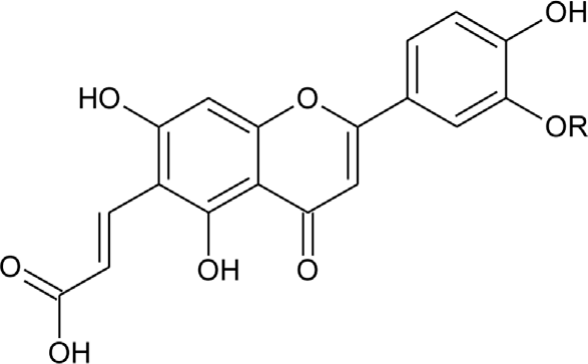				
26	Torosaflavone E	R = CH_3_	*Alternanthera philoxeroides* (Mart.) Griseb	Aerial parts	Khamphukdee et al. (2018)
27	Demethyl torosaflavone D	R = H	*Alternanthera philoxeroides* (Mart.) Griseb	Aerial parts	Khamphukdee et al. (2018)
28	Luteolin-8-C-E-propenoic acid	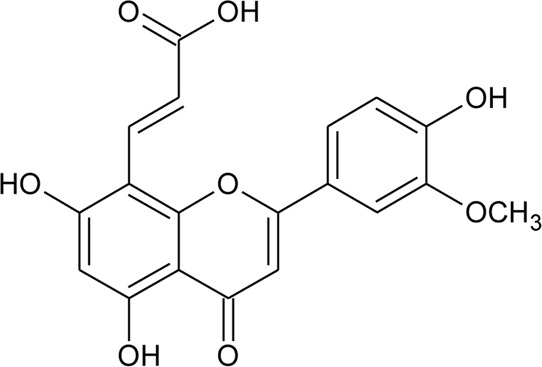	*Alternanthera philoxeroides* (Mart.) Griseb	Aerial parts	Khamphukdee et al. (2018)
29	Chrysoeriol-7-*O*-rhamnoside	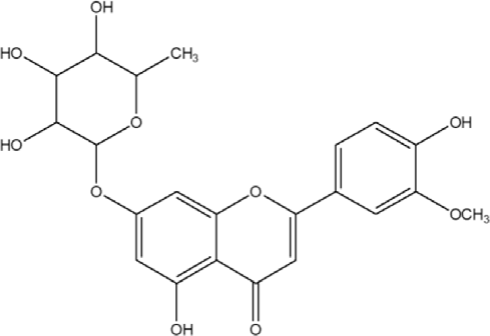	*Alternanthera philoxeroides* (Mart.) Griseb	Aerial parts	Khamphukdee et al. (2018)
	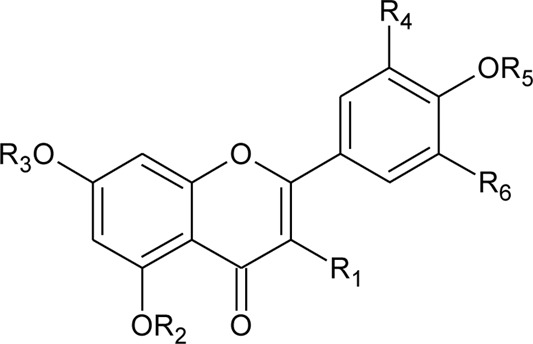				
30	Crysoeriol (5,7,4′-trihydroxy-3′-methoxyflavone)	R_1_ = R_2_ = R_3_ = R_4_ = R_5_ = H; R_6_ = OCH_3_	*Alternanthera brasiliana* (L.) Kuntze	Flowers	Facundo et al. (2012)
31	Tricin (5,7,4 -trihydroxy-3′,5′ -dimethoxyflavone)	R_1_ = R_2_ = R_3_ = R_5_ = H; R_4_ = R_6_ = OCH_3_	*Alternanthera brasiliana* (L.) Kuntze	Flowers	Facundo et al. (2012)
32	7-O-β-D-glucopyranoside-5,4′-dihydroxy-3′-methoxyflavone	R_1_ = R_2_ = R_4_ = R_5_ = H; R_6_ = OCH_3_; R_3_ = O-*β*-D-glucopyranoside	*Alternanthera brasiliana* (L.) Kuntze	Flowers	Facundo et al. (2012)
	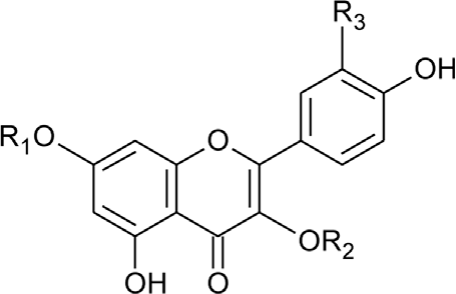 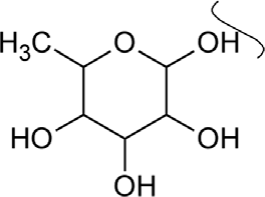			
	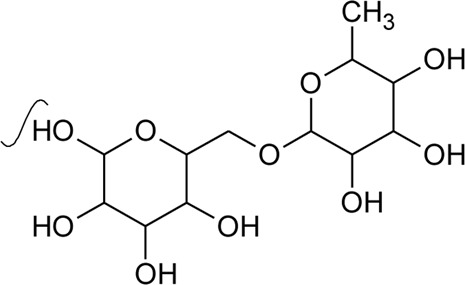 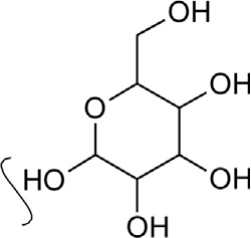			
33	Kaempferol-3-*O*-robinobioside-7-*O*-*α*-L-rhamnopyranoside or Robinin or Kaempferol-3-*O*-rutinoside-7-*O*-*α*-L-rhamnopyranoside	R_1_ = a; R_2_ = b; R_3_ = H	*Alternanthera brasiliana* (L.) Kuntze, *Alternanthera sessilis* (L.) R.Br. ex DC.	Leaves	Brochado et al. (2003)
					Deladino et al. (2017)
34	Kaempferol-7- O-glucoside	R_1_ = c; R_2_ = H; R_3_ = H	*Alternanthera brasiliana* (L.) Kuntze, *Alternanthera sessilis* (L.) R.Br. ex DC.	Leaves	Deladino et al. (2017)
35	Quercetin 3-*β*-D-glucoside	R_1_ = H; R_2_ = c; R_3_ = H	*Alternanthera brasiliana* (L.) Kuntze, *Alternanthera sessilis* (L.) R.Br. ex DC.	Leaves	Deladino et al. (2017)
36	Quercetin-3-*O*-robinobioside-7-*O*-*α*-L-rhamnopyranoside or Clovin	R_1_ = a; R_2_ = b; R_3_ = OH	*Alternanthera brasiliana* (L.) Kuntze	Leaves	Brochado et al. (2003)
37	Quercetin-3-*O*-robinobioside or Quercetin-3-O-rutinoside or Rutin	R_1_ = H; R_2_ = b; R_3_ = OH	*Alternanthera brasiliana* (L.) Kuntze, *Alternanthera littoralis* P.Beauv., *Alternanthera sessilis* (L.) R.Br. ex DC.	Leaves; Aerial parts	Brochado et al. (2003)
					Salvador and Dias, (2004)
					Deladino et al. (2017)
38	Kaempferol-3-*O*-robinobioside or Kaempferol-3-*O*-rutinoside	R_1_ = H; R_2_ = b; R_3_ = H	*Alternanthera brasiliana* (L.) Kuntze	Leaves	Brochado et al. (2003)
39	Isorhamnetin-3-O-robinobioside or Isorhamnetin-3-O-rutinoside	R_1_ = H; R_2_ = b; R_3_ = OCH_3_	*Alternanthera littoralis* P.Beauv., *Alternanthera brasiliana* (L.) Kuntze, *Alternanthera sessilis* (L.) R.Br. ex DC.	Leaves; Aerial parts	Salvador and Dias, (2004)
					Deladino et al. (2017)
40	Kaempferol-rhamnosyl- rhamnosyl-glycoside	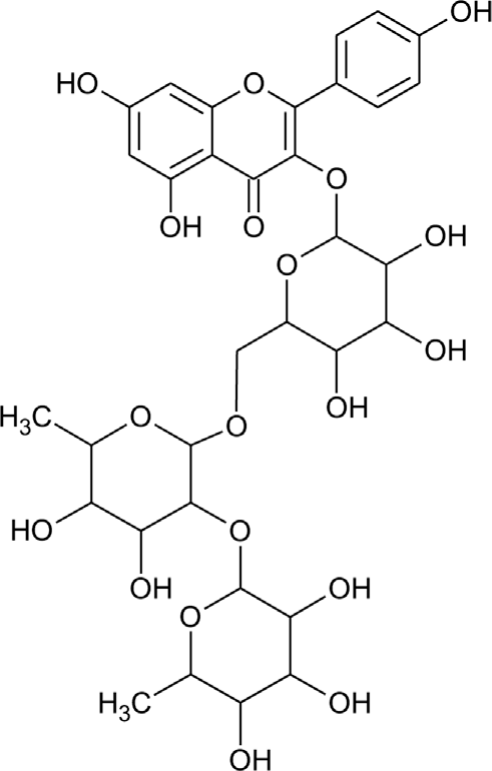	*Alternanthera brasiliana* (L.) Kuntze, *Alternanthera sessilis* (L.) R.Br. ex DC.	Leaves	Deladino et al. (2017)
	Volatile oil				
41	Limonene	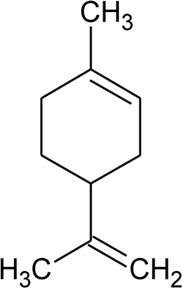	*Alternanthera pungens* Kunth	—	De Ruiz et al. (1993)
42	α-Curcumene	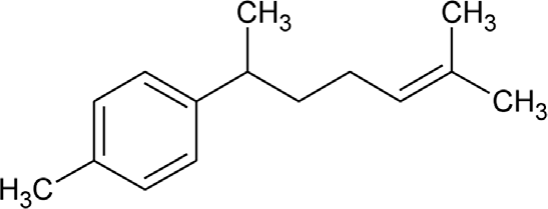	*Alternanthera pungens* Kunth	—	De Ruiz et al. (1993)
43	Geraniol	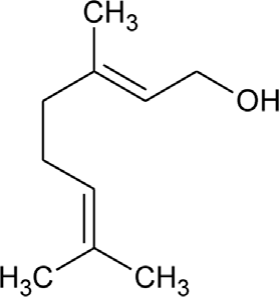	*Alternanthera pungens* Kunth	—	De Ruiz et al. (1993)
44	Linalool	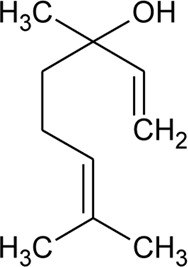	*Alternanthera pungens* Kunth	—	De Ruiz et al. (1993)
45	Camphor	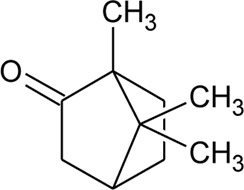	*Alternanthera pungens* Kunth	—	De Ruiz et al. (1993)
46	Myrcene	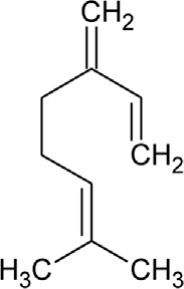	*Alternanthera pungens* Kunth	—	De Ruiz et al. (1993)
47	Camphene	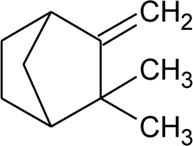	*Alternanthera pungens* Kunth	—	De Ruiz et al. (1993)
48	α-pinene	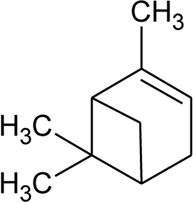	*Alternanthera pungens* Kunth	—	De Ruiz et al. (1993)
	Sterols				
49	Stigmasterol	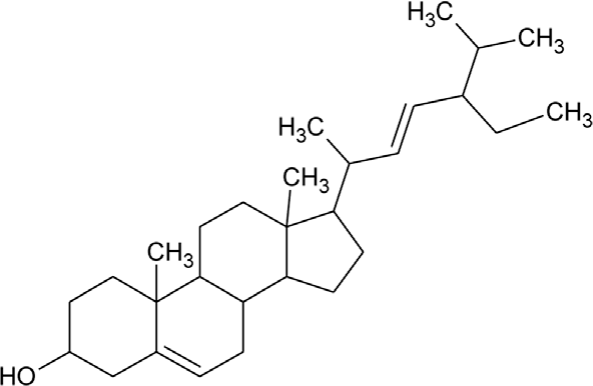	*Alternanthera brasiliana* (L.) Kuntze*, Alternanthera sessilis* (L.) R.Br. ex DC.	Leaves	Pereira et al. (2013)
					Walter et al. (2014)
50	Campesterol	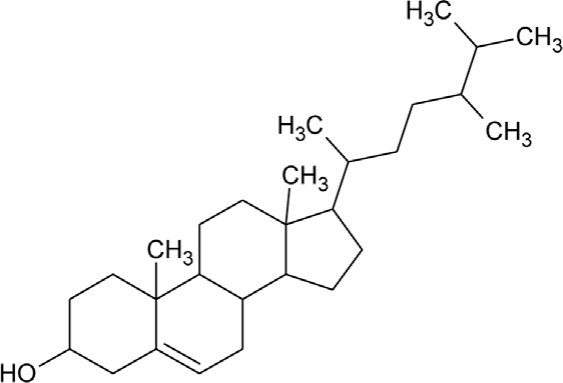	*Alternanthera sessilis* (L.) R.Br. ex DC.	—	Walter et al. (2014)
51	*β*-Sitosterol	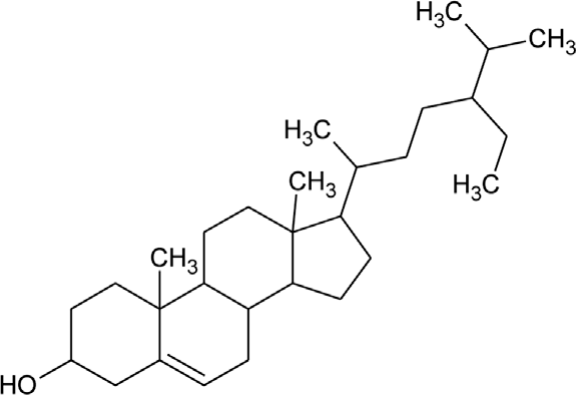	*Alternanthera brasiliana* (L.) Kuntze, *Alternanthera sessilis* (L.) R.Br. ex DC., *Alternanthera philoxeroides* (Mart.) Griseb	Leaves	Fang et al. (2006)
					Gupta and Singh, (2012b)
					Pereira et al. (2013)
	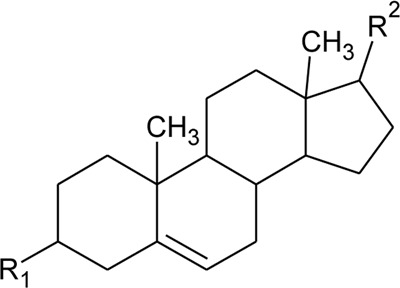		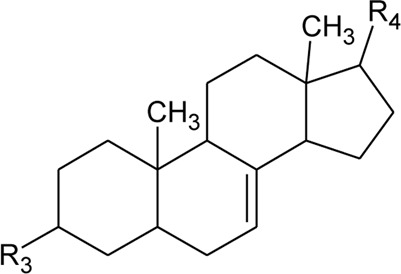		
	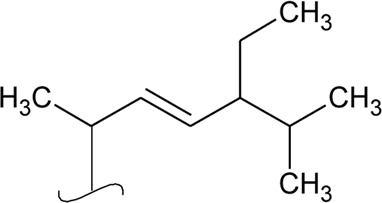 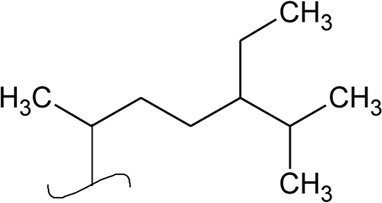		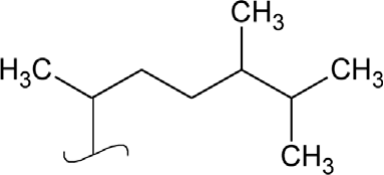		
	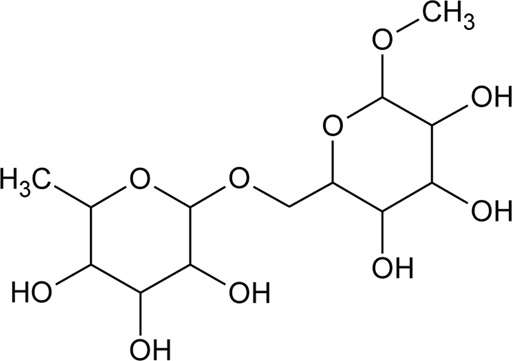				
52	Δ^5^-Stigmasterol or Stigmasteryl or Stigmasta-5, 22-dien-3-*β*-ol	R_1_ = OH; R_2_ = a	*Alternanthera littoralis* P.Beauv., *Alternanthera sessilis* (L.) R.Br. ex DC., *Alternanthera philoxeroides* (Mart.) Griseb	Aerial parts; Whole plant	Salvador and Dias, (2004)
					Fan, (2008)
					Salvador et al. (2009)
53	*β*-Sitosterol	R_1_ = OH; R_2_ = b	*Alternanthera sessilis* (L.) R.Br. ex DC.	Whole plant	Salvador et al. (2009)
54	Campesterol	R_1_ = OH; R_2_ = c	*Alternanthera sessilis* (L.) R.Br. ex DC.	Whole plant	Salvador et al. (2009)
55	Δ^7^-Spinasterol or *α*-Spinasterol	R_3_ = OH; R_4_ = a	*Alternanthera brasiliana* (L.) Kuntze, *Alternanthera sessilis* (L.) R.Br. ex DC., *Alternanthera philoxeroides* (Mart.) Griseb	Aerial parts; Whole plant	Salvador and Dias, (2004)
					Fang et al. (2006)
					Fan, (2008)
					Salvador et al. (2009)
					Pereira et al. (2013)
					Walter et al. (2014)
56	Δ^7^-Stigmasterol or Stigmast-7en-3-*β*-ol	R_3_ = OH; R_4_ = b	*Alternanthera littoralis* P.Beauv.*, Alternanthera sessilis* (L.) R.Br. ex DC.	Aerial parts; Whole plant	Salvador and Dias, (2004)
					Salvador et al. (2009)
57	Stigmast-7enyl-3-*β*-ol-3-*O*-*β*-D-glucopyranoside or 3-*O*-*β*-D-Glucopyranosyl *β*-sitosterol	R_1_ = O-Glu; R_2_ = b	*Alternanthera littoralis* P.Beauv., *Alternanthera sessilis* (L.) R.Br. ex DC.	Aerial parts; Whole plant	Salvador and Dias, (2004)
					Salvador et al. (2009)
58	3-*O*-*β*-D-Glucopyranosyl stigmasterol	R_1_ = O-Glu; R_2_ = a	*Alternanthera sessilis* (L.) R.Br. ex DC.	Whole plant	Salvador et al. (2009)
59	3-*O*-*β*-D-Glucopyranosyl Δ^7^-stigmasterol	R_3_ = O-Glu; R_4_ = b	*Alternanthera sessilis* (L.) R.Br. ex DC.	Whole plant	Salvador et al. (2009)
60	3-*O*-*β*-D-Glucopyranosyl spinasterol	R_3_ = O-Glu; R_4_ = a	*Alternanthera sessilis* (L.) R.Br. ex DC.	Whole plant	Salvador et al. (2009)
61	6S,7E,9R-6,9-Di-hydroxymegastigma-4,7-dien-3-one-9-O-beta-D-glucopyranoside	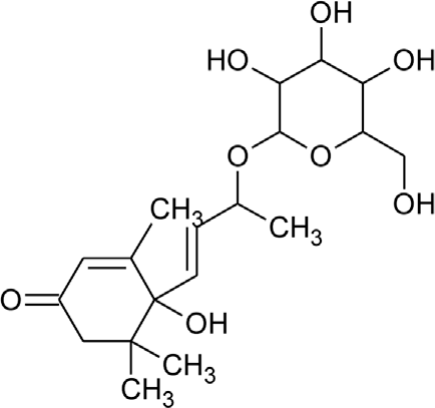	*Alternanthera philoxeroides* (Mart.) Griseb	—	Fang et al. (2009b)
62	3*β*-Hydroxystigmast-5-en-7-one	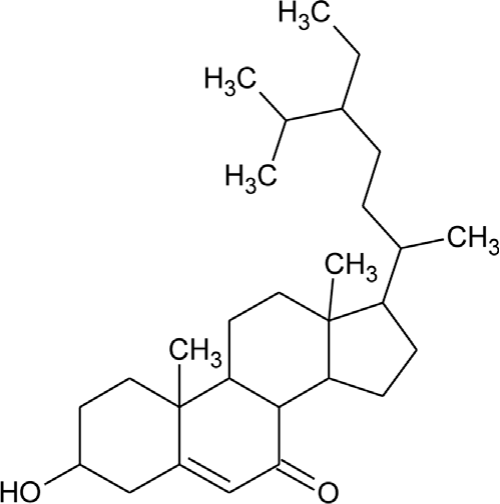	*Alternanthera brasiliana* (L.) Kuntze, *Alternanthera sessilis* (L.) R.Br. ex DC.	Leaves	Deladino et al. (2017)
	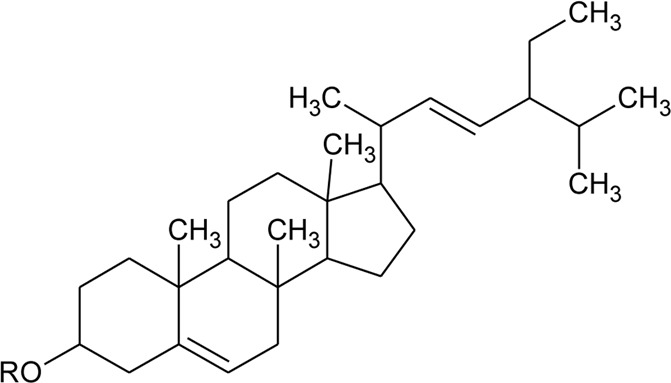				
63	Sitosterol-3-*O*-*β*-D-glucopyranoside	R = *β*-D-glucopyranoside	*Alternanthera brasiliana* (L.) Kuntze	Flowers	Facundo et al. (2012)
	Triterpenoid/Saponins				
64	Ursolic acid	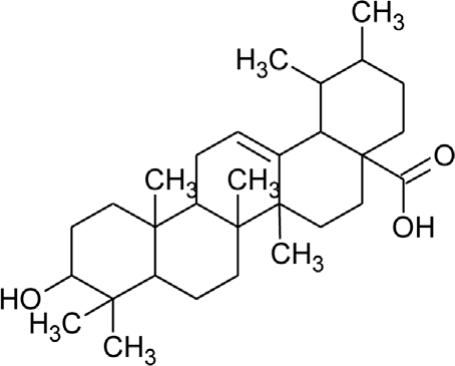	*Alternanthera philoxeroides* (Mart.) Griseb	Aerial parts	Fan, (2008)
65	Oleanolic acid 28-O-beta-D-glucopyranoside	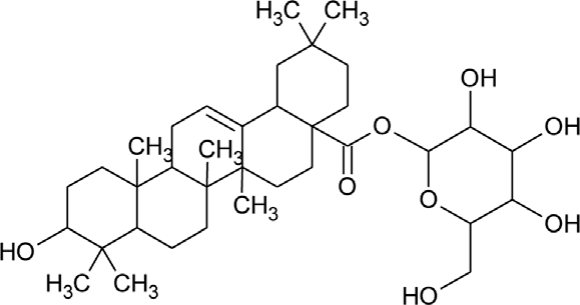	*Alternanthera philoxeroides* (Mart.) Griseb	—	Fang et al. (2009b)
	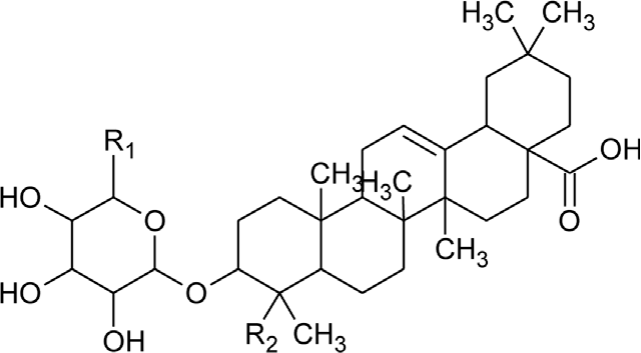				
66	Oleanolic acid 3-O-beta-D-glucuronopyranoside-6′-O-methyl ester	R_1_ = CH_3_COO; R_2_ = CH_3_	*Alternanthera philoxeroides* (Mart.) Griseb	—	Fang et al. (2009b)
67	Hederagenin 3-O-beta-D-glucuronopyranoside-6′-O-methyl ester	R_1_ = CH_3_COO; R_2_ = CH_2_OH	*Alternanthera philoxeroides* (Mart.) Griseb	—	Fang et al. (2009b)
68	Hederagenin-3-O-beta-D-glucuronopyranoside (HN-Saponin K)	R_1_ = R_2_ = CH_2_OH	*Alternanthera philoxeroides* (Mart.) Griseb	—	Guo et al. (2011)
69	Philoxeroideside A	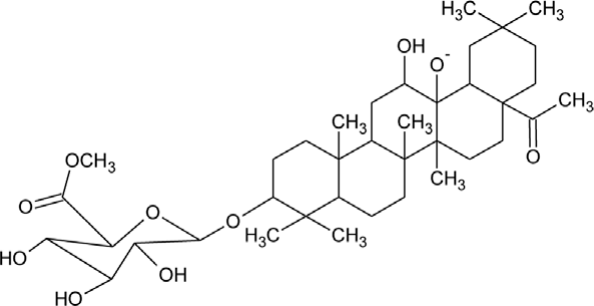	*Alternanthera philoxeroides* (Mart.) Griseb	Aerial parts	Fang et al. (2009a)
70	Philoxeroideside B	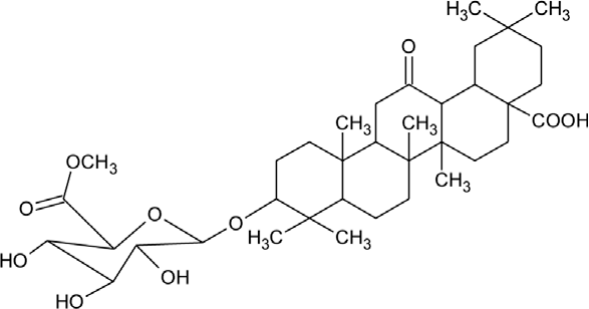	*Alternanthera philoxeroides* (Mart.) Griseb	Aerial parts	Fang et al. (2009a)
71	Philoxeroideside C	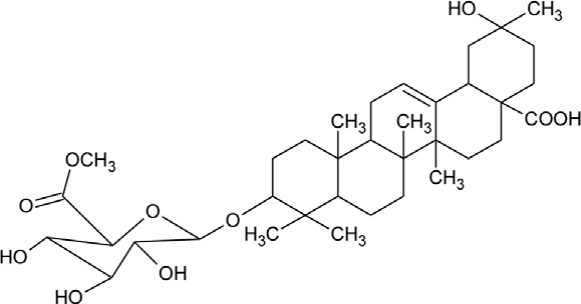	*Alternanthera philoxeroides* (Mart.) Griseb	Aerial parts	Fang et al. (2009a)
72	Philoxeroideside D	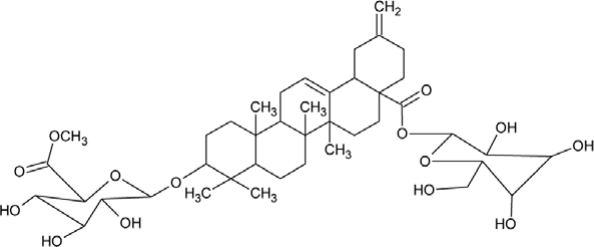	*Alternanthera philoxeroides* (Mart.) Griseb	Aerial parts	Fang et al. (2009a)
	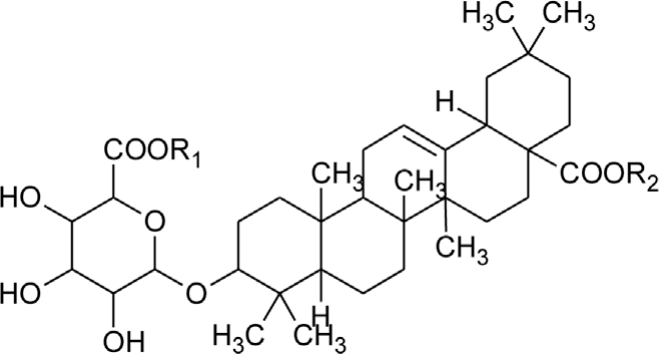 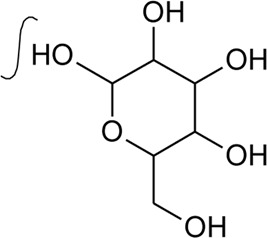				
73	Chikusetsusaponin IVa or Oleanolic acid-3-O-beta-D-glucopyranosyl-28-Obeta-D-glucopyranosyl ester	R_1_ = H; R_2_ = a	*Alternanthera philoxeroides* (Mart.) Griseb	Whole plant	Rattanathongkom et al. (2009)
74	Chikusetsusaponin IV a methyl ester	R_1_ = CH_3_; R_2_ = a	*Alternanthera philoxeroides* (Mart.) Griseb	—	Fang et al. (2009b)
75	Oleanolic acid 3-O-beta-D-glucuronopyranoside or Calenduloside E	R_1_ = R_2_ = H	*Alternanthera philoxeroides* (Mart.) Griseb	Whole plant	Fang et al. (2009b)
					Rattanathongkom et al. (2009)
					Guo et al. (2011)
76	Oleanolic acid	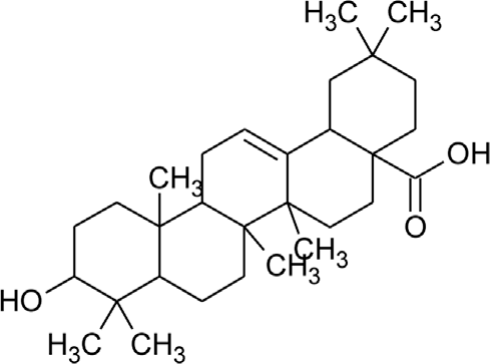	*Alternanthera philoxeroides* (Mart.) Griseb	—	Fang et al. (2006)
	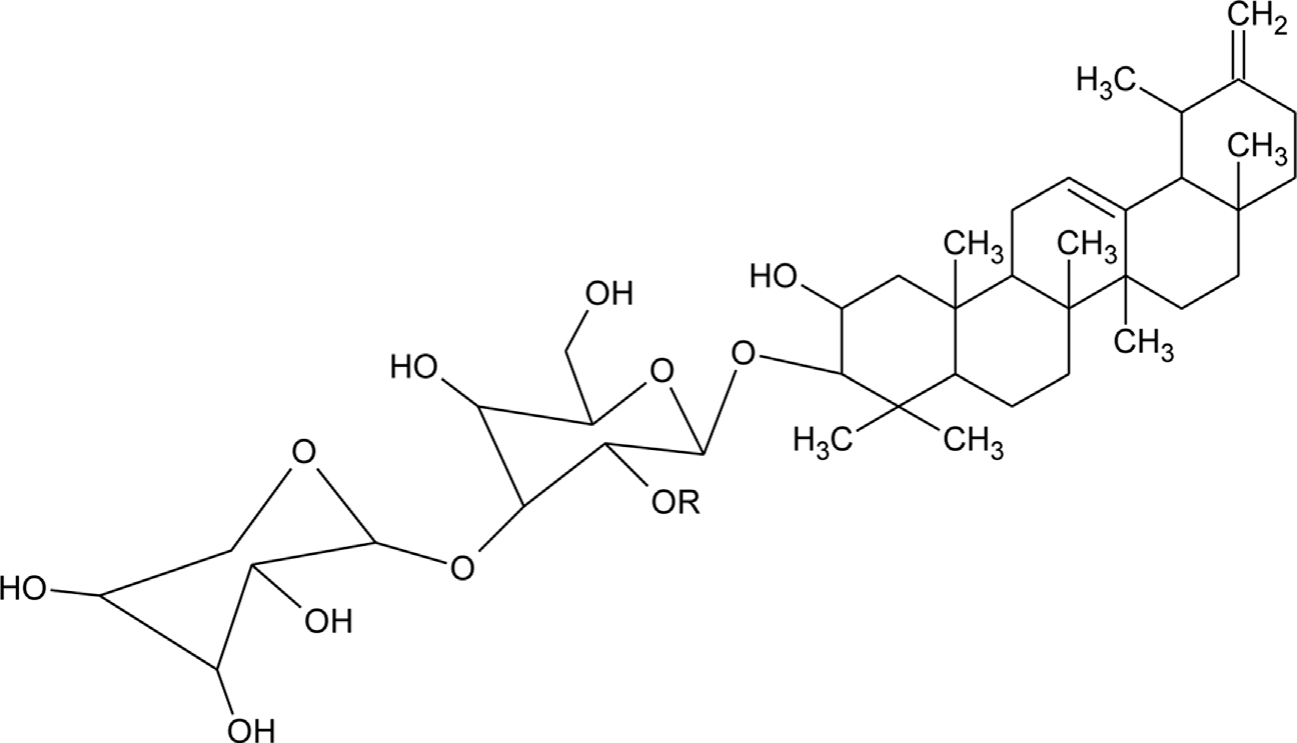				
77	2α, 3β-dihydroxyurs-12,20(30)-dien-28-oic acid	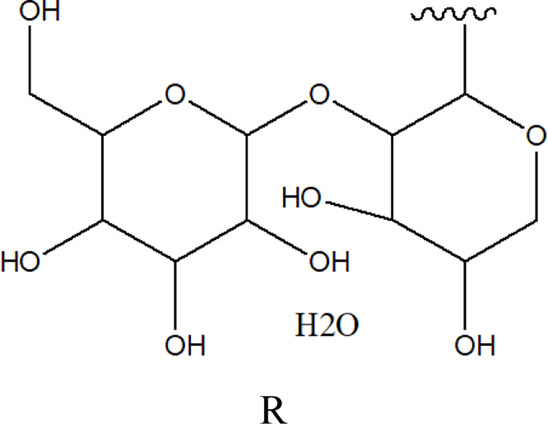	*Alternanthera sessilis* (L.) R.Br. ex DC.	Aerial parts	Sanoko et al. (1999)
78	2α,3β-dihydroxy urs-12,20(30)-dien-28-oic acid 3-*O*-{*O*-*β* -D-quinovopyranosyl-(1→2)-*O*-α-L- arabinopyranosyl- (1→2)-O-[β-D- xylopyranosyl-(1→3)] *β*-D-glucopyranoside}	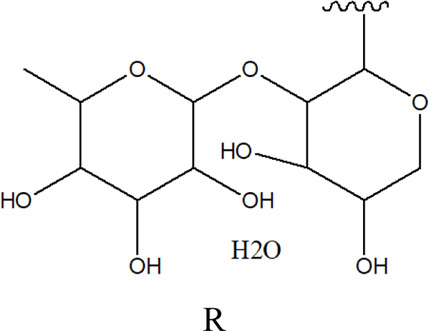	*Alternanthera sessilis* (L.) R.Br. ex DC.	Aerial parts	Sanoko et al. (1999)
79	2α,3β-dihydroxy urs-12,20(30)-dien-28-oic acid 3-*O*-{*O*-*α* -L- arabinopyranosyl -(1→2)-*O*-[β-D- xylopyranosyl-(1→3)] *β*-D-glucopyranoside}	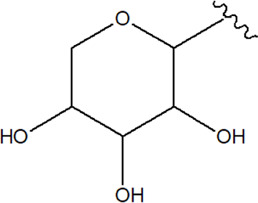 R	*Alternanthera sessilis* (L.) R.Br. ex DC.	Aerial parts	Sanoko et al. (1999)
80	2α,3β-dihydroxy urs-12,20(30)-dien-28-oic acid 3-*O*-{[*O*-*β*-D- xylopyranosyl-(1→3)] *β*-D-glucopyranoside}	R = H	*Alternanthera sessilis* (L.) R.Br. ex DC.	Aerial parts	Sanoko et al. (1999)
	Phenolic compounds				
81	Ellagic acid	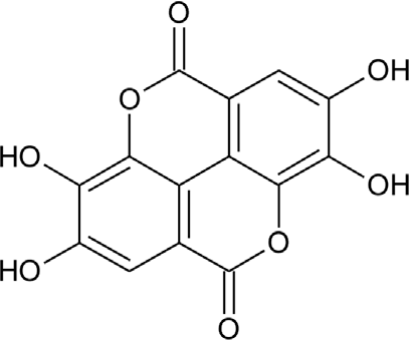	*Alternanthera sessilis* (L.) R.Br. ex DC.	Whole plant	Mondal et al. (2015)
82	Caffeic acid	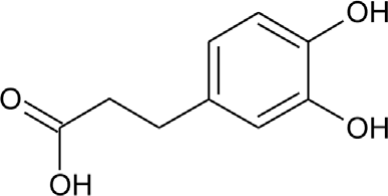	*Alternanthera philoxeroides* (Mart.) Griseb., *Alternanthera hirtula* (Mart.) R.E.Fr., *Alternanthera praelonga* A.St.-Hil	Whole plant	Correa et al. (2016)
83	Quinic acid	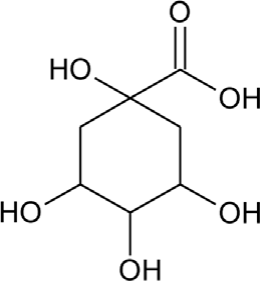	*Alternanthera philoxeroides* (Mart.) Griseb., *Alternanthera hirtula* (Mart.) R.E.Fr., *Alternanthera praelonga* A.St.-Hil	Whole plant	Correa et al. (2016)
84	Ferulic acid	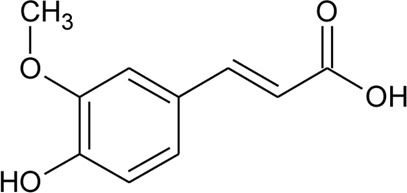	*Alternanthera brasiliana* (L.) Kuntze, *Alternanthera sessilis* (L.) R.Br. ex DC., *Alternanthera hirtula* (Mart.) R.E.Fr., *Alternanthera praelonga* A.St.-Hil	Whole plant; leaves	Correa et al. (2016)
					Deladino et al. (2017)
85	*p*-Coumaric acid	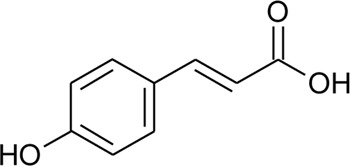	*Alternanthera brasiliana* (L.) Kuntze, *Alternanthera sessilis* (L.) R.Br. ex DC., *Alternanthera philoxeroides* (Mart.) Griseb	Leaves; Aerial parts	Fan, (2008)
					Deladino et al. (2017)
86	4-Hydroxybenzoic acid	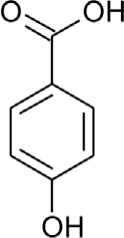	*Alternanthera brasiliana* (L.) Kuntze, *Alternanthera sessilis* (L.) R.Br. ex DC.	Leaves	Deladino et al. (2017)
87	2,5-Dihydroxybenzoic acid or gentisic acid	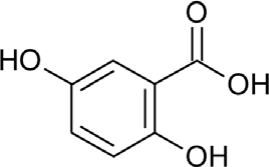	*Alternanthera brasiliana* (L.) Kuntze, *Alternanthera sessilis* (L.) R.Br. ex DC.	Leaves	Deladino et al. (2017)
88	Hydroxytyrosol	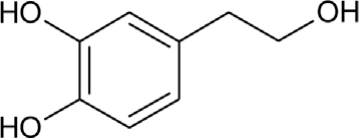	*Alternanthera littoralis* P.Beauv	Aerial parts	Koolen et al. (2017)
89	Chlorogenic acid	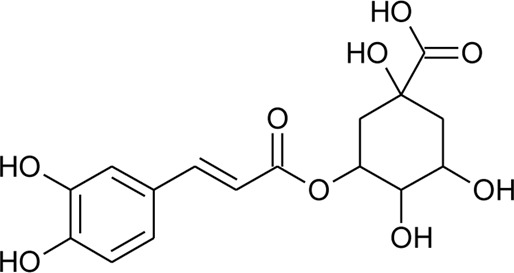	*Alternanthera brasiliana* (L.) Kuntze, *Alternanthera sessilis* (L.) R.Br. ex DC.	Leaves	Deladino et al. (2017)
90	2,5-Dihydroxybenzoic acid 5-O-*β*-D-glucoside	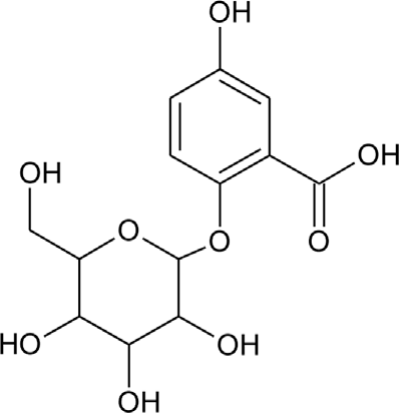	*Alternanthera brasiliana* (L.) Kuntze, *Alternanthera sessilis* (L.) R.Br. ex DC.	Leaves	Deladino et al. (2017)
	Ionone				
91	Ionone (Alcoholic derivative)	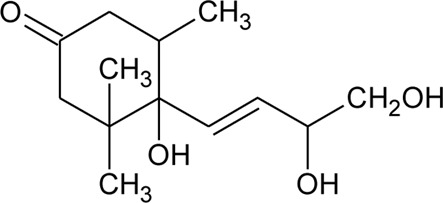	*Alternanthera sessilis* (L.) R.Br. ex DC.	Leaves	Ragasa et al. (2010)
92	α-Ionone	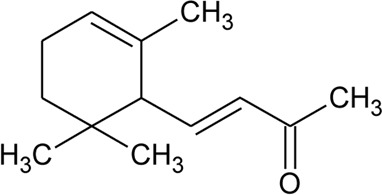	*Alternanthera sessilis* (L.) R.Br. ex DC.	Leaves	Ragasa et al. (2010)
93	Ionone (Aldehyde derivative)	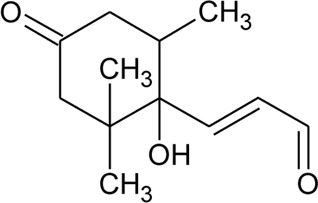	*Alternanthera sessilis* (L.) R.Br. ex DC.	Leaves	Ragasa et al. (2010)
	Anthraquinone				
	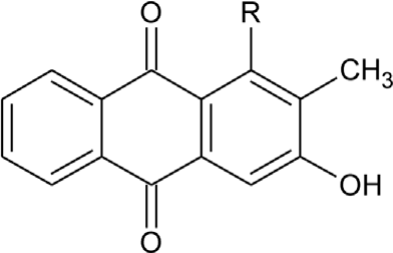				
94	Rubiadin	R = OH	*Alternanthera philoxeroides* (Mart.) Griseb	Aerial parts	Fan, (2008)
					Collett and Taylor, (2019)
95	Rubiadin l-methyl ether	R = OCH_3_	*Alternanthera philoxeroides* (Mart.) Griseb	Aerial parts	Fan, (2008)
96	2-Hydroxy-3-methylanthraquinone	R = H	*Alternanthera philoxeroides* (Mart.) Griseb	Aerial parts	Fan, (2008)
97	Rhein	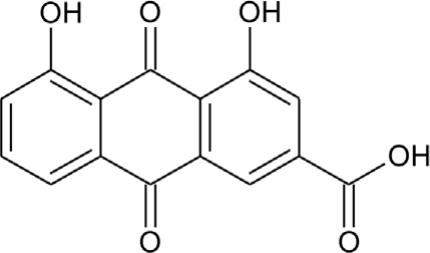	*Alternanthera pungens* Kunth	Flowers	Gupta and Saxena, (1987)
	Hydroxycinnamic acids				
	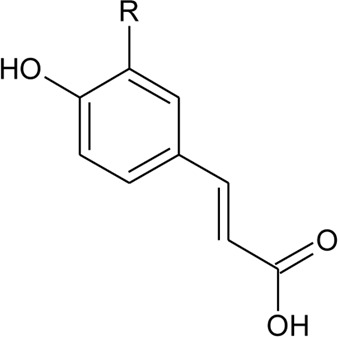				
98	(E)-3-(4-hydroxyphenyl)prop-2-enoic acid	R = H	*Alternanthera bettzickiana* (Regel) G.Nicholson	Leaves	Petrus et al. (2014a)
99	(E)-3-(3,4-dihydroxyphenyl) prop-2-enoic acid	R = OH	*Alternanthera bettzickiana* (Regel) G.Nicholson	Leaves	Petrus et al. (2014a)
100	(E)-3-(4-hydroxy-3-methoxyphenyl) prop-2-enoic acid	R = OCH_3_	*Alternanthera bettzickiana* (Regel) G.Nicholson	Leaves	Petrus et al. (2014a)
	Alkaloids				
101	Alternamide A (7,8-dihydroxy-1,2,4,5-tetrahydro-3H -1,5-ethano[c]azepin-3-one)	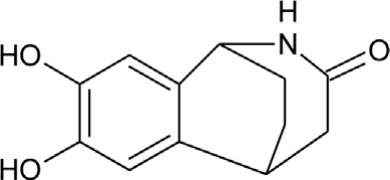	*Alternanthera littoralis* P.Beauv	Aerial parts	Koolen et al. (2017)
102	Alternamide B (6,7-dihydroxy-3,4- dihydroquinoline-1-one)	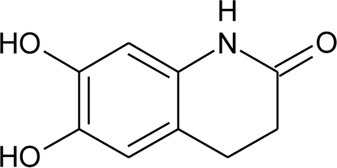	*Alternanthera littoralis* P.Beauv	Aerial parts	Koolen et al. (2017)
103	Alternamine A [(R)-1-(3,4-dihydroxyphenyl)-1,2,3,4-tetrahydroisoquinoline-6,7-diol)]	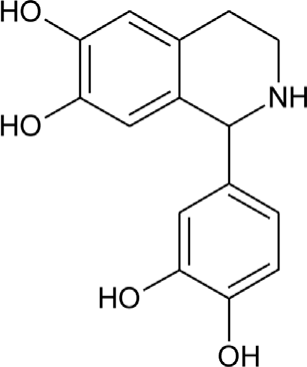	*Alternanthera littoralis* P.Beauv	Aerial parts	Koolen et al. (2017)
104	N -(3,4-Dihydroxyphenethyl) formamide	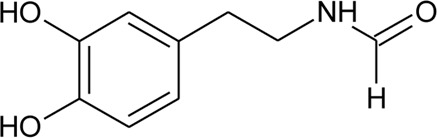	*Alternanthera littoralis* P.Beauv	Aerial parts	Koolen et al. (2017)
105	Alternamine B {4-(2-aminoethyl) benzene-1,2-diol-4-(2-aminoethyl)benzene-1,2-diol-b -D –glucopyranose}	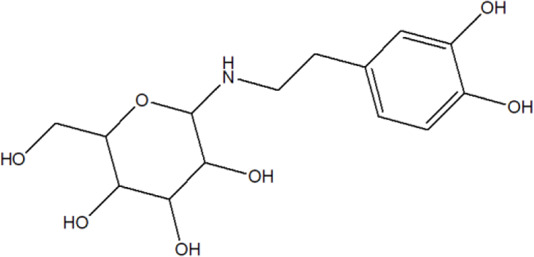	*Alternanthera littoralis* P.Beauv	Aerial parts	Koolen et al. (2017)
106	Uridine	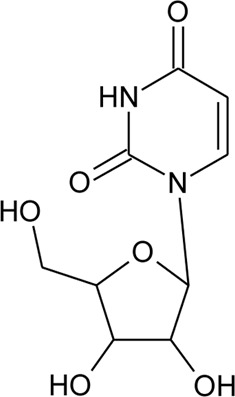	*Alternanthera littoralis* P.Beauv	Aerial parts	Koolen et al. (2017)
107	N-trans-feruloyl tyramine	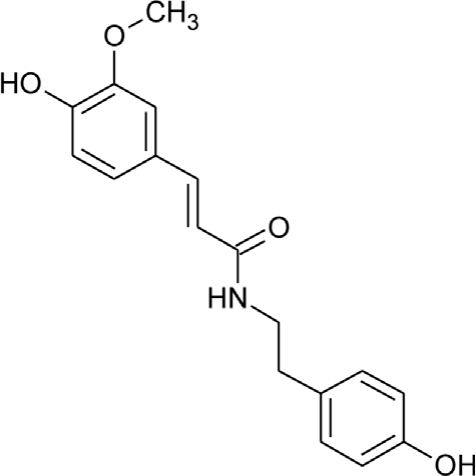	*Alternanthera philoxeroides* (Mart.) Griseb	Aerial parts	Fan, (2008)
	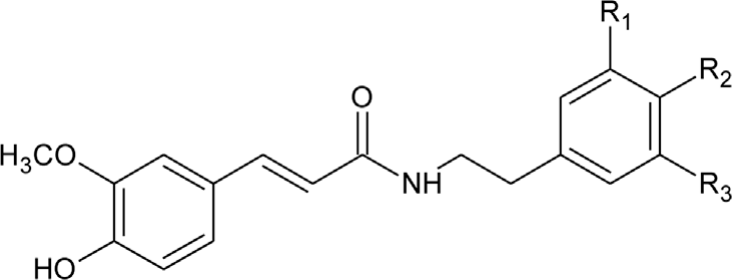				
108	N-trans-feruloyl-3,5-dimethoxytyramine	R_1_ = OCH_3_; R_2_ = OH; R_3_ = OCH_3_	*Alternanthera philoxeroides* (Mart.) Griseb	Aerial parts	Fang et al. (2007)
109	N-trans-feruloyl-3-methyldopamine	R_1_ = OCH_3_; R_2_ = OH; R_3_ = H	*Alternanthera philoxeroides* (Mart.) Griseb	Aerial parts	Fang et al. (2007)
110	N-trans-feruloyl tyramine	R_1_ = H; R_2_ = OH; R_3_ = H	*Alternanthera philoxeroides* (Mart.) Griseb	Aerial parts	Fang et al. (2007)
111	N-cis-feruloyl tyramine	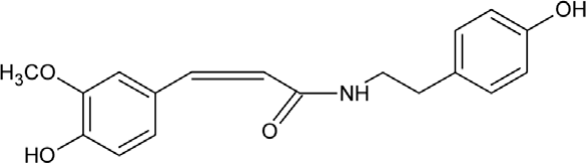	*Alternanthera philoxeroides* (Mart.) Griseb	Aerial parts	Fang et al. (2007)
112	*β*-Carboline	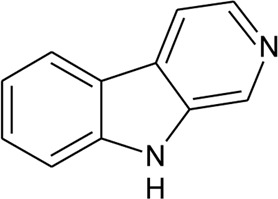	*Alternanthera philoxeroides* (Mart.) Griseb	Leaves	Zhang et al. (2018)
	Miscellaneous				
113	*β*-Carotene	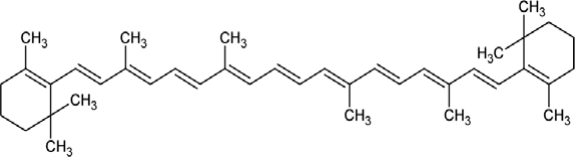	*Alternanthera sessilis* (L.) R.Br. ex DC.	—	Walter et al. (2014)
114	Ricinoleic acid	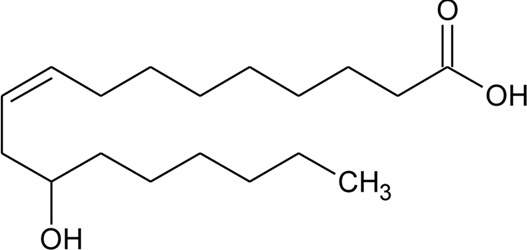	*Alternanthera sessilis* (L.) R.Br. ex DC.	Seeds	Hosamani et al. (2004)
115	Malic acid	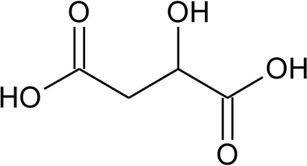	*Alternanthera philoxeroides* (Mart.) Griseb., *Alternanthera hirtula* (Mart.) R.E.Fr	Leaves	Correa et al. (2016)
	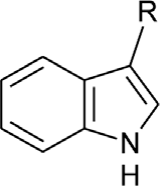				
116	Indole-3-carboxaldehyde	R = CHO	*Alternanthera philoxeroides* (Mart.) Griseb	Aerial parts	Fan, (2008)
117	Indole-3-carboxylic acid	R = COOH	*Alternanthera philoxeroides* (Mart.) Griseb	Aerial parts	Fan, (2008)
118	Azelaic acid	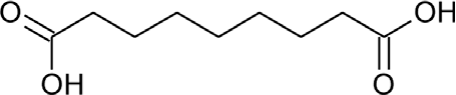	*Alternanthera philoxeroides* (Mart.) Griseb	Aerial parts	Fan, (2008)
119	Blumenol A	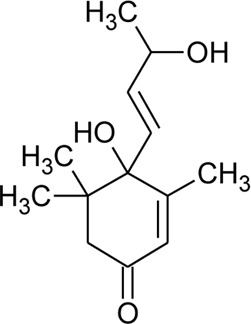	*Alternanthera philoxeroides* (Mart.) Griseb	Aerial parts	Fan, (2008)
120	4,5-Dihydroblumenol	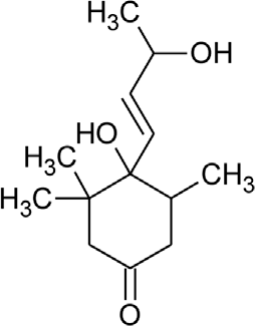	*Alternanthera philoxeroides* (Mart.) Griseb	—	Fang et al. (2009b)
121	Cycloeucalenol	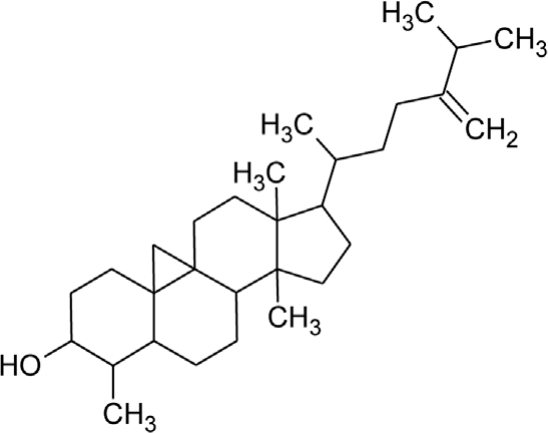	*Alternanthera philoxeroides* (Mart.) Griseb	—	Fang et al. (2006)
122	Phytol	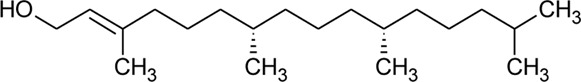	*Alternanthera philoxeroides* (Mart.) Griseb	—	Fang et al. (2006)
123	Phaeophytin A	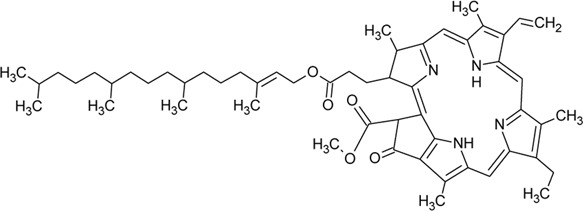	*Alternanthera philoxeroides* (Mart.) Griseb	—	Fang et al. (2006)
124	Pheophytin A	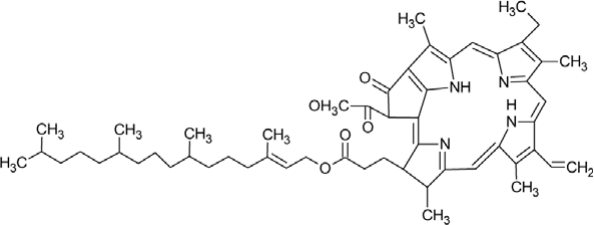	*Alternanthera philoxeroides* (Mart.) Griseb	—	Fang et al. (2006)
125	24-Methylene-cycloartanol	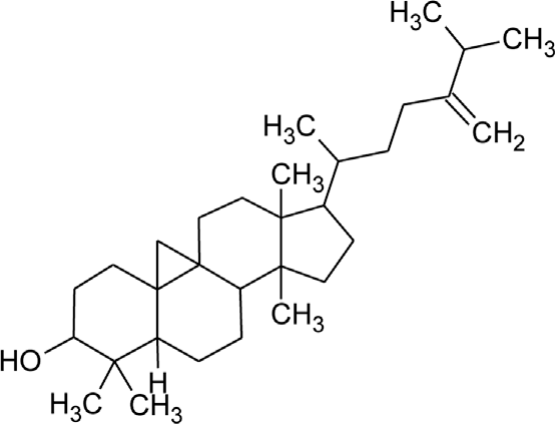	*Alternanthera brasiliana* (L.) Kuntze, *Alternanthera sessilis* (L.) R.Br. ex DC.	Leaves	Deladino et al. (2017)
	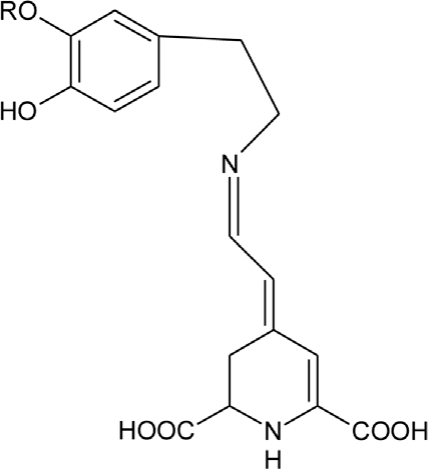				
126	Dopamine-betaxanthin	R = H	*Alternanthera brasiliana* (L.) Kuntze, *Alternanthera sessilis* (L.) R.Br. ex DC.	Leaves	Deladino et al. (2017)
127	3-Methoxytyramine-betaxanthin	R = CH_3_	*Alternanthera brasiliana* (L.) Kuntze, *Alternanthera sessilis* (L.) R.Br. ex DC.	Leaves	Deladino et al. (2017)
128	Choline	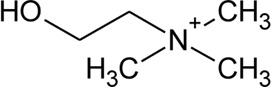	*Alternanthera pungens* Kunth	—	De Ruiz et al. (1993)
129	Leucoantocyanidin	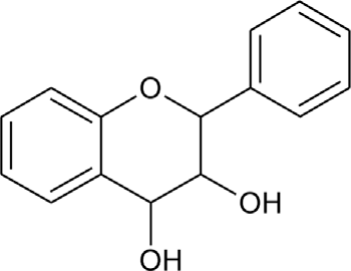	*Alternanthera pungens* Kunth	Aerial parts	Petrus and Seetharaman, (2005)

The present review emphasizes traditional uses, chemical constituents, pharmacological actions, clinical potential, and safety profile of *Alternanthera* species. The current work has been compiled to fulfill the following goals: 1) to explore if traditional claims of *Alternanthera* species have been scientifically justified by pharmacological and clinical studies, and also to assess critically if their mechanism of actions is established, 2) to explore whether detailed phytochemical investigations have been conducted to detect and isolate main/bioactive constitutes of various species, 3) to reveal whether appropriate analytical methods have been developed for standardization of plant materials based on marker compounds, 4) to analyze whether isolated compounds from *Alternanthera* species have potential to be developed as lead molecules unaltered or needs derivatization to develop semisynthetic drugs through proper SAR studies and 5) to check if the safety and toxicity profiles of *Alternanthera* species have been studied. The scattered raw data has been compiled from online databases such as SciFinder, Google Scholar, PubMed, Science Direct, and Open J-Gate for 100 years up to April 2021 and offline databases such as Aromatic Plants Abstract, scientific journals, and books from different libraries of National repute. Keywords selected were based on various species of Alternanthera genus, and different biological activities. The articles which were in English and available with full text were included. Manuscript written in non-English versions were excluded. A total of 156 articles related to Alternanthera genus were finally studied and cited. But the cross-sectional literature review led us to cover a total of around 500 articles in this review article. The review article is categorized into six sections: 1) morphology emphasizes morphological characters of different *Alternanthera* species; 2) ethnopharmacology covers traditional uses of different *Alternanthera* species; 3) phytoconstituents includes name and structure of chemicals constituents isolated from various species of the genus; 4) biological activities focus on different pharmacological activities reported in various species and presented in the table; 5) toxicity studies include scientific reports of toxicity studies of different *Alternanthera* species and 6) clinical studies describe clinical trials conducted on humans.

### Morphology

The morphological profile of various species of the genus was found to be similar with some variations. *A. brasiliana* (L.) Kuntze (a perennial herb mainly distributed in Brazil) is prostrate, 7.5–45 cm long branches, introducing a round stem, long internodes, and swollen nodes, at which inverse leaves connect (Kumar S. et al., 2011). Branches are glabrous, two lines of hair, nodes frequently villous; leaves are 2.5–7.5 cm, considerably longer when developing in watery spots, rather plump, at some point indefinitely denticulate; flowers are white, found in the form of bunches; seeds are 1.25–1.5 mm, sub-orbicular.

*A. denticulata* R. Br. and *A. nahui* Heenan and de Lange comprise stem of 100 mm height and located in an upright position (Heenan et al., 2009). The uniform spreading of minute hairs is present on the stems of both plants. The dark green-colored leaves (length—30 mm and breadth—6 mm) of both plants are linear, entire, narrow, elliptic, denticulate margins, and oblong in appearance. The abaxial surface of the tepals (length: 2.0–4.2 mm) is described by keeled, a character that is presented at the base of mature and dried tepals.

*A. philoxeroides* (Mart.) Griseb.*,* a perennial herb, has stems crawling or gliding rising towards pinnacle, establishing at the lower hubs, branched, empty, with a longitudinal hairy groove score on two inverse sides (Pulipati et al., 2015). The fresh and delicious stems can develop on a level plane and float on the outside of the water, framing pontoons, or structure tangled bunches that develop onto banks. The leaves are inverse two by two, with an unmistakable midrib, and ranges from 5–10 cm. The plant consists of leaf, lanceolate shape, intense pinnacle, whole edge, glabrous surface, graduate base, and short strong petiole.

*A. pungens* Kunth is a perennial herb with a stem of 10–15 cm long with hair. The leaves are green in color and ovate in a shape of about 0.5–4.5 cm long and 0.3–2 cm in width (Naidu, 2012). It is native to the Southern American continent generally found in South Carolina, Florida, and California spreading around the road sides (Gupta et al., 2012). In 1918 it was first reported in the Southern parts of India (Rao, 2000).

*A. sessilis* (L.) R.Br. ex DC. is a perennial herb with purple-colored and glabrous branches grown from the root bases about 50 cm in length (Anitha and Kanimozhi, 2012). The fresh leaves are shiny, 1.3–3.0 cm long and 0.5–1.0 cm wide however the leaves are bigger in wet living spaces, direct elliptic, oval or obovate, zenith adjusted and base cuneate. The blossoms are subtle, white, borne in little, axillary heads; bracts are obovate and 1 mm long. The bracteoles are shorter, persevering; subequal, and intense. Utricleare cordi-structure and are unequivocally compacted. The seeds are orbicular. The plant bears blossoms and natural products consistently.

### Ethnopharmacology and Traditional Uses

The infusion of inflorescences of *A. Brasiliana* (L.) Kuntze with water is used in headaches, coughs, colds, and grippe (Hundiwale et al., 2012). The infusion of leaves with a cup of water has been used in the treatment of fever while a decoction of roots is used in diarrhea. Traditionally, the various plant parts (stems, leaves, flowers, roots) of *A. caracasana* Kunth have been used to treat dysentery, diarrhea, and fever. The infusion of the plant is used as lavage or beverage in the traditional system of medicines (Canales-Martínez et al., 2008). The aerial parts of *A. Brasiliana* (L.) Kuntze are indicated in the treatment of inflammation, pain, and various infections (Hundiwale et al., 2012). The leaves of *A. ficoidea* (L.) P.Beauv. has been used in the treatment of heart and cancer problems (Patil and Kore, 2019). *A. littoralis* P. Beauv. has a long tradition of use in the treatment of infectious and inflammatory diseases (Koolen et al., 2017). The old texts indicated the use of *A. littoralis* P. Beauv. in the treatment of inflammatory, infectious diseases (de Santana Aquino et al., 2015), viral infections, immunity problems, cancer, malaria, and diarrhea (Hundiwale et al., 2012; Sekar, 2012). *A. nodiflora* R.Br. has been in the treatment of skin, degenerative and microbial infections (Feka et al., 2014). *A. paronychioides* A.St.-Hil. has been used in the treatment of hyperuricemia, rheumatic arthritis, uremia, nephritis, gout, cystitis, diabetes, and systemic neuralgia in TCM (Wu et al., 2013). In Ayurveda, the syrup of the whole plant of *A. philoxeroides* (Mart.) Griseb. has been employed in the treatment of influenza (Hundiwale et al., 2012). The aqueous infusion of leaf and flower of *A. porrigens* (Jacq.) Kuntze has been recorded in old texts for the treatment of hepatic pain, kidney problems, and influenza. *A. pungens* Kunth has been employed as folk medicine in Argentina, commonly known as Yerba del pollo, recorded in the Pharmacopeia National Argentina (1978) for various medicinal purposes. It has been traditionally used in the treatment of swelling, nasopharyngeal infections, as a painkiller in labor pain, and also for lactation stimulus in veterinary-related cases (Burkill, 1985). It is also used in the treatment of gonorrhea (Semenya and Potgieter, 2014), menstrual disorder, miscarriage (Lucky and Diame, 2010) and to treat dysentery, cholera, and many parasitic diseases (Grønhaug et al., 2008; Guede et al., 2010). In Sudan, it is used in aqueous form for the treatment of cough. In Brazil, the aerial parts are used against grippe and vermifuge (Agra et al., 2007). It is used for crushing kidney stones or renal calculi in the form of decoction. The whole plant of *A. sessilis* (L.) R.Br. ex DC. has been used as green vegetable for maintain the nutrient balance in body (Astudillo-Vázquez et al., 2008). The roasted leaves and stems (*p.o.*) of *A. sessilis* (L.) R.Br. ex DC. have been in the treatment of stomach pain, ulcer, and gastric problems (Kumar S. M. et al., 2011). The aerial parts of *A. sessilis* (L.) R.Br. ex DC. have been used as a diuretic in the Ayurvedic system of medicines (Hundiwale et al., 2012). The leaves of *A. sessilis* (L.) R.Br. ex DC. are used as a diuretic, antipyretic and antiseptic and roots are used as amenorrhea, inflammations, ovarian diseases, and female sterility. The young shoots of *A. sessilis* (L.) R.Br. ex DC. have been used as lactagogue and febrifuge (Hosamani et al., 2004). Keeping these in mind, the most common traditional uses for the Alternanthera species were recorded for the treatment and management of inflammation, pain, infectious diseases, and gastric problems.

### Phytoconstituents Isolated and Identified in *Alternanthera* Species

GC–MS of n-hexane extract of *A. philoxeroides* (Mart.) Griseb. leaves showed the presence of 25 compounds. Among this Acetic acid, 2-(2-methoxycarbonylamino-5-nitrophenylthio)-, methyl ester (31.92%); 1,4-Benzenediol, 2,5-bis(1,1-dimethylethyl) (15.06%); 4-Pyridinecarboxamide, 6-bromo-4,5-dicyano-1,2,3,4-tetrahydro-3,3-dimethyl-2-[[(1methylethyamino] oxy] (8.53%); L-Cysteine, N-(trifluoroacetyl)-, butyl ester, trifluoroacetate (ester) (6.59%); Cyclopentaneundecanoic acid, methyl ester (5.4%) and 3-Bromo-N-(2-thiazolyl) benzamide (3.49%) are dominant (Akbar et al., 2021). LC-MS/MS and GC-MS analysis of an ethanolic extract of *A. brasiliana* (L.) Kuntze aerial parts were performed (Alencar Filho et al., 2019). Five compounds (luteolin-8-C-rhamnosylglucoside, 2″-O-rhamnosylvitexin, 2″-O-rhamnosyl-6-C-glucosyl methyl-luteolin, rutin, and 2″-O-rhamnosylswertisin) were identified by LC-MS/MS whereas twenty-two compounds were identified by GC-MS but major proportions were n-hexadecanoic acid with 16.61% followed by linoleic acid, clionasterol, α-tocopherol, stigmast-7-en-3-ol, and α-amyrin. The GC-MS analysis of volatile oil obtained from leaves of *A. pungens* Kunth showed the presence of 12 compounds and the major compound was β-ionone (42.18%) (Ogunmoye et al., 2020). Other compounds identified were Hexahydrofarnesyl acetone (15.53%), Methyl palmitate (6.13%), 1-Octadecyne (4.72%), Undecane (3.73%), p-Metha-1,3,8-triene (3.65%), Isophytol (3.21%), δ-Cadinene (3.06%), 1,2-Dimethyl cyclooctene (3.05%), p-Cymene (2.96%), Phytol (2.67%) and Neophytadiene (2.50%).

The phytoconstituents—benzopyran, flavonoids, volatile oil, sterols, triterpenoid/saponins, phenolic compounds, ionone, anthraquinone, hydroxycinnamic acids, alkaloids, etc. have been scientifically reported from 9 species of *Alternanthera.* The chemical constituents (along with their structure) isolated from different species of the *Alternanthera* genus are shown in Table 1.

**FIGURE 2:**
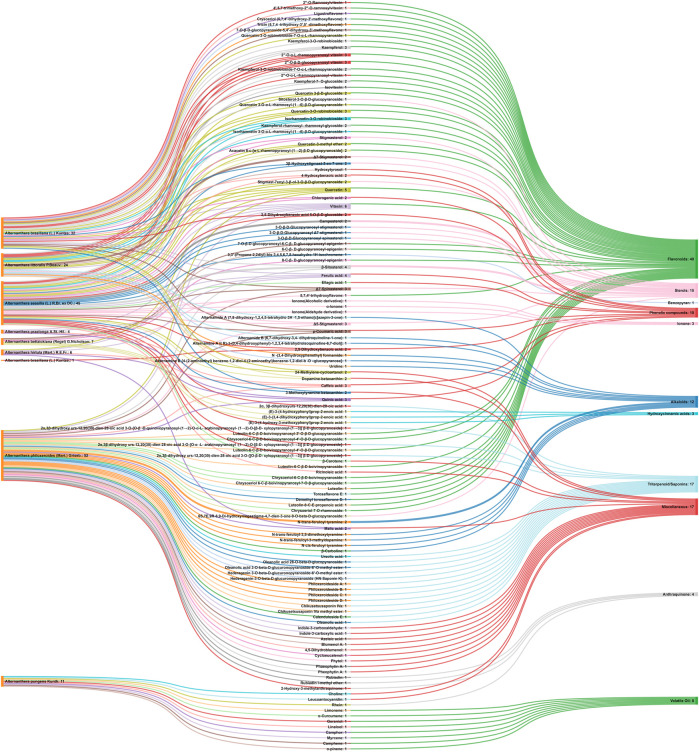
Interaction analysis map to express association and relationship between phytochemical classifications, compounds, and biological sources.

Referring to the data tabulated in Table 1 covering the isolated phytoconstituents from 9 species of Alternanthera genus, we have prepared an interactive mapping (Figure 2) to give some quick insight about it to the readers. Notably, it has also been observed that some of the phytocompounds like kaempferol, stigmasterol, quercetin, vitexin, ferulic acid, caffeic acid, etc have been isolated from various species of Alternanthera genus. This somehow lead us to suggest that these phytocompounds could serve as standardization of these markers could be helpful in identifying Alternanthera species, and avoid adulteration. Some of the compounds isolated from the species of Alternanthera genus are very common and usually been reported from multiple biological sources and well known for many pharmacological activities. For instance, kaempferol has been isolated from various other sources including *Euonymus alatus* (Thunb.) Siebold (Fang et al., 2008; Singla et al., 2021), *Vachellia nilotica* (L.) P.J.H.Hurter and Mabb.(Singh et al., 2008), etc, with multiple therapeutic potential, including but not limited to antiproliferative (Park et al., 2021), antiviral (Arabyan et al., 2021), hepatoprotective (Alshehri et al., 2021), antioxidant (Sharma et al., 2021), etc. Similarly, chlorogenic acid had been reported from multiple resources, including *Cocos nucifera* L. (Bankar et al., 2011), apple fruit (Hulme, 1953), *Neolamarckia cadamba* (Roxb.) Bosser (Kapil et al., 1995), etc with multiple therapeutic potential like neuroprotective (Hung et al., 2021), antihepatotoxic (Kapil et al., 1995), etc. Since species of Alternanthera genus containing other compounds also along with these common phytomolecules, there could be a possibility of synergistic potential and enhanced activity. Thus, we suggest the researchers to explore the therapeutic potential based on the common bioactive compounds.

### Pharmacological Activities

Several scientific investigations were conducted to validate traditional claims of various species of *Alternanthera.* Uncharacterized/non-standardized crude extracts of various species of *Alternanthera* were used in most of these scientific pharmacological studies. *Alternanthera* species have been observed to display analgesic, anticancer, anti-inflammatory, antimicrobial, antioxidant, hepatoprotective, hypotensive, allelopathic, α-glucosidase inhibitory, anthelmintic, anti-allergic, antianxiety, sedative, antiapoptotic, antiarthritic, antiasthmatic, anticataract, anticonvulsant, antidepressant, antidiabetic, antidiarrhoeal, antifungal, antibacterial, anti-HBV, antiparkinsonian, antiprotozoal, antispasmodic, antiviral, gastrointestinal protective, immunomodulatory and wound healing activities. The plant species, extract/fraction/isolate, dose tested/route of administration, bioactive dose, positive control, negative control, *In vivo*/*in vitro* models, and mechanism of action have been summarized in Table 2.

**TABLE 2:** Pharmacological activities of genus *Alternanthera.*

S. No	Pharmacological activity	Species	Extract/fraction/isolate	Dose tested/route of administration	Bioactive dose (mg/kg, IC50, etc)	Positive control	Negative control	Animals	Experimental model (*In vivo*/*in vitro*)	Mechanism of action	References
1	Analgesic	*Alternanthera brasiliana* (L.) Kuntze	Aqueous extract of aerial parts	25, 50, 100, 200 and 400 mg/kg, *p.o*	400 mg/kg, *p.o*	Dipyrone (100 mg/kg, *p.o.*)	Distilled water (10 ml/kg, *p.o.*)	Male Swiss mice	*In vivo* - Abdominal contractions induced by acetic acid	Inhibition of the synthesis of prostaglandins and avoid the sensitization of receptors	Pelisoli Formagio et al. (2012)
			Ethanolic extract of leaves	25, 50 and 100 mg/kg, *p.o*	50 and 100 mg/kg, *p.o*	Indomethacin (10 mg/kg, *p.o.*)	0.9% saline solution	Mice of the Mus musculus strain of the Swiss line	*In vivo*—Formalin test	Suppression of proinflammatory cytokine expression and inhibition of NFκB pathway and the mitogen-activated protein kinase pathway	Coutinho et al. (2017)
		*Alternanthera littoralis* P.Beauv	Ethanolic extract of aerial parts and 2″-O-α-L-rhamnopyranosylvitexin	100, 300, 500 mg/kg, *p.o.* and 1, 10, 20, 50 mg/kg, *p.o.*; 0.3–300 μg/paw local	100, 300 mg/kg, *p.o.* and 50 mg/kg, *p.o.*; 0.3–300 μg/paw local	Dexamethasone (1 mg/kg, *s.c.*)	0.9% saline solution	Adult male and female Swiss mice	*In vivo*—carrageenan, TNF, or L-DOPA-induced hyperalgesia model	Act via prevented a Cg-induced decrease in the threshold of mechanical sensitivity	de Santana Aquino et al. (2015)
		*Alternanthera philoxeroides* (Mart.) Griseb	Methanolic extract of the whole plant	50, 100, 200 and 400 mg/kg, *p.o*	200 and 400 mg/kg, *p.o*	Aspirin (200 and 400 mg/kg, *p.o.*)	Acetic acid (1%, 10 ml/kg, *i.p.*) and 1% Tween 80 in water, (10 ml/kg)	Swiss albino mice	*In vivo*—Acetic acid-induced constriction method	Act via inhibition of prostaglandin synthesis, cyclooxygenases, and lipo-oxygenases expression	Khatun et al. (2012)
		*Alternanthera sessilis* (L.) R.Br. ex DC.	Ethanolic extract of leaves	250 and 500 mg/kg, *p.o*	500 mg/kg, *p.o*	Diclofenac sodium (25 mg/kg, *p.o.*) and morphine (5 mg/kg, *i.p.*)	Acetic acid (0.7%, 10 ml/kg, *i.p.*)	Young Swiss Albino mice	*In vivo*—acetic acid-induced writhing and hot-plate tests	Act via inhibition of IL-4, IL-5, and IL-13	Mondal et al. (2014)
			Hydroethanolic extract of leaves	250 and 500 mg/kg, *p.o*	250 and 500 mg/kg, *p.o*	Morphine (0.5 mg/kg, *i.p.*) and analgin (50 mg/kg, *i.p.*)	Acetic acid (3%, 0.1 ml/10 g, *i.p.*)	Swiss Albino mice	*In vivo*—Acetic acid-induced writhing and Eddy’s hot plate methods	Act via centrally modulating mechanisms involving opiate, dopaminergic descending noradrenergic, and serotonergic receptor systems or maybe by peripherally inhibiting the prostaglandins, leukotrienes, and other endogenous substances	Mohapatra et al. (2018)
			Methanolic extract of aerial parts	50, 100, 200 and 400 mg/kg, *p.o*	200 and 400 mg/kg, *p.o*	Aspirin (200 and 400 mg/kg, *p.o.*)	1% Tween 80 in water, 10 ml/kg and 1% acetic acid (10 ml/kg *i.p.*)	Swiss albino mice	*In vivo* - acetic acid-induced pain model	Act via preventing prostaglandin production through inhibition of lipooxygenase and cyclooxygenase	Hossain et al. (2014)
			Ethanolic extract of aerial parts	200 and 400 mg/kg, p.o	400 mg/kg, p.o	Diclofenac sodium (100 mg/kg, po.)	Saline (10 mg/kg, p.o.)	Female Swiss albino mice	*In vivo*: Acetic acid-induced writhing test and hot plate test	_____	Mohaimenul et al. (2020)
2	Anthelmintic	*Alternanthera sessilis* (L.) R.Br. ex DC.	Aqueous, methanolic, and acetone extracts of leaves	25, 50, 75 and 100 mg	25, 50, 75 and 100 mg	Albendazole (15 mg/ml)	10% propylene glycol in normal saline	Indian adult earthworms *Pheretima Posthuma*	*In vitro - Pheretima Posthuma* method	Act via lysis of mucopolysaccharide membrane and cause paralysis or death of the worm	Vennila and Nivetha, (2015)
					Methanol extract showed potent activity						
			Ethanolic extract of the whole plant and ellagic acid	1.56–50 mg/ml and 0.09–3 mg/ml	1.56–50 mg/ml and 0.09–3 mg/ml	Albendazole (1.5 mg/ml)	1.0% tween-80 in phosphate-buffered saline	*Haemonchus contortus* strain	*In vitro* - Adult motility test	Act via disrupting cell membrane permeability through pore formation, the disintegration of integuments at a specific site, inhibition of cAMP phosphodiesterase and Na^+^/K^+^ ATPase	Mondal et al. (2015)
3	Antiallergic	*Alternanthera sessilis* (L.) R.Br. ex DC.	95% Ethanolic extract of aerial parts	25, 50 and 100 μg/ml	25, 50 and 100 μg/ml	—	—	Rat basophilic leukemia cells	*In vitro*—Estimation of calcium, IL-6, TNF-α, IL-13, IL-4, lactate dehydrogenase release, *β*-hexosaminidase secretion assay, and Western Blot Analysis	Act via inhibition of antigen-stimulated secretion of TNF-α and IL-6 production and attenuates activation of NF-κB	Rayees et al. (2013)
4	Antianxiety	*Alternanthera brasiliana* (L.) Kuntze	Aqueous extract of leaves	100, 200 and 400 mg/kg, *p.o*	400 mg/kg, *p.o*	—	Distilled water (10 ml/kg, *p.o.*)	Male adult Wistar rats	*In vivo*—Elevated plus-maze model	---------	Pelisoli Formagio et al. (2012)
			Ethanolic extract of leaves	250, 500 and 1,000 mg/kg, *p.o*	1,000 mg/kg, *p.o*	Diazepam (1 mg/kg, i.*p.*)	Saline (10 ml/kg, p.o.)	Albino mice	*In vivo*—Hole board test and Elevated plus maze test	Benzodiazepine-like or GABA receptor-related action or 5-HT partial agonists like buspirone	Oyemitan et al. (2015)
			Methanolic extract of leaves	100, 300 and 600 mg/kg, *i.p*	100, 300 and 600 mg/kg, *i.p*	Diazepam (1 mg/kg, *i.p.*)	Distilled water (10 ml/kg, *p.o.*)	Adult male Swiss albino mice	*In vivo—*Hole board test, open field test, elevated plus maze test, light/dark exploration test, and actophotometer test	Direct activation of GABA receptors	Barua et al. (2013)
		*Alternanthera philoxeroides* (Mart.) Griseb	Ethanolic extract of leaves	250 and 500 mg/kg, *p.o*	250 and 500 mg/kg, *p.o*	17*β*-Estradiol (1 mg/kg, i.*p.*)	Distilled water (10 ml/kg, *p.o.*)	Female ICR mice	*In vivo*—Elevated plus-maze test, Light/Dark transition test, and Locomotor activity test	Act via estrogenic activity	Khamphukdee et al. (2017)
5	Antiapoptotic	*Alternanthera bettzickiana* (Regel) G.Nicholson	Ethanolic extract of the whole plant	20 and 50 μg/ml	20 and 50 μg/ml	Quercetin (10, 20 mΜ)	High glucose (25 mmol/L)	—	*In vitro*—high glucose (25 mmol/l)-induced pancreatic β-cell apoptosis and dysfunction	Act via maintaining β-cell viability; suppressing reactive oxygen species production; inhibiting the activation of caspase-9, caspase-3, cleavage of poly (ADP-ribose) polymerase; upregulating pancreatic expression and the insulin secretagogue action of pancreatic β-cells	Wu et al. (2013)
6	Antiarthritic	*Alternanthera bettzickiana* (Regel) G.Nicholson	Ethanolic extract of aerial parts	*In vivo* study: 250, 500 and 1,000 mg/kg, p.o. for 28 days	Dose dependently significantly decreased paw swelling, MDA level, improved biochemical and hematological parameters, increased SOD and CAT levels	*In vivo* study: Diclofenac sodium (10 mg/kg, p.o.)	Distilled water	Wistar rats	*In vivo:* Paw swelling, Complete Freund’s Adjuvant induced arthritis	Downregulation of nuclear factor (NF)-kB, COX-2, interleukin (IL)-6, tumor necrosis factor (TNF)-α, and IL-1β and upregulation of IL-10, I-kB, and IL-4	Manan et al. (2020)
				*In vitro* study: 50–6,400 μg/ml		*In vitro* study: Diclofenac sodium (50–6,400 μg/ml)			*In vitro*: Egg Albumin Denaturation Inhibition, Inhibition of Protein Denaturation Using Bovine Serum Albumin		
		*Alternanthera philoxeroides* (Mart.) Griseb., *Alternanthera sessilis* (L.) R.Br. ex DC.	Ethanolic extract of leaves	100–500 μg/ml	500 μg/ml	Diclofenac sodium (100–500 μg/ml)	—	—	*In vitro*—Bovine Serum protein denaturation method and Egg albumin denaturation method	Inhibits thermally-induced protein denaturation	Sunmathi et al. (2016)
7	Antiasthmatic	*Alternanthera sessilis* (L.) R.Br. ex DC.	Ethanolic extract of leaves	500 mg/kg, *p.o*	500 mg/kg, *p.o*	Mepyramine (8 mg/kg, *p.o.*)	Saline (1 ml/kg, *p.o.*)	Guinea pigs	*In vivo—*Bronchial hyperreactivity by Histamine aerosol induced bronchospasm in guinea pigs and broncho-alveolar lavage fluid (BALF) in egg albumin sensitized guinea pigs	Act via inhibition of antigen-induced histamine release or reduction in leucocyte count	Fathima et al. (2016)
			70% Ethanolic extract of the whole plant and its dichloromethane and aqueous fractions	—	1–10 mg/kg *i.p*	Verapamil (1–10 m g/kg, *i.p.*)	Acetylcholine (1 μg/kg)	Sprague-Dawley albino rats	*In vivo—*acetylcholine chloride (Ach)-induced-bronchospasm	Act via calcium channel blocking potential	Saqib and Janbaz, (2016)
8	Anticancer/Cytotoxic	*Alternanthera bettzickiana* (Regel) G.Nicholson	Aqueous extract of leaves	10–100 μM	50–100 μM	β-actin antibody	—	Lung cancer (A549) cell lines	*In vitro*—MTT assay	Act via decline in cell proliferation, disturbances in the activity of mitochondrial membrane, the process of DNA fragmentation and apoptosis in cell line	Nagalingam et al. (2018)
			Aqueous extract of leaves and silver nanoparticles and Ag-mesoporous MnO2 nanocomposite	2.5–30 μM	10, 30 μM	—	A group without extract/drug	Human HT-29 and SW620 colon cancer cell lines	*In vitro—*MTT assay	Cell death through the generation of intracellular oxidative stress	Jothi Ramalingam et al. (2017)
		*Alternanthera brasiliana* (L.) Kuntze	Aqueous fraction of the ethanolic extract from the leaves, robinin, clovin, quercetin 3-O-robinobioside, kaempferol 3-O-robinobioside, kaempferol 3-O-rutinoside-7-O-α-L-rhamnopyranoside and kaempferol 3-O-rutinoside	10–100 μg/ml	Kaempferol 3-O-robinobioside and kaempferol 3-O-rutinoside: IC_50_ = 25 μg/ml	Azathioprine	—	Human peripheral blood mononuclear cells	*In vitro—*Lymphocyte proliferation assay	Inhibition of the proliferative response of human T-cells	Brochado et al. (2003)
			Ethyl acetate extract of leaves	4, 8, 16, 32 and 64 μg/ml	IC_50_ = 33.54 and 33.69 μg	5-Fluorouracil (20 mg/kg i.p.)	Distilled water, *p.o.,* and Ehrlich ascites carcinoma cells (2 × 10^6^ cells/mouse i.p.)	Ehrlich ascites carcinoma cells	*In vitro—*Trypan blue dye exclusion method and MTT assay	Decreased the levels of lipid peroxidation and significantly increased the levels of GSH, SOD, and catalase	Samudrala et al. (2015)
				200 and 400 mg/kg, *p.o*	200 and 400 mg/kg, *p.o*			Swiss albino mice	*In vivo—*Ehrlich ascites carcinoma method		
		*Alternanthera philoxeroides* (Mart.) Griseb	Alternanthin B; N-trans-feruloyl-3,5-dimethoxytyramine; alternanthin; N-trans-feruloyl-3-methyldopamine and N-trans-feruloyl tyramine	10 and 30 μg/ml	30 μg/ml	—	—	Hela and L929 cancer cell lines	*In vitro—*MTT assay	Cytotoxic effect against Hela and L929 cancer cell lines	Fang et al. (2007)
			Philoxeroideside A-D	—	Philoxeroideside D IC_50_ = 37.29 (SK-N-SH) and 45.93 (HL60) μg/ml	—	—	SK-N-SH and HL60 cell lines	*In vitro—*3-(4,5-dimethylthiazol-2-yl)-2,5-diphenyltetrazolium bromide colorimetric assay	Cytotoxic effect against SK-N-SH and HL60 cell lines	Fang et al. (2009b)
			Methanolic extract of leaves	10, 20, 40, 80, 160 mg/ml	160 mg/ml	—	Cardiomyocyte apoptosis induced by doxorubicin	H9c2 cell lines	*In vitro—*MTT assay and annexin V-FITC/PI staining assay	Decreased the cell apoptosis induced by doxorubicin	Zhang et al. (2018)
		*Alternanthera philoxeroides* (Mart.) Griseb.; *Alternanthera hirtula* (Mart.) R.E.Fr. and *Alternanthera praelonga* A.St.-Hil	Ethanolic extract of the whole plant	0.25, 2.5, 25 and 250 μg/ml	Exhibited mild activity	Doxorubicin	DMSO	UACC-62 (melanoma); MCF-7 (mamma); 786-O (kidney); NCI-H460 (lung); PC-3 (prostate); OVCAR-3 (ovary); HT-29 (colon); K562 (leukemia). Non cancer cell line: VERO (epithelial cell from green monkey kidney)	*In vitro*—MTT assay	Toxicity against cell lines	Correa et al. (2016)
		*Alternanthera sessilis* (L.) R.Br. ex DC.	Methanolic extract of leaves	0.05–10 mg/ml	IC_50_ = 6.5 mg/ml	—	—	Vero cell lines	*In vitro—*3-(4,5-dimethylthiazol-2-yl)-2,5-diphenyltetrazolium bromide assay method	—	Jain et al. (2016)
			Silver nanoparticles of the aqueous extract	1.56, 3.12, 6.25, 12.5, 25 μg/ml	IC_50_ = 6.85 μg/ml	—	Normal saline/DMSO	PC3 human prostate cancer cell line	*In vitro—*MTT assay	Apoptosis dependent pathway	Firdhouse and Lalitha, (2013)
			Gold nanoparticles of the aqueous extract of leaves	1–15 mg/ml	10–15 mg/ml	—	A group without extract/drug	HeLa cervical cancer cell lines	*In vitro—*MTT assay	Act via modulating intrinsic apoptotic mechanisms in cervical cancer cells	Qian et al. (2019)
			Aqueous extract of leaves and stems	20–100 μg/ml	20–100 μg/ml	—	A group without extract/drug	SIRC rabbit corneal cell line	*In vitro—*MTT assay	Act via inhibiting cytotoxic nature of the pathogen causing ocular diseases	Suganya et al. (2019)
			n-hexane and methanolic extracts of aerial parts	7.81, 15.625, 31.25, 62.5, 125 and 250 μg/ml	LC_50_values of methanol and n-hexane extracts are 255.4 and 925.68 μg/ml respectively	—	DMSO (2.5 ml)	—	*In vitro* Brine Shrimp lethality assay	—	Pathak et al. (2020)
			Ethanolic extract of aerial parts	800, 400, 200 and 100 μg/ml	LC_50_–1,364 μg/ml	Vincristine sulphate (LC_50_–0.93 μg/ml)	DMSO (1%)	—	*In vitro* Brine Shrimp lethality assay	—	Mohaimenul et al. (2020)
			Ethanolic, 70% ethanolic, 80% methanolic, ethyl acetate, and aqueous extracts of the whole plant	5–40 μg/ml	Ethanol and water extracts exhibited potent activity in a concentration-dependent manner	fenofibrate (0.1 mM)	A group without extract/drug	HepG2, a human hepatic cancer cell line	*In vitro*—preventive and ameliorative effects against palmitate-induced lipid accumulation in HepG2	Act via preventing steatosis (intracellular lipid content reduced)	Yap et al. (2019)
		*Alternanthera sessilis* (L.) R.Br. ex DC.	Silver nanoparticles of the aqueous extract of leaves	25 and 50 μg/ml	IC_50_ = 42.5 μg/ml	Quercetin	Group without extract/drug	Human breast adenocarcinoma (MCF-7) cell line	*In vitro* - MTT assay	Act via decreasing expression of MMP- 9 in the cancer cells and inhibit cancer cell migration and reduce the chances of metastasis in human breast cancer	Sathishkumar et al. (2016)
			Ethanolic extract of aerial parts, stem, and leaves	25–500 μg/ml	25–500 μg/ml	Paclitaxel (50 ng/ml)	A group without extract/drug	HT-29 and 3T3 human colon cancer cell line	*In vitro*—MTT assay and colony formation assay	Act via damage of plasma membrane causing necrosis of cancer cell	Arulselvan et al. (2018)
			Aqueous extract of aerial parts	5 and 50 mg/kg, *i.p*	50 mg/kg, *i.p*	—	Normal saline	Male albino Swiss mice	*In vivo—*Ehrlich ascites carcinoma model	Act via potentially reducing the number of tumor cells	Guerra et al. (2003)
9	Anticataract	*Alternanthera sessilis* (L.) R.Br. ex DC.	Ethyl acetate extract of leaves	100, 200, and 400 mg	100, 200, and 400 mg	Malondialdehyde and Inorganic Phosphorus	Cataract induced lenses	Lenses tissue	*In vitro*—lipid peroxidation and Na^+^ - K^+^ ATPase assays	Significant increase in the activity of Na^+^ - K^+^ ATPase in the lens tissue	Kota et al. (2017)
10	Anticonvulsant	*Alternanthera brasiliana* (L.) Kuntze	Ethanolic extract of leaves	250, 500 and 1,000 mg/kg, *p.o*	Mild activity at a higher dose	Diazepam (1 mg/kg, i.*p.*)	Pentylenetetrazole (85 mg/kg, *i.p.*); Strychnine (2 mg/kg, *i.p.*)	Albino mice	*In vivo—*Pentylenetetrazole (PTZ)-induced convulsions, Strychnine-induced convulsions, and Maximal electroshock seizures	Act via inhibition of blocking GABA– BZD receptor-mediated neurotransmission, regulation or stimulation of glycine in the spinal cord and blockade the entry of Ca^2+^, Na^+^ into the cells	Oyemitan et al. (2015)
						Phenytoin sodium (25 mg/kg, *i.p.*)					
			Ethanolic extract of leaves	20, 100 and 500 mg/kg, *i*.*p*	20 mg/kg, *i*.*p*	—	Distilled water (10—ml/kg, *p.o.*) and PTZ (60 mg/kg, *i.p.*)	Wistar rats	*In vivo—*Pentylenetetrazole-induced seizures in rats test	Act via activation of GABA-ergic system	Schallenberger et al. (2017)
			Methanolic extract of leaves	100, 300 and 600 mg/kg, *i.p*	100, 300 and 600 mg/kg, *i.p*	Diazepam (1 mg/kg, *i.p.*)	Distilled water (10—ml/kg, *p.o.*) and PTZ (80 mg/kg, *i.p.*)	Adult male Swiss albino mice	*In vivo—*Maximal electroshock-induced seizures and pentylenetetrazole induced seizures	Act via enhancing GABA mediated inhibition in the brain	Barua et al. (2013)
11	Antidepressant	*Alternanthera philoxeroides* (Mart.) Griseb	Ethanolic extract of leaves	250 and 500 mg/kg/day	250 and 500 mg/kg, *p.o*	17*β*-Estradiol (1 μg/kg, i.*p.*)	Distilled water (0.2—ml/mice, *p.o.*)	Female ICR mice	*In vivo—*forced swimming and tail suspension tests	Act via estrogenic activity	Khamphukdee et al. (2018)
		*Alternanthera sessilis* (L.) R.Br. ex DC.	Methanolic extract of leaves	100 and 200 mg/kg, *p.o*	100 and 200 mg/kg	Diazepam (2 mg/kg	Distilled water	Adult Swiss albino Wistar mice	*In vivo—*Tail suspension test and Forced swim test	Act via interaction with adrenergic, dopaminergic serotonergic, and GABAnergic system	Gupta and Singh, (2014)
12	Antidiabetic	*Alternanthera brasiliana* (L.) Kuntze	80% Ethanolic extract of stem and leaves	200 and 400 mg/kg, *p.o*	200 and 400 mg/kg, *p.o*	Metformin (600 μg/kg, *i.p.*)	Distilled water (1 ml, *p.o.*)	Male Swiss albino mice	*In vivo—*alloxan-induced diabetes model	Significantly decreased the elevated levels of blood glucose, lipid peroxidation, and various free radicals in experimental animals	Reza et al. (2019)
		*Alternanthera philoxeroides* (Mart.) Griseb	Methanolic extract of whole plant	50, 100, 200 and 400 mg/kg, *p.o*	200 and 400 mg/kg, *p.o*	Glibenclamide (10 mg/kg, *p.o.*)	1% Tween-80 in water, 10 ml/kg	Swiss albino mice	*In vivo—*oral glucose tolerance test	Act via regeneration of β-cells of the pancreas and inhibiting glucose absorption from the gut	Khatun et al. (2012)
			Methanol-soluble fraction	20, 40 and 60 μg/ml	60 μg/ml	—	—		*In vitro—*α-glucosidase inhibitory test	Act via inhibition of α-glucosidase enzyme	Bhattacherjee et al. (2014)
		*Alternanthera pungens* Kunth	Aqueous and ethanolic extracts of the whole plant	200 and 400 mg/kg, p.o	Dose-dependent activity	Metformin (150 mg/kg, p.o.)	Distilled water	Wistar rats	*In vivo*: Alloxan-induced hyperglycemia	—	Mourya et al. (2020)
		*Alternanthera sessilis* (L.) R.Br. ex DC.	Aqueous and ethanolic extracts of aerial parts	125, 250 and 500 mg/kg, *p.o*	125, 250 and 500 mg/kg, *p.o*	Glibenclamide (0.6 mg/kg, *p.o.*)	1% Acacia solution, *p.o.* and alloxan monohydrate (110 mg/kg, *p.o.*)	Male Wistar albino rats	*In vivo—*Alloxan induced diabetes model	Act via potentiating the existing *β*-cells of islets of Langerhan’s in diabetic rats	Kumar et al. (2011b)
			Hexane, ethyl acetate, and aqueous fractions of aerial parts	500 mg/kg, *p.o*	500 mg/kg, *p.o.* of ethyl acetate fraction	Glibenclamide (10 mg/kg, *p.o.*)	1% CMC (2 ml/kg) and streptozotocin monohydrate (40 mg/kg, *i.p.*) and pioglitazone (30 mg/kg, *i.p.*)	Male Sprague-Dawley rats	*In vivo—*Streptozotocin-induced diabetic rat test	Act via improvements in peripheral insulin sensitivity which reduces blood glucose concentration	Tan and Kim, (2013)
			Methanolic extract of aerial parts	50, 100, 200 and 400 mg/kg, *p.o*	200 and 400 mg/kg, *p.o*	Glibenclamide ide (10 mg/kg, *p.o.*)	1% Tween 80 in water, 10 ml/kg, and Glucose (2 g/kg, *p.o.*)	Swiss albino mice	*In vivo—*oral glucose tolerance tests	Act via potentiating pancreatic insulin secretion or by increasing glucose uptake	Hossain et al. (2014)
			Petroleum ether extract of leaves	25–100 μg/ml	25 μg/ml	Acarbose (25–100 μg/ml)	—	—	*In vitro* - α-amylase inhibition assay	—	Sundar et al. (2019)
			95% Ethanolic extract of the whole plant	200 and 400 mg/kg, *p.o*	200 and 400 mg/kg, *p.o*	Glibenclamide (10 mg/kg, *p.o.*)	Saline (2 ml/kg, *p.o.*) and streptozotocin monohydrate (50 mg/kg, *i.p.*)	Wistar albino rats	*In vivo -* Streptozotocin-induced diabetes	Act via protective action on lipid peroxidation, enhancing effects on cellular antioxidant defense and protection against oxidative damage	Das et al. (2015)
			Ethanolic extract of the whole plant	200 mg/kg, *p.o*	200 mg/kg, *p.o*	Glibenclamide (90 μg/kg, *p.o.*)	Tween 20 (0.2 ml, *p.o.*) and streptozotocin monohydrate (50 mg/kg, *i.p.*)	Male Albino Wistar rats	*In vivo -* Streptozotocin-induced diabetes	Reduction in blood glucose levels	Rao et al. (2011)
			n-hexane, ethyl acetate, and water fractions of the Methanolic extract of leaves	Up to 20 mg/ml	Ethyl acetate fraction IC_50_ α-amylase—0.52 mg/ml IC_50_ α-glucosidase—2.82 mg/ml	Acarbose IC_50_ α-amylase—0.0025 mg/ml IC_50_ α-glucosidase—0.36 mg/ml	—	—	*In vitro* α-amylase and α-glucosidase inhibitory activities	—	Manalo et al. (2020)
			Ethanolic extract of aerial parts	200 mg/kg, p.o	200 mg/kg, p.o	Metformin (150 mg/kg, p.o.)	Saline (10 mg/kg, p.o.)	Female Swiss albino mice	*In vivo*: Alloxan-induced hyperglycemia	—	Mohaimenul et al. (2020)
			Juice	20 and 100 μl	100 μl	—	—	Male adult Wistar rats	*In vitro*—pancreatic α-amylase inhibition assay and rat intestinal α-glucosidase inhibition assay	Act via lysis of cell membrane and inhibiting protein synthesis	Tiwari et al. (2013)
			Hexane, chloroform, ethyl acetate, butanol, and aqueous fractions of methanolic extracts of leaves and callus	—	Leaf ethyl acetate fraction and Callus ethyl acetate fraction exhibited potent anti-glucosidase	Acarbose	—	—	*In vitro*—α-glucosidase inhibitory test	Act via inhibition of α-glucosidase enzyme	Chai et al. (2016)
13	Antidiarrhoeal	*Alternanthera sessilis* (L.) R.Br. ex DC.	Hexane, chloroform, methanolic, and aqueous extracts of the whole plant	50 and 100 mg/kg, *p.o.* of each extract	Methanol and aqueous extracts exhibit potent activity	Diphenoxylate (2.5 mg/kg)	Polyvinylpyrrolidone, Castor oil (0.1 ml/mice; 1 ml/rats) or MgSO_4_ (2 g/kg)	Male Wistar rats and CD1 strain male mice	*In vivo*—Diarrhoea induced by castor oil and MgSO4	Inhibition of water and electrolyte transport through the intestinal mucosa or enhancing peristalsis in the intestine	Zavala et al. (1998)
14	Antigout	*Alternanthera sessilis* (L.) R.Br. ex DC.	Methanolic extract of aerial parts	100–1,000 μg/ml	IC_50_–557.77 μg/ml	Allopurinol (IC_50_–6.1 μg/ml)	DMSO	—	*In vitro:* Xanthine oxidase inhibitory assay	Xanthine oxidase inhibition	Chong and Loh, (2020)
15	Anti-HBV	*Alternanthera philoxeroides* (Mart.) Griseb	luteolin-6-C-*β*-D-boivinopyranosyl-3′-O- *β*-D-glucopyranoside; chrysoeriol-6-C- *β* D-boivinopyranosyl-4′-O- *β* D-glucopyranoside; luteolin-6-C-*β*-D-boivinopyranosyl-4′-O- *β*-D-glucopyranoside; luteolin-6-C-*β*-D-boivinopyranoside and chrysoeriol-6-C- *β*-D-boivinopyranoside	—	—	—	DMEM with 0.2% DMSO	HepG2.2.15 cells	*In vitro*—Inhibition of HBsAg and HBeAg secretions HepG and MTT assay	Act via inhibiting the secretion of HBsAg in HepG2.2.15	Li et al. (2016)
16	Antihypertensive	*Alternanthera sessilis* (L.) R.Br. ex DC.	70% Ethanolic extract of the whole plant and its dichloromethane and aqueous fractions	1–10 mg/kg, *i.p*	Ethanol extract: 1–10 mg/kg, *i.p*	Verapamil (1–10 m g/kg, *i.p.*)	Adrenaline (1 μg/kg)	Sprague-Dawley albino rats	*In vivo*—ketamine (50–80 mg/kg, *i.p.*) –diazepam (5 mg/kg, *i.p.*) anaesthetized normotensive rats	Decreased both systolic and diastolic blood pressure of the anesthetized rats	Saqib and Janbaz, (2016)
17	Anti-inflammatory	*Alternanthera brasiliana* (L.) Kuntze	Aqueous extract of leaves	200 or 400 mg/kg, *p.o*	400 mg/kg, *p.o*	Indomethacin (10 mg/kg, *p.o.*)	Distilled water (10 ml/kg, *p.o.*)	Male adult Wistar rats	*In vivo*—carrageenan-induced pleurisy	Reduction of polymorphonuclear cells and the increase of mononuclear cells in the exudate of animals	Pelisoli Formagio et al. (2012)
			Methanolic extract of leaves	300, 600 and 900 mg/kg, *p.o*	600 mg/kg, *p.o*	Sulfasalazine (360 mg/kg, *p.o.*)	Normal saline and 4% acetic acid (1 ml, *t.r.*)	Adult Wistar albino rats	*In vivo*—acetic acid-induced colitis model of inflammatory bowel disease	Significantly reduced colon weight and decreased macroscopic and microscopic score	P et al. (2016)
		*Alternanthera littoralis* P.Beauv	Ethanolic extract of aerial parts 2″-O-α-L-rhamnopyranosylvitexin	30, 100, 300 mg/kg, *p.o.* 1, 10, 20 mg/kg, *p.o*	100, 300 mg/kg, *p.o.* 1, 10, 20 mg/kg, *p.o*	Dexamethasone (1 mg/kg, *s.c.*)	0.9% saline solution	Adult male and female Swiss mice	*In vivo*—carrageenan-induced paw edema and carrageenan-induced pleurisy method	Act via inhibiting TrpV1, oxidative stress, cytokines	de Santana Aquino et al. (2015)
		*Alternanthera philoxeroides* (Mart.) Griseb., *Alternanthera sessilis* (L.) R.Br. ex DC.	Ethanolic extract of leaves	100–500 μg/ml	500 μg/ml	Diclofenac sodium (100–500 μg/ml)	—	—	*In vitro*—% Membrane stabilization and % Haemolysis	Act via by inhibiting hypotonicity induced lysis of erythrocyte membrane and inhibition of the release of phospholipases	Sunmathi et al. (2016)
		*Alternanthera pungens* Kunth	Aqueous extract of leaves	200 mg kg, *i.p*	200 mg kg, *i.p*	Indomethacin (10 mg/kg, *i.p.*)	1% Carrageenan (0.1 ml, *i.p.*)	Wistar strain rats	*In vivo*—carrageenan-induced inflammatory test	Decreased level of release of histamine serotonin and kinin, prostaglandin, proteases, lysosomes, and protein C-reactive	Franck et al. (2016)
		*Alternanthera sessilis* (L.) R.Br. ex DC.	90% ethanolic extract of stems	25, 50, 100, 200, 300, 400 and 500 μg/ml	200 or 500 μg/ml	Dexamethasone (0.5 μg/ml)	Untreated cells	RAW 264.7 murine macrophage cell line	*In vitro*—cell viability assay, quantifying the nitric oxide, proinflammatory cytokine production, nuclear translocation of NF-κB p65, and protein expression analysis	Reduced the level of proinflammatory cytokines and mediators in LPS- stimulated RAW 264.7 macrophages by inactivating their corresponding genes at the transcriptional level and by preventing the activation of the NF- κB pathway	Muniandy et al. (2018a)
			Petroleum ether and methanolic extracts of leaves	100, 200 and 300 μg/ml	Methanol extract (100 μg/ml)	Aspirin (100, 200 and 300 μg/ml)	Group without extract/drug	—	*In vitro*—protein denaturation method	—	Sundar et al. (2019)
		*Alternanthera sessilis* (L.) R.Br. ex DC.	Ethanolic extract of whole plant (EEAT) 2″-O-β-D-glucopyranosyl-vitexin	30, 100 and 300 mg/kg, p.o. 0.1, 1 and 10 mg/kg, p.o	100 mg/kg, p.o. 1 mg/kg, p.o	Prednisolone (3 mg/kg, p.o.)		Swiss mice	*In vivo*: Carrageenan-induced paw edema, zymosan-articular inflammation, carrageenan pleurisy, and complete Freund’s adjuvant	Significantly inhibited (i) edema, mechanical hyperalgesia in carrageenan-induced paw inflammation; (ii) leukocyte migration and protein extravasation in carrageenan-induced pleurisy; (iii) knee edema, mechanical hyperalgesia, and leukocyte migration in articular inflammation induced by zymosan	Kassuya et al. (2021)
			Aqueous extract of the whole plant	200 and 400 mg/kg, *i.p*	200 and 400 mg/kg, *i.p*	Indomethacin (5 mg/kg, *i.p.*)	Sterile saline (0.2 ml, *i.p.*)	Male BALB/c mice	*In vivo*—Carrageenan-induced edema method	Cyclooxygenase -1 and -2 inhibition	Biella et al. (2008)
18	Antimicrobial	*Alternanthera bettzickiana* (Regel) G.Nicholson	Hexane, chloroform, ethyl acetate, methanolic, and aqueous extracts of leaves	125, 250, 500 and 1,000 μg/ml	Mild activity	Cotrimoxazole (23.75µg/disc), Ciproflaxocin (5µg/disc), Chloramphenicol (30µg/disc) and Piperacillin (100µg/disc)	Sterile distilled water	Various bacterial strains	*In vitro* - Kirby-Bauer disc diffusion method	Act via lysis of bacterial cell wall and inhibiting protein synthesis	Vidhya et al. (2015)
			Aqueous extract of leaves and silver nanoparticles and Ag-mesoporous MnO2 nanocomposite	5–100 μg/ml	100 μg/ml of Silver nanoparticles and Ag-mesoporous MnO2 nanocomposite	—	DMSO	Various bacterial strains	*In vitro*—Agar well diffusion assay	Act via inhibition of DNA replication and blocking cellular respiration	Jothi Ramalingam et al. (2017)
			Aqueous extract of leaves (Au-NP)	10, 20, 30 or 40 μl	10, 20, 30 or 40 μl	Ciprofloxacin	—	Various bacterial strains	*In vitro* - Agar well diffusion method	Act via inhibiting DNA gyrase, topoisomerase II, topoisomerase IV	Nagalingam et al. (2018)
		*Alternanthera brasiliana* (L.) Kuntze	Ethanolic extract of leaves	MIC = ≥1,024 μg/ml	MIC = ≥1,024 μg/ml	Gentamicin (1,024 μg/ml)	—	Various bacterial strains	*In vitro* - disk diffusion method	Act via methylation of the aminoglycoside-binding site and targeted mutations in the 30S ribosomal subunit	Coutinho et al. (2017)
			Ethanolic extract of aerial parts	7.8–1,000 μg/ml	Inactive	Amphotericin-B	DMSO	Various murine macrophages and fungal strains	*In vitro* - broth microdilution method	—	Johann et al. (2010)
		*Alternanthera caracasana* Kunth	Hexane, chloroform, methanolic, acetone, and ethyl acetate extract of aerial parts and 7-methoxycoumarin	—	Acetone and ethyl acetate extracts and 7-methoxycoumarin	Kanamycin and chloramphenicol (25 μg)	DMSO	Various bacterial strains	*In vitro* - disk diffusion method	Act via lysis of microbial cell wall and inhibiting protein synthesis	Canales-Martínez et al. (2008)
		*Alternanthera brasiliana* (L.) Kuntze	Ethanolic extract of leaves	250 mg	Mild activity	Ciprofloxacin	—	Various bacterial strains	*In vitro* - disk diffusion method	Act via lysis of microbial cell wall and inhibit protein synthesis	Akachukwu and Uchegbu, (2016)
			Silver nanoparticles from aqueous extract of leaves	20–100 μg/ml	20–100 μg/ml	—	DMSO	Various bacterial strains	*In vitro*—Agar well diffusion assay	Act via inhibition of DNA replication and blocking cellular respiration	Kumar et al. (2014)
		*Alternanthera littoralis* P.Beauv	Hexane and ethanolic extract of leaves	25 mg/ml (Final reactive concentration: 2,625 µg/105 µl)	25 mg/ml(Final reactive concentration: 2,625 µg/105 µl)	ketoconazole (0.20 mg/ml) and methylene blue (0.05 mg/ml)	Propylene glycol/distilled sterilized water (5:95)	Various fungal strains	*In vitro* - agar-well diffusion method	Microbial membrane lysis and protein degradation	Gasparetto et al. (2010)
		*Alternanthera nodiflora* R.Br	Aqueous and methanolic extracts of the whole plant	25–100 mg/ml	Methanol extract (100 mg/ml)	—	—	Various bacterial and fungal strains	*In vitro* - agar well diffusion method	Act via lysis of microbial cell wall and inhibiting protein synthesis	Feka et al. (2014)
		*Alternanthera philoxeroides* (Mart.) Griseb	Methanol-soluble fraction of leaves	20, 40 and 60 μg/ml	60 μg/ml	—	—	Various bacterial strains	*In vitro* - disc diffusion assay	Act via lysis of bacterial cell wall and inhibit protein synthesis	Bhattacherjee et al. (2014)
			Aqueous and chloroform: methanol (1:1) extracts of leaves	35.25–80 μg/ml	35.25–80 μg/ml	—	Distilled water and DMSO	Various bacterial strains	*In vitro*—disc diffusion method	—	Rawani et al. (2011)
			Ethanolic extract of leaves	500, 750 and 1,000 μg/ml	1,000 μg/ml	Tetracycline (30 μg/ml) for bacteria and fluconazole (100 μg/ml) for fungi	DMSO	Various bacterial and fungal strains	*In vitro*—Agar well diffusion assay	Act via lysis of microbial cell wall and inhibit protein synthesis	Pulipati et al. (2016)
			Methanolic extract of leaves, stem and roots n-hexane, chloroform and ethyl acetate fractions	100 mg/ml	100 mg/ml	Penicillin (100 mg/ml)	DMSO (166 µl)	Bacterial phytopathogens (*Erwinia carotovora*, *Ralstonia solanacearum,* and *Xanthomonas axonopodis*)	*In vitro*; Disk diffusion method n-hexane fraction maximum zone of inhibition	—	Akbar et al. (2021)
			Methanolic extract of leaves	500, 750 and 1,000 μg/ml	1,000 μg/ml	Nitrofurantoin (300 µg/disc)	DMSO	Multidrug-resistant uropathogens (*Staphylococcus aureus, Staphylococcus saprophyticus, Enterococcus faecalis, Escherichia coli, Klebsiella pneumoniae, Pseudomonas aeruginosa, Proteus vulgaris,* and *Proteus mirabilis*)	*In vitro*; Agar well diffusion method Inhibition rate observed in the following order: *S. saprophyticus* > *S. aureus* > *K. pneumoniae E. coli*, *P. vulgaris* > *E. faecalis, P. aeruginosa* > *P. Mirabilis*	—	Pulipati and Babu, (2020)
		*Alternanthera philoxeroides* (Mart.) Griseb. and *Alternanthera sessilis* (L.) R.Br. ex DC.	Aqueous extract of leaves	—	Both plants exhibited antibacterial only	—	—	Various bacterial and fungal strains	*In vitro* - agar well diffusion method	Act via lysis of microbial cell wall and inhibit protein synthesis	Kumari and Krishnan, (2016)
			Ethanolic extract of leaves	10, 25, and 50 μg	10, 25, and 50 μg	Gentamycin/Nystatin	Ethanol	Various bacterial and fungal strains	*In vitro* - Well diffusion assay	—	Sivakumar and Sunmathi, (2016)
		*Alternanthera pungens* Kunth	Aqueous, acetone, ethanolic, and petroleum ether extracts of aerial parts	25–200 mg/ml	All extracts exhibited antibacterial potential but the antifungal profile was shown by only acetone and aqueous extracts	Ampicillin (100 μg/ml) and Miconazole (100 μg/ml)	DMSO	Various bacterial and fungal strains	*In vitro*—Agar well diffusion assay	Act via inhibition of DNA replication and blocking cellular respiration	Jakhar and Dahiya, (2017)
		*Alternanthera sessilis* (L.) R.Br. ex DC.	Hexane and methanolic extracts of aerial parts	2–16 mg/ml	Mild action	Cefotaxime (2–16 μg/ml)	—	Various bacterial strains	*In vitro*—agar dilution method	Act via lysis of microbial cell wall and inhibiting protein synthesis	Osuna et al. (2008)
			Petroleum ether (40–60°C), chloroform, acetone, methanolic, and aqueous extracts of leaves	5–75 µg	Chloroform extract exhibited a potent antibacterial profile	Ciprofloxacin (5–75 µg) and fluconazole (5–75 µg)	—	Various bacterial and fungal strains	*In vitro* - cup plate and turbidimetric methods	—	Jalalpure et al. (2008)
			Aqueous, ethanolic, and acetone extracts of leaves	1,000 μg/ml	25.7 and 252.5 μg/ml	Tetracycline and ketoconazole	—	Various bacterial and fungal strains	*In vitro* - Kirby-Bauer method	Act via lysis of microbial cell wall and inhibit protein synthesis	Monroy and Limsiaco, (2016)
			Silver nanoparticles of aqueous extract of leaves	100 μg/ml	100 μg/ml	---------	—	Various bacterial strains	*In vitro* - Well diffusion assay	---------	Niraimathi et al. (2013)
			Ethanolic extract of leaves	25, 100, 250 and 500 μg/ml	500 μg/ml	—	—	Various bacterial strains	*In vitro* - agar-well diffusion method	Act via inhibition of extracellular microbial enzymes, proteins, deprivation of iron as substances for microbial growth or destroy its membranes	Rajamurugan et al. (2013)
			Aqueous extract of leaves and stems	250, 500 and 1,000 μg/μl	250, 500 and 1,000 μg/μl	—	—	Various bacterial strains	*In vitro* - agar well diffusion method	Act via lysis of microbial cell wall and inhibit protein synthesis	Suganya et al. (2019)
			Petroleum ether, ethyl acetate, chloroform, and methanolic extract of leaves	50 mg/ml	Ethyl acetate and methanol extract exhibited maximum activity	—	DMSO	Various bacterial strains	*In vitro*—Agar well diffusion assay	Act via inhibition of DNA replication and blocking cellular respiration	Kota et al. (2017)
			Petroleum ether and methanolic extracts of leaves	25, 50, and 100 µg	100 µg	Streptomycin (10 µg)	DMSO	Various bacterial strains	*In vitro*—Agar well diffusion assay	—	Sundar et al. (2019)
			Petroleum ether and methanolic extracts of leaves	10 mg	10 mg	Fluconazole (10 mg)	DMSO	Various bacterial and fungal strains	*In vitro*—Agar well diffusion assay	—	Sundar et al. (2019)
			Hexane and ethanolic extracts of the adult plants	MIC = 50–500 μg/ml	MIC = 50–500 μg/ml	Fluconazole (10 mg)	DMSO	Various bacterial and fungal strains	*In vitro*—Agar well diffusion assay	Act via destroying the cell membrane and prevent the protein synthesis	Salvador et al. (2009)
19	Antioxidant	*Alternanthera bettzickiana* (Regel) G.Nicholson	Four fractions of 80% aqueous methanolic extract of flowers	200 mg/l	8.2–67.2% Scavenging of ABTS radical; 6.9–63.8% scavenging as per FRAP assay	Rutin (10 mg/l)	Solution of stable free radicals	—	*In vitro* - ABTS, FRAP, and metal ion chelation assay	Inhibition of free radicals	Petrus et al. (2014b)
			Hexane, chloroform, ethyl acetate, methanolic, and aqueous extracts of leaves	125, 250, 500, and 1,000 μg/ml	Methanol extract exhibited strong activity IC_50_ = 293.44 μg/ml	—	Solution of stable free radicals	—	*In vitro* - DPPH radical scavenging, reducing power and total antioxidant (Ammonium molybdate) activities	Inhibition of free radicals	Vidhya et al. (2015)
		*Alternanthera brasiliana* (L.) Kuntze	80% Ethanolic extract of stem and leaves	1–1,000 μg/ml	IC_50_ = 52.02–140.05 μg/ml	Ascorbic acid	Solution of stable free radicals	—	*In vitro* - DPPH radical scavenging, reducing power, nitric oxide (NO) radical inhibition, and scavenging of hydrogen peroxide assay	Act via inhibition of free radicals	Reza et al. (2019)
		*Alternanthera brasiliana* (L.) Kuntze	Ethanolic extract of leaves	0.1–1,000 μg/ml	—	Ascorbic acid	—	—	*In vitro*—1,1-diphenyl- 2-picrylhydrazyl (DPPH) radical-scavenging, iron (II)-chelating, nitric oxide radical-scavenging, ferrous sulfate, and carbon tetrachloride-induced lipid peroxidation assays	Inhibition/inactive free radicals	Enechi et al. (2013)
			Methanolic extract of leaves	50–1,000 μg/ml	50–1,000 μg/ml	Butylated hydroxyanisole	DPPH stable free radicals	—	*In vitro*—DPPH assay	Inhibition of stable DPPH free radicals	Chandran, (2017)
			Ethanolic extract of leaves	0–1 mg/ml	Concentration-dependent activity	Vitamin C	Solution of stable free radicals	—	*In vitro*—DPPH assay, Ferric oxide reducing power assay, and Nitric oxide scavenging assay	Inhibition of stable free radicals	Attaugwu and Uvere, (2017)
			Ethanolic extract and its dichloromethane, ethyl acetate, n-butanolic fractions of leaves	Ethyl acetate fraction exhibited strong activity (IC_50_ = 163 mg/ml)	Ethyl acetate fraction exhibited strong activity (IC_50_ = 163 mg/ml)	Ascorbic acid (IC_50_ = 6.48 μg/ml)	Solution of stable free radicals	—	*In vitro*—DPPH assay	Inhibition of free radicals	Pereira et al. (2013)
			Ethanolic extract of aerial parts and its hexane, chloroform, and ethyl acetate fractions	---------	Ethanol extract and its ethyl acetate fraction exhibited maximum activity	Ascorbic acid, butylated hydroxyanisole, and butylated hydroxytoluene	Solution of stable free radicals	—	*In vitro* –DPPH and β-carotene assay	Act via inhibition of stable free radicals	Araujo et al. (2014)
		*Alternanthera brasiliana* (L.) Kuntze	Ethanolic extract of leaves	25–400 μg/ml	400 mg/ml	Ascorbic acid (25–400 μg/ml)	Solution of stable free radicals	—	*In vitro* - 2, 2-diphenyl-1-picrylhydrazyl (DPPH) and ferric reducing antioxidant power assay	Inhibition of stable free radicals	Akachukwu and Uchegbu, (2016)
		*Alternanthera ficoidea* (L.) P.Beauv	Methanolic extract of leaves, stem, and roots	IC_50_ = 442.5, 423.75 and 390.66 μg/ml, respectively, for leaves, stems and roots	IC_50_ = 442.5, 423.75 and 390.66 μg/ml, respectively, for leaves, stems and roots	Ascorbic acid	Solution of stable free radicals	—	*In vitro*—DPPH assay	Inhibition of stable free radicals	Patil and Kore, (2019)
		*Alternanthera littoralis* P.Beauv	Alternamide A-B, Alternamine A-B	—	Alternamide B (1.10 relative Trolox equivalent)	Quercetin and caffeic acid	Solution of stable free radicals	—	*In vitro*—ORAC assay	Inhibition of stable free radicals	Koolen et al. (2017)
		*Alternanthera paronychioides* A.St.-Hil	Methanolic, ethanolic, and aqueous extracts of the whole plant	Ethanolic extract	—	—	Solution of stable free radicals	—	*In vitro* - Trolox equivalent antioxidant capacity, oxygen radical absorbance capacity, and cellular antioxidant activity	Inhibition of stable free radicals	Wu et al. (2013)
			Aqueous extract of leaves	100 μg/ml	Mild activity	Trolox (0–80 nmol/μl)	—	—	*In vitro*—1,1-diphenyl- 2-picrylhydrazyl (DPPH)	Inhibition/inactive free radicals	Tukun et al. (2014)
		*Alternanthera philoxeroides* (Mart.) Griseb	Methanol-soluble fraction from leaves	20, 40 and 60 μg/ml	60 μg/ml	—	—	—	*In vitro* - DPPH and ABTS radical scavenging assay	Inhibition of stable free radicals	Bhattacherjee et al. (2014)
		*Alternanthera philoxeroides* (Mart.) Griseb.; *Alternanthera hirtula* (Mart.) R.E.Fr. and *Alternanthera praelonga* A.St.-Hil	Ethanolic extracts of the whole plant	---------	Exhibited mild activity	Quercetin, vitexin, caffeic acid, chlorogenic acid, and Trolox	Solution of stable free radicals	—	*In vitro* –DPPH assay	Inhibition of stable free radicals	Correa et al. (2016)
		*Alternanthera pungens* Kunth	Ethanolic and aqueous extracts of leaves	20–100 mg/ml	100 mg/ml	Butylated hydroxytoluene and ascorbic acid	Azinobis-3-ethybenzothiazoline-6-sulfonic acid radical and DPPH radical	—	*In vitro*—2, 2-azinobis-3-ethylbenzothiazoline-6-sulfonic acid radical scavenging assay and DPPH radical scavenging assay	Act via proton- donating ability and could serve as free radical inhibitors	Mourya et al. (2019)
			Aqueous extract of leaves	200 mg kg, *i.p*	200 mg kg, *i.p*	Vitamin C (100 mg/kg, *i.p.*)	1% Carrageenan (0.1 ml, *i.p.*)	Wistar strain rats	*In vivo*—estimation of thiobarbiturates Acid Reactive Substances assay	Act via significant reduction of serum concentration levels of TBARS	Franck et al. (2016)
			Aqueous, acetone, ethanolic, and petroleum ether extracts of aerial parts	100–1,000 μg/ml	IC_50_ = 324.43, 203.56, 100.79 and 931.63 μg/ml, respectively for extracts	Ascorbic acid (100–1,000 μg/ml)	Solution of stable free radicals	—	*In vitro*—DPPH assay	Act via inhibition of stable free radicals	Jakhar and Dahiya, (2017)
		*Alternanthera sessilis* (L.) R.Br. ex DC.	90% Methanolic, 70% acetone, 80% ethanolic extracts of leaves and stems	100–1,000 μg/ml	All extracts are active	Ascorbic acid and rutin	—	—	*In vitro -* phosphomolybdate, DPPH scavenging, superoxide scavenging, nitric oxide scavenging, and iron-chelating methods	Act via inhibition of various oxidative stress-producing species	Borah et al. (2011)
			Hexane, chloroform, ethyl acetate, butanolic, and aqueous fractions of leaves and callus methanol extracts	100 μg/ml	Ethyl acetate fraction of leaves exhibited potent antioxidant activity	Quercetin	—	—	*In vitro -* 1,1-diphenyl-2-picrylhydrazyl (DPPH) scavenging assay	Inhibition of free radicals	Chai et al. (2016)
			30% Hydroethanolic extract of the whole plant	100 μg/ml	—	Mannitol, ascorbic acid, quercetin and sodium pyruvate	Solution of respective free radicals	—	*In vitro* - scavenging of hydroxyl radicals, superoxide radical scavenging, hydrogen peroxide radical scavenging, and metal chelating tests	Inhibition of free radicals	Sharma et al. (2013)
			Separate Methanolic and hexane extracts of leaves and stems	0.05–0.20 mg/ml	Methanolic extract of leaves	Butylated hydroxytoluene	Solution of stable DPPH free radicals	—	*In vitro* - DPPH radical scavenging activity	Inhibition of DPPH free radicals	Khan et al. (2018)
			Ethanolic and aqueous extracts of aerial parts	—	The ethanolic extract exhibited strong activity	—	Solution of stable free radicals	—	*In vitro* - *β*-carotene bleaching, DPPH, ABTS, ORAC, and FRAP assay	Inhibition of free radicals	Azizah et al. (2015)
			90% hydroethanolic extract of stem	100–1,000 μg/ml	100–1,000 μg/ml	Gallic acid	DPPH stable free radicals	—	*In vitro* - DPPH assay	Inhibition of stable DPPH free radicals	Muniandy et al. (2018b)
			Ethanolic and aqueous extracts of aerial parts	100–1,000 μg/ml	—	β-carotene, ascorbic acid, Trolox, iron (II) sulfate heptahydrate	Solution of stable free radicals	—	*In vitro* - β-carotene bleaching assay, 2,2-Diphenyl-1-picrylhydrazyl (DPPH) assay, 2,2′-azinobis-3-ethylbenzothiazoline-6-sulphonic acid (ABTS) assay, Oxygen radical absorbance capacity (ORAC) assay, and Ferric reducing antioxidant power (FRAP) assay	Inhibition of stable free radicals	Othman et al. (2016)
			Juice	25, 40, and 100 μl	25, 40, and 100 μl	Ascorbic acid	Solution of stable free radicals	—	*In vitro* - 2,2-Diphenyl-1-picrylhydrazyl (DPPH) assay, 2,2′-azinobis-3-ethylbenzothiazoline-6-sulphonic acid (ABTS) assay, hydrogen peroxide (HO) scavenging assay, ferric chloride reducing assay	Inhibition of stable free radicals	Tiwari et al. (2013)
			Ethanolic extract of leaves	10, 50, 100, 250 and 500 μg/ml	IC_50_ = 364, 522 μg/ml	Ascorbic acid	Solution of stable free radicals	—	*In vitro* - DPPH radical scavenging assay, ABTS radical cation-scavenging assay, and Reducing power assay	Inhibition of stable free radicals	Rajamurugan et al. (2013)
			Methanolic extract of leaves	100–1,200 μg/ml	IC_50_ = 400 μg/ml	Ascorbic acid	Solution of stable free radicals	—	*In vitro* - DPPH radical scavenging method	Inhibition of stable free radicals	Jain et al. (2016)
			Aqueous extract of leaves and stems	10–100 μg/ml	10–100 μg/ml	Quercetin (10–100 μg/ml)	Solution of stable free radicals	—	*In vitro* - DPPH radical scavenging and Ferric reducing antioxidant power assay	Inhibition of stable free radicals	Suganya et al. (2019)
			Hexane, ethyl acetate, ethanolic, and aqueous extracts of leaves and stem	0–1,000 μg/ml	The ethanolic extract exhibited potent activity	Ascorbic acid, gallic acid, rutin, and butylated hydroxytoluene	Solution of stable free radicals	—	*In vitro*—DPPH test, Trolox equivalent antioxidant capacity, and ferric reducing antioxidant power assay	Inhibition of stable free radicals	Mohd Hazli et al. (2019)
			Silver nanoparticles from aqueous extract of leaves	100–500 μg/ml	IC_50_ = 300.6 μg/ml	Gallic acid	Solution of stable free radicals	—	*In vitro*—DPPH test	Inhibition of stable free radicals	Niraimathi et al. (2013)
			100% Ethanolic, 70% ethanolic, 80% methanolic, ethyl acetate, and aqueous extracts of the whole plant	0–1,000 μg/ml	Ethanolic extracts exhibited maximum activity (DPPH IC_50_: 82.6 μg/ml; TEAC: 0.51 mmol TE/g; FRAP: 1.95 mmol Fe^2+^/g)	Ascorbic acid, gallic acid, rutin, and butylated hydroxytoluene	Solution of stable free radicals	—	*In vitro*—DPPH test, Trolox equivalent antioxidant capacity, and ferric reducing antioxidant power assay	Inhibition of stable free radicals	Yap et al. (2019)
			Petroleum ether, ethyl acetate, chloroform, and methanolic extract of leaves	100–600 μg/ml	100–600 μg/ml	Ascorbic acid (100–600 μg/ml)	Solution of stable free radicals	—	*In vitro*—Reducing power and DPPH assay	Inhibition of stable free radicals	Kota et al. (2017)
			Petroleum ether and methanolic extracts of leaves	50, 100 and 150 μg/ml	50, 100 and 150 μg/ml	Ascorbic acid (50–150 μg/ml)	Solution of stable free radicals	—	*In vitro*—Reducing power and DPPH assay	Inhibition of stable free radicals	Sundar et al. (2019)
			n-hexane and methanolic extracts of aerial parts	10, 30, 50, 70, 90 and 110 μg/ml	Methanol extract (IC_50_–71.10 μg/ml) n-hexane extract (IC_50_–92.54 μg/ml)	Ascorbic acid (IC_50_–39.53 μg/ml)	—	—	*In vitro* DPPH assay	—	Pathak et al. (2020)
			The volatile oil of leaves and flowers	50–250 μg/ml	Flower (IC_50_ = 170 μg/ml) and leaves (IC_50_ = 179 μg/ml)	Butylated hydroxytoluene (IC_50_ = 88 μg/ml)	Solution of stable free radicals	—	*In vitro*—DPPH assay	Inhibition of stable free radicals	Khan et al. (2016)
		*Alternanthera sessilis* (L.) R.Br. ex DC.	Ethanolic extract and its four fractions; Acacetin 8-c-[*α*-L-rhamnopyranoyl-(1→2)-*β*-D-glucopyranoside]; 2″-*O*-α-L-rhamnopyranosyl-vitexin; 2″-*O*-*β*-D-glucopyranosyl vitexin and Vitexin	—	Extract, fractions, and isolates exhibited significant activity	Quercetin, isoquercitrin, caffeic acid and chlorogenic acid	Solution of stable free radicals	—	*In vitro*—ORAC assay	Inhibition of stable free radicals	Salvador et al. (2006)
20	Antiparkinsonism/Antidementia	*Alternanthera philoxeroides* (Mart.) Griseb	Ethanolic extract of the whole plant	*In vivo* study: 250 and 500 mg/kg, p.o. once daily for 8 weeks	Dose dependently improved cognitive deficits-like behavior of the estrogen-deprived mice	17β-estradiol 1 μg/kg, p.o. once daily for 8 weeks	Distilled water	OVX Female ICR mice	*In vivo:* Morris water maze task, novel object recognition task, and Y-maze task	Inhibition of lipid peroxidation in the whole brain, downregulation of neuroinflammatory cytokines (IL-1β, IL-6, and TNF-α) and upregulation of estrogen receptor-mediated facilitation genes (PI3K and AKT) in both frontal cortex and hippocampus	Khamphukdee et al. (2021)
				*In vitro* study: 100 μg/ml					*In vitro*: Amyloid aggregation inhibition and cholinesterase inhibitory activity		
		*Alternanthera sessilis* (L.) R.Br. ex DC.	Silver nanoparticles and ethanolic extract of the whole plant	20 and 200 mg/kg, p.o	20 and 200 mg/kg, p.o	Syndopa (10 mg/kg, *p.o.*)	Distilled water and Rotenone (1.5 mg/kg, *s.c.*)	Male Wistar rats	*In vivo*—rotenone model of parkinsonism	Act via the reduction in the lipid peroxidation, increase in reduced glutathione, and reduction in oxidative stress in the brain of animals	Ittiyavirah and Hameed, (2015)
21	Antiprotozoal	*Alternanthera littoralis* P.Beauv	Alternamide A-B, Alternamine A-B	—	Alternamine A (IC_50_ = 0.16 μM) and Alternamine B (IC_50_ = 0.82 μM)	Amphotericin B and crystal violet	DMSO	Various protozoal strains	*In vitro*—Trypanocidal and leishmanicidal assays	—	Koolen et al. (2017)
22	Antispasmodic	*Alternanthera sessilis* (L.) R.Br. ex DC.	Aqueous, hexane, methanolic extract, and fractions of methanol extract (F_1_-F_6_) of leaves	—	Methanolic extract and fractions of the methanolic extract (F_2_-F_4_)	—	—	Adult male Wistar rats	*In vivo*—Smooth muscle preparation, Inhibition of dose-response curves to CaCl_2_, Relaxant effect on K^+^-induced contractions, Inhibition of dose-response curves to 5-HT, and inhibition of concentration-response curve to acetylcholine (ACh)	Act via inhibition of serotonergic and Ca^2+^ influx blockade, the peristaltic movement of the rat ileum, and reduction of the intestinal transit of food in rats	Garín-Aguilar et al. (2013)
		*Alternanthera sessilis* (L.) R.Br. ex DC.	70% Ethanolic extract of the whole plant and its dichloromethane, aqueous fractions	—	Ethanolic extract: (0.01–1.0 mg/ml), aqueous fraction (0.01–0.3 mg/ml) and dichloromethane (0.01–0.1 mg/ml)	Verapamil (1–10 mg/kg, *i.p.*)	—	White albino rabbits	*In vitro*—isolated rabbit tissue preparations (i.e., jejunum, trachea, and aorta)	Decreased the contractions in terms of both frequency and magnitude	Saqib and Janbaz, (2016)
23	Antiviral	*Alternanthera philoxeroides* (Mart.) Griseb	Chikusetsusaponin IV a	—	IC_50_ = 29, 30, 73, 25, and 25	—	No drug group	HSV-1, HSV-2, human cytomegalovirus, measles virus, mumps virus, and Female BALB/c mice	*In vitro*—various viral cell	Suppressed both the intracellular virus levels and the release of the virus in a concentration-dependent manner and prevent the viral protein synthesis	Rattanathongkom et al. (2009)
									*In vivo*—mouse model of genital herpes caused by HSV-2		
24	Central-stimulating	*Alternanthera sessilis* (L.) R.Br. ex DC.	Ethanolic extract of leaves	250 and 500 mg/kg, *p.o*	500 mg/kg, *p.o*	Caffeine (20 mg/kg, *i.p.*)	Pentobarbitone (50 mg/kg, *i.p.*)	Young Swiss Albino mice	*In vivo*—Pentobarbitone induced sleeping time, open field and hole cross tests	Act via stimulating the inhibitory neurotransmitter gamma-aminobutyric acid (GABA) mediate d postsynaptic inhibition through allosteric modification of GABA-A receptors	Mondal et al. (2014)
25	Gastrointestinal protective	*Alternanthera sessilis* (L.) R.Br. ex DC.	Aqueous and ethanolic extract of the whole plant	1–300 mg/kg, *p.o*	1–300 mg/kg, *p.o*	Atropine (1 mg/kg)	—	Swiss mice	*In vitro*—charcoal meal method	Act via decreasing gastrointestinal content	Astudillo-Vázquez et al. (2008)
26	Hepatoprotective	*Alternanthera sessilis* (L.) R.Br. ex DC.	Aqueous extract of entire plant	300 mg/kg, *p.o*	300 mg/kg, *p.o*	—	Corn oil/0.9% physiological saline/polyethylene glycol 400 Carbon tetrachloride (31.25 μL/kg, *i.p.*) or acetaminophen/paracetamol (600 mg/kg, *i.p.*) in mice and D(+)-galactosamine (188 mg/kg, *i.p.*) in rats	Male ICR strain mice and male Wistar strain albino rats	*In vivo*—Carbon tetrachloride-induced hepatotoxicity	Act via inhibition of cytochrome P450, or promotion of its glucuronidation	Lin et al. (1994)
			Methanolic extract of the whole plant	50, 200 and 250 mg/kg, *p.o*	200 and 250 mg/kg, *p.o*	Silymarin (100 mg/kg, *p.o.*)	2% w/v Gum acacia suspension (1 ml/kg, *p.o.*) and carbon tetra chloride (1.25 ml/kg, *i.p.*)	Male Wistar rats	*In vivo*—carbon tetrachloride-induced hepatotoxicity	Act via significant reversal of degeneration marked by a prominent decrease of necrosis, cell integrity restoration	Bhuyan et al. (2017)
27	Immunomodulatory	*Alternanthera sessilis* (L.) R.Br. ex DC.	Aqueous extract of the whole plant	50, 100 and 200 mg/kg, *i.p*	50, 100 and 200 mg/kg, *i.p*	Sheep red blood cells (0.1 mL, 25% suspension in saline, *i.p.*)	Sterile saline (0.2 ml, *i.p.*)	Male BALB/c mice, adult guinea pigs, and adult sheep	*In vivo*—Enzyme-linked immunosorbent assay	Act via increasing production of mitogen-induced antibodies and inhibiting the production of antibodies to T-dependent antigens	Biella et al. (2008)
			Aqueous extract of aerial parts	5 and 50 mg/kg, *i.p*	50 mg/kg, *i.p*	—	Normal saline	Male albino Swiss mice	*In vivo*—mice immunized with sheep red blood cells (SRBC 10%, *i.p.*) as T-dependent antigen, or in mice stimulated with mitogens (10 μg, *Escherichia coli* lipopolysaccharide, LPS, *i.p.*)	Act via immune activation either by inhibiting or stimulating antibody production, depending on its concentration	Guerra et al. (2003)
		*Alternanthera sessilis* (L.) R.Br. ex DC.*, Alternanthera brasiliana* (L.) Kuntze and *Alternanthera littoralis* P.Beauv	Aqueous and ethanolic extract of leaves; tetrahydrofuran, dichloromethane, aqueous, petroleum ether soluble fraction	0–200 μg/ml	0–200 μg/ml	—	—	Peripheral blood mononuclear cells	*In vitro*—Natural Killer Assay	Inhibition of lymphocyte activation	Moraes et al. (1994)
										Act via activation of the cells of the immune system	
28	Insecticide	*Alternanthera brasiliana* (L.) Kuntze	Ethanolic extract of leaves	10, 20 and 40 μg/ml	10, 20 and 40 μg/ml	—	1% sucrose	Adult flies (*Drosophila melanogaster*)	*In vivo*—Toxicity against *Drosophila melanogaster* and locomotor assays	Act via inhibition of nucleic acid synthesis, DNA gyrase	Coutinho et al. (2017)
29	Lithotriptic/Antiurolithiatic	*Alternanthera sessilis* (L.) R.Br. ex DC.	Kalka - fine paste of macerated fresh plant material	0.054 g/100g, 0.108 g/100g, and 0.216 g/100 g	0.054 g/100g, 0.108 g/100g, and 0.216 g/100 g	Cystone (67.5 mg/kg)	0.75% (v/v) ethylene glycol in drinking water and coconut water (0.86 ml/200 g)	Healthy adult albino rats	*In vivo*—Ethylene glycol induced urolithiasis	Act via diuretic activity, crystallization inhibition activity, improving renal function and antioxidant activity of the drugs	Dhanya et al. (2017)
			Ethanolic extract of the whole plant	10, 20, and 40 mg	40 mg	Cystone (10 mg)	—	—	*In vitro*; Titrimetry, simultaneous flow static model, turbidimetry, and gravimetric methods	—	Babu et al. (2021)
30	Larvicidal	*Alternanthera sessilis* (L.) R.Br. ex DC.	Ethanolic extract of the whole plant	20, 40, 60, 80 and 100 μg/ml	LC_50_–66.84 μg/ml	—	—	—	Percent mortality	—	Babu et al. (2021)
31	Locomotor	*Alternanthera brasiliana* (L.) Kuntze	Aqueous extract of leaves	100, 200 and 400 mg/kg, *p.o*	400 mg/kg, *p.o*	—	Distilled water (10 ml/kg, *p.o.*)	Male adult Wistar rats	*In vivo*—Open field exposure test	Act via an increase in their exploratory activities	Pelisoli Formagio et al. (2012)
			Ethanolic extract of leaves	250, 500 and 1,000 mg/kg, *p.o*	500 and 1,000 mg/kg, *p.o*	Diazepam (1 mg/kg, i.*p.*)	Saline (10 ml/kg, p.o.)	Albino mice	*In vivo*—Novelty-induced behaviors	Act via regulation of different neurotransmitters such as GABA, ACh, noradrenaline, serotonin, glutamate, and dopamine	Oyemitan et al. (2015)
		*Alternanthera philoxeroides* (Mart.) Griseb	Ethanolic extract of leaves	250 and 500 mg/kg/day	Inactive	17*β*-Estradiol (1 μg/kg, i.*p.*)	Distilled water (0.2 ml/mice, *p.o.*)	Female ICR mice	*In vivo*—Y-maze test	—	Khamphukdee et al. (2018)
32	Nootropic	*Alternanthera sessilis* (L.) R.Br. ex DC.	Methanolic extract of leaves	100 or 200 mg/kg, *p.o*	200 mg/kg, *p.o*	*Bacopa monniera* extract (40 mg/kg, *p.o.*)	Scopolamine (0.4 mg/kg, *i.p.*)	Adult Swiss albino Wistar mice	*In vivo*—rectangular maze and Y maze tests	Act via evoking pronounced alteration behavior and better learning assessments	Gupta and Singh, (2012a)
33	Photoprotective	*Alternanthera brasiliana* (L.) Kuntze	5% w/w Gel from extract enriched with flavonoids	—	5% w/w flavonoids rich gel	—	Gel base	—	*In vitro*—Mansur method	Act via the ability to stabilize reactive oxygen species, due to the presence of hydroxyl groups attached to the aromatic rings, allowing the resonance	Alencar Filho et al. (2020)
34	Sedative	*Alternanthera brasiliana* (L.) Kuntze	Ethanolic extract of leaves	250, 500 and 1,000 mg/kg, *p.o*	250, 500 and 1,000 mg/kg, *p.o*	Diazepam (1 mg/kg, i.*p.*)	Saline (10 ml/kg, p.o.)	Albino mice	*In vivo*—ketamine-induced hypnosis test	Act via stimulatory or central excitatory effect	Oyemitan et al. (2015)
						Ketamine (100 mg/kg, *i.p.*)					
35	Wound healing	*Alternanthera brasiliana* (L.) Kuntze	Methanolic extract of leaves	5% ointment applied topically; 200 and 400 μg	5% ointment applied topically; 400 μg	Himax ointment	Vaseline ointment and methylcellulose	Sprague Dawley rats	*In vivo*—Excision and incision wound model and Chorioallantoic membrane model	Act via an increase in collagen concentration and stabilization of fibers	Barua et al. (2009)
			Methanolic extract of leaves	5% w/w ointment applied topically	5% w/w ointment applied topically	Himax ointment	Soft white petroleum jelly	Sprague Dawley rats	*In vivo*—burn wound model	Act via formation of the epidermis with keratin layer and deposition of collagen fibers	Barua et al. (2012a)
			Methanolic extract of leaves	2.5, 5.0 and 7.5% (w/w) ointment	2.5, 5.0 and 7.5% (w/w) ointment	Himax ointment	Soft white petroleum jelly	Adult Sprague Dawley rats	*In vitro*—immunocompromised wound model	Act via collagen deposition, fibroblast proliferation, angiogenesis, and development of basement membrane	Barua et al. (2012b)
			Methanolic extract of leaves	5% (w/w) ointment	5% (w/w) ointment	—	Soft white petroleum jelly	Healthy Sprague Dawley rats	*In vivo*—excision wound model	Act via wound contraction, fibroblastic deposition	Baru et al. (2012)
		*Alternanthera sessilis* (L.) R.Br. ex DC.	90% hydroethanolic extract of stem	12.5–500 μg/ml	50 and 300 μg/ml	Allantoin (50 μg/ml)	The natural rate of migration and viability of cells without extract	NHDF, HDF-D, and HaCaT cells	*In vitro*—wound scratch and MTT assay	Act via formation of the epidermis with keratin layer and deposition of collagen fibers	Muniandy et al. (2018b)
			Chloroform extract of leaves	200 μg/ml	200 μg/ml	—	Saline water	Albino rats	*In vivo*—excision wound, incision wound, and dead space wound model	Act via increase collagen content, degree of collagen cross-linkage within the wound and promotes cell division, growth of bone, cartilage, and other connective tissues	Jalalpure et al. (2008)

**FIGURE 3:**
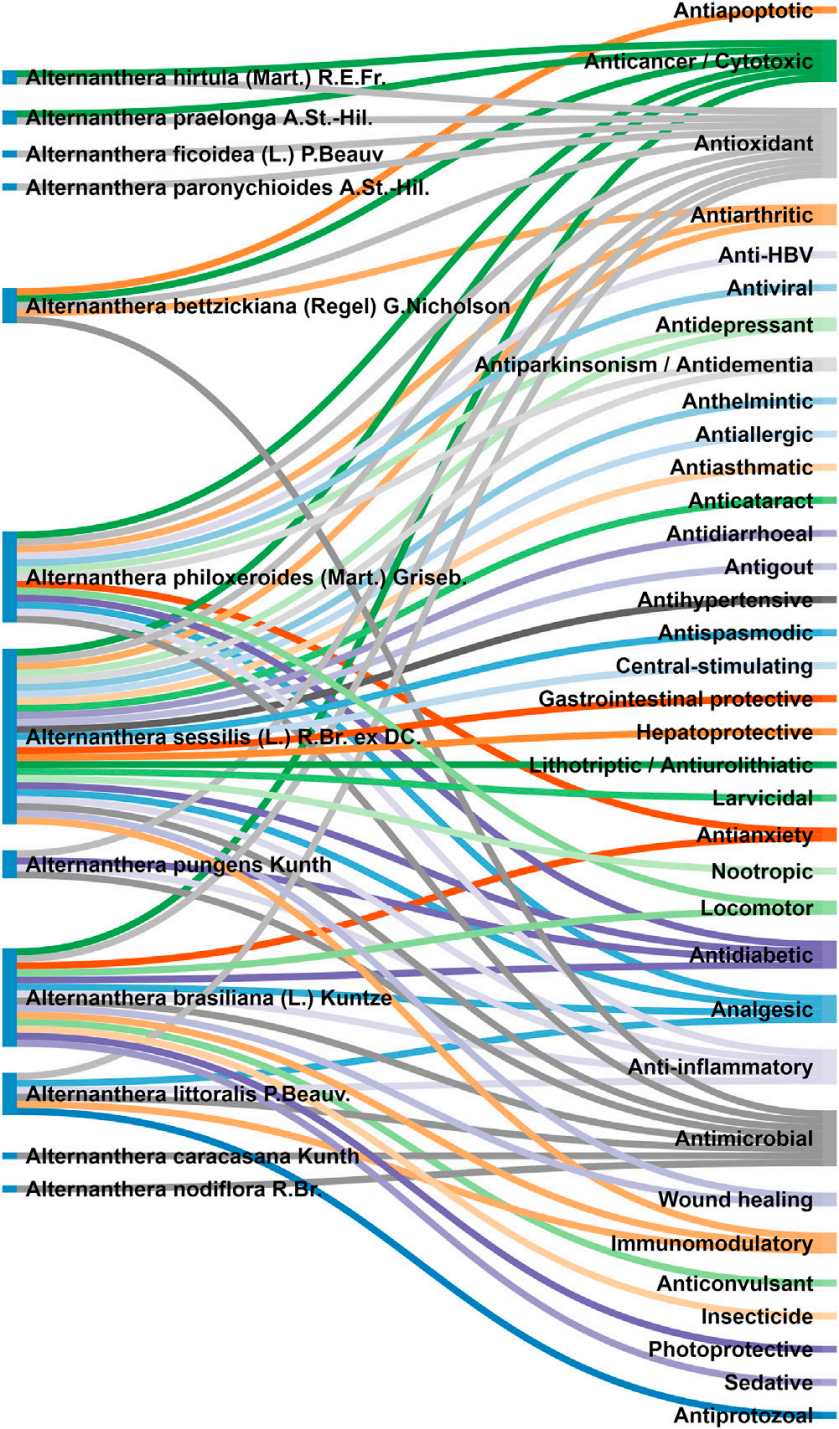
Interactive analysis mapping between various species of Alternanthera genus and their elicited pharmacological properties.

Referring to the data tabulated in Table 2, and interactive Figure 3, it is quite evident that the Alternanthera genus is having tremendous potential having polypharmacological effects. 35 different types of pharmacological effects were elicited by different species of Alternanthera genus. While the species like *Alternanthera sessilis* (L.) R.Br. ex DC., *Alternanthera brasiliana* (L.) Kuntze, and *Alternanthera philoxeroides* (Mart.) Griseb. were most widely explored, it opens up the opportunity for the researchers to explore other species of this genus.

### Analgesic Activity

Pelisoli Formagio and the team had evaluated the aqueous extract from the aerial parts of *Alternanthera brasiliana* (L.) Kuntze for its analgesic potential. 90.35% reduction of acetic acid induced contractions were observed in mice, when treated with 25 mg/kg of the aqueous extract (Pelisoli Formagio et al., 2012). Coutinho and the team had performed the formalin test in mice for assessment of analgesic effect of ethanolic extract from the leaves of *Alternanthera brasiliana* (L.) Kuntze. At 100 mg/kg, ethanolic extract was capable of reducing the edematogenic process by 64.17% (Coutinho et al., 2017). Phytoconstituents like kaempferol (Parveen et al., 2007), quercetin (Anjaneyulu and Chopra, 2003), vitexin (Zhu et al., 2016), etc may be responsible for the analgesic potential of *Alternanthera brasiliana* (L.) Kuntze.

de Santana Aquino and the team had evaluated ethanolic extract as well as isolated compound, 2″-O-α-L-rhamnopyranosylvitexin from the aerial parts of *Alternanthera littoralis* P.Beauv. for analgesic potential. Results suggested that the ethanolic extract as well as 2″-O-α-L-rhamnopyranosylvitexin are capable of exerting significant analgesic effect, most probably through the TNF pathway (de Santana Aquino et al., 2015). Since kaempferol, quercetin, and vitexin were also been reported from *Alternanthera littoralis* P.Beauv. (Figure 2), so these compounds could also attribute in analgesic potential of the extract.

Khatun and the team had prepared the methanolic extract from the whole plant part of *Alternanthera philoxeroides* (Mart.) Griseb. and evaluated for its analgesic potential in the acetic acid induced mice. They found that 400 mg/kg dose of methanolic extract was capable of reducing constrictions by 44.8%. Phytoconstituents like kaempferol (Parveen et al., 2007), quercetin (Anjaneyulu and Chopra, 2003), vitexin (Zhu et al., 2016), caffeic acid (Gamaro et al., 2011), ursolic acid (Vasconcelos et al., 2006), etc may be responsible for the analgesic potential of *Alternanthera philoxeroides* (Mart.) Griseb.

Various research teams have independently assessed the analgesic potential of *Alternanthera sessilis* (L.) R.Br. ex DC.: Mondal and the team used ethanolic extract of the leaves (Mondal et al., 2014); Mohapatra and the team used hydroethanolic extract of leaves (Mohapatra et al., 2018); Hossain and the team used methanolic extract of aerial parts (Hossain et al., 2014); while Mohaimenul and the team used ethanolic extract of aerial parts (Mohaimenul et al., 2020). It is thus quite validated that aerial parts especially leaves of *Alternanthera sessilis* (L.) R.Br. ex DC. have the analgesic potential. Various mechanisms observed by those researchers for this activity. Some of them are like inhibition of interleukins like IL-4, IL-5, and IL-13, dopaminergic and serotonergic pathways, inhibition of lipoxygenase and cyclooxygenase, etc. Along with kaempferol, vitexin, and quercetin, compounds like stigmasterol (Walker et al., 2017) may also be responsible for such analgesic effect.

### Anthelmintic Activity

Vennila and Nivetha had prepared various extracts from the leaves of *Alternanthera sessilis* (L.) R.Br. ex DC. and performed *In vitro—Pheretima Posthuma* method for assessment of anthelmintic activity. They observed that methanolic extract was the most potent and active at all the tested concentrations. The possible mechanism proposed by them was membrane lysis which subsequently led to paralysis or death of the worm (Vennila and Nivetha, 2015). On the other hand, Mondal and the team had assessed anthelmintic activity of ethanolic extract of the whole plant as well as the isolated ellagic acid by using *In vitro—*Adult motility test. They had also indicated the disruption of cell permeability, along with various other pathways and found ellagic acid a key responsible compound (Mondal et al., 2015). Other compounds that may be responsible for this pharmacological effects could be quercetin (Borges et al., 2020), β-sitosterol (Deepak et al., 2002), etc.

### Antiallergic Activity

Rayees and the team checked the antiallergic activity of 95% ethanolic extract from aerial parts of *Alternanthera sessilis* (L.) R.Br. ex DC. Studies were conducted in rat basophilic leukemia (RBL-2H3) cells. They found that the treatment with ethanolic extract resulted in nuclear factor-KB (NF-kB) dependent inhibition of cytokines like IL-6, TNF-α, IL-13, and IL-4, along with the decrease in β-hexosaminidase release (Rayees et al., 2013). Compounds like β-sitosterol (Yuk et al., 2007; Mahajan and Mehta, 2011), kaempferol (Oh et al., 2013), quercetin (Mlcek et al., 2016), vitexin (Venturini et al., 2018), stigmasterol (Antwi et al., 2018), etc may be responsible for the antiallergic activity of *Alternanthera sessilis* (L.) R.Br. ex DC.

### Antianxiety Property

Various research teams have independently assessed the antianxiety potential of *Alternanthera brasiliana* (L.) Kuntze: Pelisoli Formagio had used the aqueous extract of the leaves (Pelisoli Formagio et al., 2012); Oyemitan and the team had used the ethanolic extract of the leaves (Oyemitan et al., 2015); while Barua and the team had used the methanolic extract of the leaves (Barua et al., 2013). It is thus quite validated that the leaves of *Alternanthera brasiliana* (L.) Kuntze have the antianxiety potential. Various mechanisms observed by those researchers for this activity. Some of them are like activation of GABA receptor and 5-HT partial agonistic action. Phytomolecules like stigmasterol (Karim et al., 2021), kaempferol (Kaur et al., 2017), quercetin (Singh et al., 2013), p-coumaric acid (He Y. et al., 2021), etc may be responsible for this antianxiety property of *Alternanthera brasiliana* (L.) Kuntze.

Khamphukdee and the team had assessed ethanolic extract from the leaves of *Alternanthera philoxeroides* (Mart.) Griseb. for antianxiety potential by performing *In vivo—*Elevated plus-maze test, Light/Dark transition test, and Locomotor activity test in female mice. They observed that both the test doses i.e. 250 and 500 mg/kg/day of the extract was able to reduce the anxiety, most probably through the esterogenic pathway. Quercetin and kaempferol were detected in this plant also, so may be responsible for such antianxiety behavior.

### Antiapoptotic Activity

Wu and the team had studied the antiapoptotic potential of ethanolic extract from the whole plant of *Alternanthera bettzickiana* (Regel) G.Nicholson. They found that ethanolic extract has strong tendency to reduce apoptosis which was modulated via multiple mechanisms including reduction of reactive oxygen species, inhibition of caspase-3 and caspase-9 activation, etc. They had reported quercetin as the major compound in that extract, and they found same mechanisms when evaluated quercetin for antiapoptotic potential.

### Antiarthritic Activity

Manan and the team had studied antiarthritic potential of the ethanolic extract obtained from the aerial parts of *Alternanthera bettzickiana* (Regel) G.Nicholson using *in silico, in vitro* and *in vivo* methodologies. HPLC analysis indicated the presence of catechin, gallic acid, sinapic acid, chlorogenic acid, alpha-tocopherol, gamma-tocopherol, and quercetin. They have found that even the 250 mg/kg/day of the ethanolic extract was able to modulate the parameters suggesting the antiarthritic potential when compared with standard drug and disease control. In silico analysis suggested the strong interaction between the HPLC-analysed phytomolecules and cyclooxygenases (Manan et al., 2020).

Sunmathi and the team had studied the antiarthritic activity of ethanolic extracts obtained from the leaves of *Alternanthera philoxeroides* (Mart.) Griseb. and *Alternanthera sessilis* (L.) R.Br. ex DC. using *in vitro* methodologies. They found that 500 μg/ml of ethanolic extract of *Alternanthera philoxeroides* (Mart.) Griseb. and *Alternanthera sessilis* (L.) R.Br. ex DC. were able to stabilize the membrane by 64.92 and 75.43%, respectively. Phytomolecules like vitexin (Yang et al., 2019) and quercetin (Mamani-Matsuda et al., 2006) may be responsible for the antiarthritic activity of *Alternanthera philoxeroides* (Mart.) Griseb. and *Alternanthera sessilis* (L.) R.Br. ex DC.

### Antiasthmatic Activity

Various research teams have independently assessed the antiasthmatic potential of *Alternanthera sessilis* (L.) R.Br. ex DC.: Fathima and the team had used ethanolic extract of leaves (Fathima et al., 2016) while Saqib and Janbaz had used 70% Ethanolic extract of the whole plant and its dichloromethane and aqueous fractions (Saqib and Janbaz, 2016). This validates the applicability of *Alternanthera sessilis* (L.) R.Br. ex DC. in the treatment management of asthma. Ethanolic extract obtained from the leaves was found to reduce the leucocyte count and significantly inhibited the histamine release (Fathima et al., 2016). 70% ethanolic extract of the whole plant was found to act via calcium channel blocking mechanism (Saqib and Janbaz, 2016). Phytomolecules like kaempferol (Gong et al., 2012), vitexin (Venturini et al., 2018), quercetin (Fortunato et al., 2012), stigmasterol (Antwi et al., 2017a), chlorogenic acid (Kim et al., 2010), etc. may be key components for the antiasthamatic activity of *Alternanthera sessilis* (L.) R.Br. ex DC.

### Anticancer/Cytotoxic Property

Various research teams have independently assessed the anticancer property of *Alternanthera bettzickiana* (Regel) G.Nicholson: M Nagalingam and the team had used aqueous extract of the leaves (Nagalingam et al., 2018) while R Jothi Ramalingam and the team had used aqueous extract of leaves and silver nanoparticles and Ag-mesoporous MnO2 nanocomposite (Jothi Ramalingam et al., 2017). This validates the potential of leaves from *Alternanthera bettzickiana* (Regel) G.Nicholson and their nanoparticles in colon cancer and lung cancer. Apigenin analogues present in the *Alternanthera bettzickiana* (Regel) G.Nicholson may be responsible for the anticancer property (Madunić et al., 2018; Imran et al., 2020).

Similarly, various research teams have independently assessed the anticancer property of *Alternanthera brasiliana* (L.) Kuntze: Brochado and the team had used aqueous fraction of the ethanolic extract from the leaves. They had also isolated 6 bioactive compounds from this fraction viz. robinin, clovin, quercetin 3-O-robinobioside, kaempferol 3-O-robinobioside, kaempferol 3-O-rutinoside-7-O-a-L-rhamnopyranoside, and kaempferol 3-O-rutinoside (Brochado et al., 2003); Samudral and the team had used ethyl acetate extract obtained from the leaves (Samudrala et al., 2015). These pieces of evidence validates the anticancer potential of *Alternanthera brasiliana* (L.) Kuntze leaves. Brochado and the team found Kaempferol 3-O-robinobioside and kaempferol 3-O-rutinoside as the active phytomolecules (Brochado et al., 2003).

Independently several researches had also been conducted from various labs to assess the potential of *Alternanthera philoxeroides* (Mart.) Griseb. as anticancer agent: Zhang and the team had used the methanolic extract of the leaves and checked cytotoxicity against H9c2 cell lines. They found that even at 20 mg/ml, the methanolic extract was able to inhibit the doxorubicin induced cardiomyocyte apoptosis by more than 50%. They had also observed the presence of -carboline and quercetin (Zhang et al., 2018). Fang and the team had isolated 5 phytomolecules from the aerial parts of *Alternanthera philoxeroides* (Mart.) Griseb., and checked their inhibitory activity against Hela and L929 cell lines. While N-trans-feruloyl-3,5-dimethoxytyramine, alternanthin, N-trans-feruloyl-3-methyldopamine, and N-trans-feruloyl tyramine were found to have more than 50% inhibition at 30 μg/ml against Hela cell line, only Alternanthin B, and alternanthin were having more than 50% inhibition at 30 μg/ml against L929 cell line (Fang et al., 2007). Fang and the team had further isolated 4 more compounds from the aerial parts of *Alternanthera philoxeroides* (Mart.) Griseb. The triterpenoidal saponins, Philoxeroidesides A, B, C, and D were found to inhibit SK-N-SH cell line with an IC50 of 51, 118.69, 60.6, and 37.29 μg/ml, respectively, while inhibited HL60 cell line with an IC50 of 185.29, 185.57, 271.45, and 45.93 μg/ml, respectively. Philoxeroidesides D was found to be quite potential against both the cell lines (Fang J.-B. et al., 2009). In another study performed by Correa and the team where they had used ethanolic extracts obtained from the whole plant of *Alternanthera philoxeroides* (Mart.) Griseb.; *Alternanthera hirtula* (Mart.) R.E.Fr., and *Alternanthera praelonga* A.St.-Hil. They tested the ethanolic extracts against various human cancer cells lines including that from melanoma, breast, kidney, lung, prostate, ovary, colon, leukemia, along with non-cancer cell line from green monkey kidney. Out of all the cancer cell lines, these ethanolic extracts were being able to be found potent only against the leukemia cell line, K562 (Correa et al., 2016).

Several researchers have independently assessed the potential of *Alternanthera sessilis* (L.) R.Br. ex DC. for the management of cancer: Jain and the team had used the methanolic extract of leaves (Jain et al., 2016); Firdhouse and Lalitha had used silver nanoparticles of the aqueous extract (Firdhouse and Lalitha, 2013); Qian and the team had used gold nanoparticles of the aqueous extract of leaves (Qian et al., 2019); D Suganya and the team had used aqueous extract of leaves and stems (Suganya et al., 2019); Pathak and the team had used n-hexane and methanolic extracts of aerial parts (Pathak et al., 2020); Mohaimenul and the team had used ethanolic extract of aerial parts (Mohaimenul et al., 2020); Yap and the team had used ethanolic, 70% ethanolic, 80% methanolic, ethyl acetate, and aqueous extracts of the whole plant (Yap et al., 2019); Sathishkumar and the team had used silver nanoparticles of the aqueous extract of leaves (Sathishkumar et al., 2016); Arulselvan and the team had used ethanolic extract of aerial parts, stem, and leaves (Arulselvan et al., 2018); while Guerra and the team aqueous extract of aerial parts (Guerra et al., 2003). All these studies indicated the true potential of *Alternanthera sessilis* (L.) R.Br. ex DC. for the treatment and management of cancer, with leaving no doubt in it. Phytomolecules present in the *Alternanthera sessilis* (L.) R.Br. ex DC. like kaempferol (Imran et al., 2019), vitexin (Liu et al., 2019; Lee et al., 2020), quercetin (Rauf et al., 2018), stigmasterol (Ali et al., 2015), chlorogenic acid (Barahuie et al., 2017), campesterol (Bae et al., 2021), and β-sitosterol (Pradhan et al., 2016), etc. may be responsible for this anticancer property.

### Anticataract Property

Kota and the team had checked the anticataract property of ethyl acetate extract obtained from the leaves of *Alternanthera sessilis* (L.) R.Br. ex DC. Cataract induced in eye lenses of the chicks were subjected for the treatment with 100, 200, and 400 mg of ethyl acetate extract, followed by analysis of lipid peroxidation and Na^+^- K^+^ ATPases. They found that 100 and 200 mg ethyl acetate treatment will lead to decrease in malondialdehyde and increase in the inorganic phosphorous content (Kota et al., 2017). Phytomolecules like quercetin (Lan et al., 2020), chlorogenic acid (Kim et al., 2011), and β-sitosterol (Haroon et al., 2020) may be responsible for this anticataract property of *Alternanthera sessilis* (L.) R.Br. ex DC.

### Anticonvulsant Activity

Independently several researches had also been conducted from various labs to assess the potential of *Alternanthera brasiliana* (L.) Kuntze as anticonvulsant agent: Oyemitan and the team had used the ethanolic extract of leaves (Oyemitan et al., 2015); Schallenberger and the team had also used the ethanolic extract of leaves (Schallenberger et al., 2017); while Barua and the team had used the methanolic extract of leaves (Barua et al., 2013). This had validated the anticonvulsant potential of the leaves of *Alternanthera brasiliana* (L.) Kuntze. Various mechanisms elucidated by them are like modulation of GABAergic system, controlling the entry of calcium and sodium ions in the cells, and glycine regulation in spinal cord (Oyemitan et al., 2015). Phytomolecules like vitexin (de Oliveira et al., 2020), quercetin (Nassiri-Asl et al., 2014; Nieoczym et al., 2014), stigmasterol (Karim et al., 2021), chlorogenic acid (Aseervatham et al., 2016), and ferulic acid (Hassanzadeh et al., 2017) may be responsible for the antiepileptic effect of *Alternanthera brasiliana* (L.) Kuntze.

### Antidepressant Activity

Khamphukdee and the team had assessed the antidepressant effect of the ethanolic extract obtained from the leaves of *Alternanthera philoxeroides* (Mart.) Griseb. They found that the extract was having significant antidepressant effect modulated through the estrogenic pathway (Khamphukdee et al., 2018). Phytomolecules like quercetin (Anjaneyulu and Chopra, 2003), vitexin (Can et al., 2013), β-sitosterol (Zhao et al., 2016), p-coumaric acid (Lee et al., 2018), caffeic acid (Monteiro et al., 2020), ursolic acid (Machado et al., 2012; Singla et al., 2017), and malic acid (Gómez-Moreno et al., 2013) may be responsible for the antidepressant activity of *Alternanthera philoxeroides* (Mart.) Griseb.

Gupta and K. Singh had evaluated the antidepressant activity of methanolic extract obtained from the leaves of *Alternanthera sessilis* (L.) R.Br. ex DC. They had observed that the antidepressant effect of the methanolic extract was acting via interaction with adrenergic, dopaminergic serotonergic, and GABAergic system (Gupta and Singh, 2014). Phytomolecules like quercetin, vitexin, and p-coumaric acid had also been reported from *Alternanthera sessilis* (L.) R.Br. ex DC., along with other antidepressant agents like kaempferol (Park et al., 2010b), ferulic acid (Chen et al., 2014) and chlorogenic acid (Park et al., 2010a). These phytomolecules may be responsible for the antidepressant activity of *Alternanthera sessilis* (L.) R.Br. ex DC.

### Antidiabetic Activity

Reza and the team had assessed the antidiabetic potential of 80% ethanolic extracts obtained from the stem and leaves of *Alternanthera brasiliana* (L.) Kuntze. They found that the ethanolic extracts were being able to significantly modulate the biochemical parameters like blood glucose, lipid peroxidation, and free radicals in the alloxan-induced diabetic Swiss albino mice (Reza et al., 2019). Phytomolecules like kaempferol (Ibitoye et al., 2018), quercetin (Vessal et al., 2003), stigmasterol (Wang et al., 2017; Singla and Shen, 2020), p-coumaric acid (Amalan et al., 2016), ferulic acid (Narasimhan et al., 2015), and chlorogenic acid (Ong et al., 2013) may be responsible for the antidiabetic potential of *Alternanthera brasiliana* (L.) Kuntze.

Khatun and the team as well as Bhattacherjee and the team had independently assessed the antidiabetic activity of *Alternanthera philoxeroides* (Mart.) Griseb. Various important mechanisms had been observed by them including regeneration of the β-cells of the pancreas, alpha-glucosidase inhibition, as well as the inhibition of the glucose absorption from the gut wall (Khatun et al., 2012; Bhattacherjee et al., 2014). Compounds like quercetin and p-coumaric acid had been reported from *Alternanthera philoxeroides* (Mart.) Griseb., and may be responsible for such antidiabetic effect.

Mourya and the team had used aqueous and ethanolic extracts obtained from the whole plant of *Alternanthera pungens* Kunth for the assessment of antidiabetic potential. Dose dependent antidiabetic activity was observed by them when studied in alloxan-induced diabetic Wistar rats. Phytocompounds like camphene (Hachlafi et al., 2021), camphor (Drikvandi et al., 2020), geraniol (Babukumar et al., 2017), and limonene (Murali and Saravanan, 2012) may be responsible for such antidiabetic property of *Alternanthera pungens* Kunth.

Independently several researches had also been conducted from various labs to assess the potential of *Alternanthera sessilis* (L.) R.Br. ex DC. as antidiabetic agent: Kumar and the team had used aqueous and ethanolic extracts of aerial parts (Kumar S. M. et al., 2011); Tan and Kim had used hexane, ethyl acetate, and aqueous fractions of aerial parts (Tan and Kim, 2013); Hossain and the team had used methanolic extract of aerial parts (Hossain et al., 2014); Sundar and the team had used petroleum ether extract of leaves (Sundar et al., 2019); Das and the team had used 95% ethanolic extract of the whole plant (Das et al., 2015); Rao and the team had used ethanolic extract of the whole plant (Rao et al., 2011); Manalo and the team had used n-hexane, ethyl acetate, and water fractions of the methanolic extract of leaves (Manalo et al., 2020); Mohaimenul and the team had used ethanolic extract of aerial parts (Mohaimenul et al., 2020); Tiwari and the team had used the juice (Tiwari et al., 2013); Chai and the team had used hexane, chloroform, ethyl acetate, butanol, and aqueous fractions of methanolic extracts of leaves and callus (Chai et al., 2016). Plenty of evidences obtained from the above researches leaved no doubt in that fact that *Alternanthera sessilis* (L.) R.Br. ex DC. possesses antidiabetic properties. Various mechanisms demonstrated by different preparations from *Alternanthera sessilis* (L.) R.Br. ex DC., including but not limited to modulation of insulin sensitivity, improvement in pancreatic insulin secretion, reduction in blood glucose level, inhibition of α-glucosidase enzyme, etc. Phytomolecules like kaempferol (Ibitoye et al., 2018), quercetin (Vessal et al., 2003), stigmasterol (Wang et al., 2017; Singla and Shen, 2020), 4-hydroxybenzoic acid (Peungvicha et al., 1998), β-sitosterol (Ponnulakshmi et al., 2019), ellagic acid (Fatima et al., 2015), ferulic acid (Narasimhan et al., 2015), and chlorogenic acid (Ong et al., 2013) may be responsible for the antidiabetic potential of *Alternanthera sessilis* (L.) R.Br. ex DC.

### Antidiarrheal Activity

Zavala and the team had evaluated the antidiarrheal property of hexane, chloroform, methanolic, and aqueous extracts obtained from the whole plant of *Alternanthera sessilis* (L.) R.Br. ex DC. They had observed that out of all extracts, methanolic and aqueous extracts had shown significant inhibition of castor oil-induced diarrhea. Methanolic extract was further found to inhibit normal defecation in mice also. Peristaltic movement was also modulated by the methanolic extract (Zavala et al., 1998). Phytomolecules like quercetin (Lozoya et al., 1994; Song et al., 2011; Shi et al., 2020), β-sitosterol (Ding et al., 2018), ellagic acid (Chen et al., 2020), ferulic acid (Hu et al., 2021), and chlorogenic acid (Zhang et al., 2017; Chen et al., 2018) may be responsible for the antidiarrheal property of *Alternanthera sessilis* (L.) R.Br. ex DC.

### Antigout Activity

Chong and Loh had assessed the antigout potential of methanolic extract obtained from the aerial parts of *Alternanthera sessilis* (L.) R.Br. ex DC. Methanolic extract was able to inhibit xanthine oxidase enzyme with an IC50 of 557.77 μg/ml (Chong and Loh, 2020). Phytomolecules like kaempferol (Wang et al., 2015d), quercetin (Bindoli et al., 1985), stigmasterol (Chiang and Chen, 2008), ellagic acid (Sun et al., 2021), ferulic acid (Nile et al., 2016), and chlorogenic acid (Wang et al., 2009) may be responsible for the antigout potential of *Alternanthera sessilis* (L.) R.Br. ex DC.

### Anti-Hepatitis B Virus Activity

Li and the team had isolated C-boivinopyranosyl flavones from *Alternanthera philoxeroides* (Mart.) Griseb. and found that luteolin-6-*C*-β-d-boivinopyranosyl-3′-*O*-β-d-glucopyranoside, chrysoeriol-6-*C*-β-d-Boivinopyranosyl-4′-*O*-β-d-glucopyranoside, and luteolin-6-*C*-β-d-boivinopyranosyl-4′-*O*-β-d-glucopyranoside were strongly inhibiting the viral antigen, HBsAg in HBV-infected HepG2.2.15 with an IC50 of 28.65, 22.20, and 31.54 µM, respectively (Li et al., 2016).

### Antihypertensive Activity

Saqib and Janbaz had evaluated the antihypertensive effect of 70% Ethanolic extract of the whole plant and its dichloromethane and aqueous fractions from *Alternanthera sessilis* (L.) R.Br. ex DC. The *in vivo* studies suggested that the ethanolic extract was capable to reducing both the systolic and the diastolic pressure. Phytomolecules like kaempferol (Ahmad et al., 1993; Binang and Takuwa, 2021), quercetin (Perez-Vizcaino et al., 2009; Binang and Takuwa, 2021), vitexin (Xue et al., 2020), β-sitosterol (Olaiya et al., 2014), ellagic acid (Berkban et al., 2015), ferulic acid (Li et al., 2020), and chlorogenic acid (Zhao et al., 2011) may be responsible for the antihypertensive potential of *Alternanthera sessilis* (L.) R.Br. ex DC.

### Anti-Inflammatory Activity

Pelisoli Formagio and the team had performed the *in vivo* studies to assess the anti-inflammatory activity of the aqueous extract obtained from the leaves of *Alternanthera brasiliana* (L.) Kuntze while P Shivashankar and the team had used the methanolic extract obtained from the leaves. Pelisoli Formagio and the team had observed the significant decrease in the polymorphonuclear cells as well as increase in the mononuclear cells in rat’s exudate after treated with the aqueous extract, while P Shivashankar and the team found the reduction in the colon weight in acetic acid-induced colitis model of adult Wistar albino rats after treatment with the methanolic extract (Pelisoli Formagio et al., 2012; P et al., 2016). Phytomolecules like kaempferol (Devi et al., 2015), quercetin (Lesjak et al., 2018), stigmasterol (Morgan et al., 2021), p-coumaric acid (Pragasam et al., 2012), ferulic acid (Ozaki, 1992), and chlorogenic acid (Hwang et al., 2013) may be responsible for the anti-inflammatory potential of *Alternanthera brasiliana* (L.) Kuntze.

de Santana Aquino and the team had evaluated anti-inflammatory activity of ethanolic extract of aerial parts and the isolated compound, 2″-O-α-L-rhamnopyranosylvitexin from *Alternanthera littoralis* P.Beauv. They found that the ethanolic extract was able to reduce the paw edema as well as capable to reducing leukocyte migration. In addition to these, the isolated compound was also able to reduce protein leakage into the pleural cavity (de Santana Aquino et al., 2015). Other phytomolecules that could be responsible for the anti-inflammatory activity of the ethanolic extract will be kaempferol, quercetin, stigmasterol, etc.

Sunmathi and the team had evaluated anti-inflammatory activity of ethanolic extract obtained from the leaves of *Alternanthera philoxeroides* (Mart.) Griseb. Dose dependent membrane stabilization was observed. Phytomolecules like quercetin (Lesjak et al., 2018), vitexin (Rosa et al., 2016), β-sitosterol (Loizou et al., 2010), p-coumaric acid (Pragasam et al., 2012), caffeic acid (da Cunha et al., 2009), ursolic acid (Baricevic et al., 2001), and malic acid (Obertreis et al., 1996) may be responsible for the anti-inflammatory activity of *Alternanthera philoxeroides* (Mart.) Griseb.

Franck and the team had evaluated the anti-inflammatory activity of aqueous extract obtained from the leaves of *Alternanthera pungens* Kunth. They had observed the decreased level of histamine release, serotonin and kinin, prostaglandin, proteases, lysosomes, and protein C-reactive. Phytomolecules like α-pinene (Kim et al., 2015), myrcene (Rufino et al., 2015), limonene (Rufino et al., 2015), choline (Rowley et al., 2010), rhein (Gao et al., 2014), linalool (Peana et al., 2002), geraniol (Ye et al., 2019), and camphor (Ehrnhöfer-Ressler et al., 2013) which were reported earlier in *Alternanthera pungens* Kunth., may be responsible for this anti-inflammatory effect.

Independently several researches had also been conducted from various labs to assess the potential of *Alternanthera sessilis* (L.) R.Br. ex DC. as anti-inflammatory agent: Sunmathi and the team had used ethanolic extract obtained from the leaves (Sunmathi et al., 2016); Muniandy and the team had used 90% ethanolic extract of stems (Muniandy et al., 2018a); Sundar and the team had used petroleum ether and methanolic extracts of leaves (Sundar et al., 2019); Kassuya and the team had used Ethanolic extract of whole plant (EEAT) as well as the isolated molecule, 2″-O-β-D-glucopyranosyl-vitexin (Kassuya et al., 2021); Biella and the team had used aqueous extract of the whole plant (Biella et al., 2008). Plenty of evidences obtained from the above researches leaved no doubt in that fact that *Alternanthera sessilis* (L.) R.Br. ex DC. possesses anti-inflammatory properties. Various mechanisms demonstrated by different preparations from *Alternanthera sessilis* (L.) R.Br. ex DC., including but not limited to cyclooxygenase -1 and -2 inhibition (Biella et al., 2008), modulating NF- κB pathway (Muniandy et al., 2018a), leukocyte migration (Kassuya et al., 2021), etc. Phytomolecules like kaempferol (Devi et al., 2015; Pizzo et al., 2018), quercetin (Lesjak et al., 2018), vitexin (Rosa et al., 2016), stigmasterol (Morgan et al., 2021), β-sitosterol (Loizou et al., 2010), 4-hydroxybenzoic acid (Winter et al., 2017), ellagic acid (Corbett et al., 2010), ferulic acid (Ozaki, 1992), campesterol (Moreno-Anzúrez et al., 2017), spinasterol (Jeong et al., 2010), β-carotene (Uteshev et al., 2000), p-coumaric acid (Pragasam et al., 2012), ricinoleic acid (Vieira et al., 2001), and chlorogenic acid (Hwang et al., 2013) may be responsible for the anti-inflammatory potential of *Alternanthera sessilis* (L.) R.Br. ex DC.

### Antimicrobial Activity

Independently, several research teams had evaluated the antimicrobial effects of the leaves of *Alternanthera bettzickiana* (Regel) G.Nicholson: Vidhya and the team had used hexane, chloroform, ethyl acetate, methanolic, and aqueous extracts of leaves (Vidhya et al., 2015); R, Jothi Ramalingam and the team had used aqueous extract of leaves and silver nanoparticles and Ag-mesoporous MnO2 nanocomposite (Jothi Ramalingam et al., 2017); Nagalingam and the team had used the aqueous extract obtained from leaves (Au-NP) (Nagalingam et al., 2018). These research were focused on leaves and somehow validated the antimicrobial property of it. Various mechanisms elucidated were like cell wall lysis, protein synthesis inhibition, and topoisomerase inhibition, etc (Vidhya et al., 2015; Jothi Ramalingam et al., 2017; Nagalingam et al., 2018). Phytocompounds like apigenin analogs (Koo, 2003; Thirukumaran et al., 2019) may be responsible for this antimicrobial property of *Alternanthera bettzickiana* (Regel) G.Nicholson.

Coutinho and the team had evaluated the antimicrobial property of ethanolic extract obtained from the leaves of *Alternanthera brasiliana* (L.) Kuntze. They had observed that though the ethanolic extract as such was having insignificant potential, but it elicited significant synergetic potential when combined with gentamycin and tested against *Staphylococcus aureus*, *Escherichia coli*, and *Pseudomonas aeruginosa* (Coutinho et al., 2017). Johann and the team had also performed the antimicrobial experiments on the ethanolic extract obtained from the aerial parts of *Alternanthera brasiliana* (L.) Kuntze, and they had also observed that the extract was inactive against various murine macrophages and fungal strains (Johann et al., 2010). Other research team like that of Akachukwu and Uchegbu had also reported mild activity of the ethanolic extract obtained from its leaves (Akachukwu and Uchegbu, 2016) while Kumar and the team noticed significant activity elicited by the silver nanoparticles obtained from the leaves aqueous extract (Kumar et al., 2014).

Canales-Martínez and the team had evaluated the antimicrobial effect of the hexane, chloroform, methanolic, acetone, and ethyl acetate extracts obtained from the aerial parts of *Alternanthera caracasana* Kunth and also isolated a bioactive compound, 7-methoxycoumarin. They observed that the ethyl acetate extract as well as 7-methoxycoumarin were active against various Gram-positive and Gram-negative bacterial strains, but inactive against *Candida albicans* (Canales-Martínez et al., 2008). Phytochemical profiling of *Alternanthera caracasana* Kunth is still not done, leaving a scope for the researchers.

Gasparetto and the team had used crude hexane and ethanolic extract obtained from the leaves of *Alternanthera littoralis* P.Beauv., and assessed them for their antimicrobial potential. They noticed that the antifungal activity was exhibited by the crude extracts only when combined with photo-irradiation by a diode laser (Gasparetto et al., 2010). Phytocompounds like kaempferol (del Valle et al., 2016), stigmasterol (Alawode et al., 2021), hydroxytyrosol (Bisignano et al., 1999), quercetin (Gatto et al., 2002), vitexin (Das et al., 2016), and uridine (Wiegmann et al., 2016) which were reported earlier from *Alternanthera littoralis* P.Beauv., may be responsible for such antimicrobial effects.

Feka and the team had studied the antimicrobial property of the aqueous and methanolic extracts obtained from the whole plant of *Alternanthera nodiflora* R.Br. They found that the methanolic extract was having significant antimicrobial activity against bacterial and yeast strains, but inactive against mould test strain (Feka et al., 2014). Phytochemical profiling of *Alternanthera nodiflora* R.Br. is still not done, leaving a scope for the researchers.

Independently several research teams had evaluated the antimicrobial potential of *Alternanthera philoxeroides* (Mart.) Griseb.: Bhattacherjee and the team had used methanol-soluble fraction obtained from the leaves (Bhattacherjee et al., 2014); Rawani and the team had used aqueous and chloroform: methanol (1:1) extracts of leaves (Rawani et al., 2011); Pulipati and the team had used ethanolic extract obtained from the leaves (Pulipati et al., 2016); Akbar and the team had used methanolic extract of leaves, stem and roots as well as their n-hexane, chloroform and ethyl acetate fractions (Akbar et al., 2021); while Pulipati and Babu had used the methanolic extract of leaves (Pulipati and Babu, 2020). These independent researches left no doubt and validated the antimicrobial feature of *Alternanthera philoxeroides* (Mart.) Griseb. They had reported multiple mechanisms of actions like bacterial cell wall lysis and protein synthesis inhibition (Bhattacherjee et al., 2014; Pulipati et al., 2016; Pulipati and Babu, 2020). Phytomolecules like quercetin (Gatto et al., 2002), vitexin (Das et al., 2016), β-sitosterol (Ododo et al., 2016), stigmasterol (Alawode et al., 2021), p-coumaric acid (Boz, 2015), caffeic acid (Lima et al., 2016), luteolin analogs (Chiruvella et al., 2007; Qian et al., 2020), chrysoeriol analogs (Jang et al., 2020), malic acid (Raybaudi-Massilia et al., 2009), β-carboline (Arshad et al., 2008; Suzuki et al., 2018), ursolic acid (Collins and Charles, 1987), oleanolic acid (Horiuchi et al., 2007), azelaic acid (Leeming et al., 1986), phytol (Pejin et al., 2014), and rubiadin (Marioni et al., 2016) which were earlier reported from *Alternanthera philoxeroides* (Mart.) Griseb., may be responsible for this antimicrobial property.

Jakhar and Dahiya had studied the aqueous, acetone, ethanolic, and petroleum ether extracts obtained from the aerial parts of *Alternanthera pungens* Kunth for assessment of antimicrobial effect against various bacterial and fungal strains. They found that all the extracts were having potential as antibacterial, but the antifungal property was exhibited by only acetone and aqueous extracts. Noticed mechanisms were inhibition of DNA replication as well as blocking of cellular respiration. Phytochemicals like choline (Siopa et al., 2016), rhein (Joung et al., 2012), limonene (Vuuren and Viljoen, 2007), α-curcumene (Santos da Silva et al., 2015), geraniol (Lira et al., 2020), linalool (Park S.-N. et al., 2012), camphor (Masry et al., 2021), myrcene (Chaves-Quirós et al., 2020), and α-pinene (Dhar et al., 2014; Cloeckaert et al., 2015) which were earlier reported from *Alternanthera pungens* Kunth, may be responsible for such antimicrobial action.

Plenty of independent researches have been extracted from the literature, covering evaluation of antimicrobial activity of *Alternanthera sessilis* (L.) R.Br. ex DC.: Osuna and the team had used hexane and methanolic extracts obtained from the aerial parts (Osuna et al., 2008); Jalalpure and the team had used petroleum ether (40–60°C), chloroform, acetone, methanolic, and aqueous extracts of leaves (Jalalpure et al., 2008); Monroy and Limsiaco had used aqueous, ethanolic, and acetone extracts obtained from leaves (Monroy and Limsiaco, 2016); Niraimathi and the team had used silver nanoparticles of aqueous extract of leaves (Niraimathi et al., 2013); Rajamurugan and the team had used ethanolic extract obtained from the leaves (Rajamurugan et al., 2013); D Suganya and the team had used aqueous extract of leaves and stems (Suganya et al., 2019); Kota and the team had used petroleum ether, ethyl acetate, chloroform, and methanolic extract obtained from the leaves (Kota et al., 2017); Sundar and the team had used petroleum ether and methanolic extracts of leaves (Sundar et al., 2019); while Salvador and the team had used hexane and ethanolic extracts obtained from the adult plants (Salvador et al., 2009). These studies clearly concluded that *Alternanthera sessilis* (L.) R.Br. ex DC. possesses antimicrobial properties. Several mechanisms elucidated by them are like cell membrane lysis, prevention of protein synthesis, blocking cellular respiration, inhibition of DNA replication, deprivation of iron for microbial growth, etc (Osuna et al., 2008; Salvador et al., 2009; Rajamurugan et al., 2013; Monroy and Limsiaco, 2016; Kota et al., 2017; Suganya et al., 2019). Phytomolecules like Vitexin (Das et al., 2016), Kaempferol (del Valle et al., 2016), Quercetin (Gatto et al., 2002), Kaempferol-7- O-glucoside (Singh et al., 2011), Stigmasterol (Alawode et al., 2021), β-Sitosterol (Ododo et al., 2016), Ellagic acid (Abuelsaad et al., 2013; De et al., 2018), Ferulic acid (Shi et al., 2016), p-Coumaric acid (Boz, 2015), 4-Hydroxybenzoic acid (Cho J.-Y. et al., 2014), 2,5-Dihydroxybenzoic acid (Kim et al., 2007), Chlorogenic acid (Li et al., 2013; Kabir et al., 2014), Ionone (Mikhlin et al., 1983), β-Carotene (Hayashi et al., 2012), and Ricinoleic acid (Novak et al., 1961) which were earlier reported from *Alternanthera sessilis* (L.) R.Br. ex DC. may be responsible for its antimicrobial property.

### Antioxidant Activity

Petrus and the team had evaluated the antioxidant activity of the 80% aqueous methanolic extract obtained from the flowers of *Alternanthera bettzickiana* (Regel) G.Nicholson. They had observed that the extract possessed radical scavenging and ferrous ion chelating properties (Petrus A. et al., 2014). On the other hand, Vidhya and the team had evaluated the antioxidant activity of the hexane, chloroform, ethyl acetate, methanolic, and aqueous extracts obtained from the leaves *Alternanthera bettzickiana* (Regel) G.Nicholson. They observed that out of all, methanolic extract was exhibiting stronger radical scavenging activity (Vidhya et al., 2015). Phytomolecules like apigenin analogs (Prince Vijeya Singh et al., 2004) which were earlier reported from *Alternanthera bettzickiana* (Regel) G.Nicholson, may be responsible for this antioxidant potential.

Independently, several research teams had investigated the antioxidant potential of *Alternanthera brasiliana* (L.) Kuntze: Reza and the team had used 80% ethanolic extract of stem and leaves (Reza et al., 2019); Enechi and the team had used ethanolic extract of leaves (Enechi et al., 2013); Chandran R had used methanolic extract of leaves (Chandran, 2017); Attaugwu and Uvere had used ethanolic extract of leaves (Attaugwu and Uvere, 2017); Pereira and the team had used ethanolic extract and its dichloromethane, ethyl acetate, n-butanolic fractions of leaves (Pereira et al., 2013); Araujo and the team had used ethanolic extract of aerial parts and its hexane, chloroform, and ethyl acetate fractions (Araujo et al., 2014); while Akachukwu and Uchegbu had used ethanolic extract of leaves (Akachukwu and Uchegbu, 2016). These pieces of evidence increase the credibility of *Alternanthera brasiliana* (L.) Kuntze as antioxidant. Phytoconstituents like Ligustroflavone (Kang et al., 2021), Vitexin (An et al., 2012), Kaempferol (Park et al., 2006), Quercetin (Zhang et al., 2011), Tricin (Duarte-Almeida et al., 2007), Quercetin 3-β-D-glucoside (Niranjan Panat et al., 2015), Isorhamnetin-3-O-robinobioside (Boubaker et al., 2012), Stigmasterol (Liang et al., 2020), β-Sitosterol (Gupta et al., 2011), Ferulic acid (Graf, 1992), p-Coumaric acid (Kiliç and Yeşiloğlu, 2013), 4-Hydroxybenzoic acid (Velika and Kron, 2012), 2,5-Dihydroxybenzoic acid (Calderón Guzmán et al., 2007), Chlorogenic acid (Sato et al., 2011), Dopamine-betaxanthin (Cai et al., 2003), and 3-Methoxytyramine-betaxanthin (Cai et al., 2003) which were earlier reported from *Alternanthera brasiliana* (L.) Kuntze, may be responsible for its antioxidant property.

Patil and Kore had evaluated the antioxidant property of methanolic extracts obtained from different parts viz. leaves, stem, and roots of *Alternanthera ficoidea* (L.) P.Beauv. They had observed that out of all, the methanolic extract from the roots was having most potent antioxidant activity (Patil and Kore, 2019). To the best of our knowledge, the phytochemial characterization of *Alternanthera ficoidea* (L.) P.Beauv. was not yet done, leaving an ample scope for the researchers.

Koolen and the team had isolated seven phytoconstituents from the aerial sections of *Alternanthera littoralis* P.Beauv. and evaluated them for the antioxidant potential using *In vitro*—ORAC assay. They had observed that out of all compounds, Alternamide B was the most significant one as antioxidant. Researchers had further suggested the catechol scaffold as a pharmacophore for this activity (Koolen et al., 2017).

Two independent research teams had evaluated the antioxidant potential of *Alternanthera paronychioides* A.St.-Hil.: Wu and the team had used methanolic, ethanolic, and aqueous extracts of the whole plant (Wu et al., 2013) while Tukun and the team had used aqueous extract obtained from the leaves (Tukun et al., 2014). These preliminary studies signifies the role of *Alternanthera paronychioides* A.St.-Hil. as antioxidant. To the best of our knowledge, the phytochemial characterization of *Alternanthera paronychioides* A.St.-Hil. was not yet done, leaving an ample scope for the researchers.

Bhattacherjee and the team had evaluated the antioxidant activity of methanol soluble fraction obtained from the leaves of *Alternanthera philoxeroides* (Mart.) Griseb. (Bhattacherjee et al., 2014). while Correa and the team had used ethanolic extracts of the whole plant (Correa et al., 2016). These preliminary studies suggested that the *Alternanthera philoxeroides* (Mart.) Griseb. is worthy of further investigation as antioxidant. Phytomolecules like Luteolin and luteolin analogs (Romanova et al., 2001), Chrysoeriol analogs (Mishra et al., 2003), Vitexin (An et al., 2012), Quercetin (Zhang et al., 2011), β-Sitosterol (Gupta et al., 2011), Δ5-Stigmasterol (Liang et al., 2020), Ursolic acid (Bobé et al., 2012; do Nascimento et al., 2014), Oleanolic acid and Oleanolic acid analogs (Wang et al., 2010), Calenduloside E (Tang et al., 2019), Caffeic acid (Gulcin, 2006), Quinic acid (Pero et al., 2009), p-Coumaric acid (Kiliç and Yeşiloğlu, 2013), Rubiadin (Tripathi et al., 1997), β-Carboline (Moura et al., 2007), Malic acid (Jin et al., 2016), Azelaic acid (Muthulakshmi and Saravanan, 2013), Cycloeucalenol (Wang W. et al., 2015), Phytol (Santos et al., 2013), and Pheophytin A (Endo et al., 1985) which were previously been reported from *Alternanthera philoxeroides* (Mart.) Griseb., may be responsible for this antioxidant property.

Several research teams have independently assessed the antioxidant potential of *Alternanthera pungens* Kunth: Mourya and the team had used ethanolic and aqueous extracts obtained from the leaves (Mourya et al., 2019); Franck and the team had used aqueous extract of leaves (Franck et al., 2016); while Jakhar and Dahiya had used aqueous, acetone, ethanolic, and petroleum ether extracts of aerial parts (Jakhar and Dahiya, 2017). These studies validated the antioxidant potential of *Alternanthera pungens* Kunth. Various phytochemicals like Limonene (Roberto et al., 2009), Geraniol (Aytac et al., 2016), Linalool (Duarte et al., 2016), Camphor (Drikvandi et al., 2020), Myrcene (Khalili et al., 2020), Camphene (Tiwari and Kakkar, 2009), and α-pinene (Aydin et al., 2013) which were reported earlier from *Alternanthera pungens* Kunth, may be responsible for its antioxidant action.

While going through literature, we have found enough pieces of evidences reporting and validating the antioxidant property of *Alternanthera sessilis* (L.) R.Br. ex DC.: Borah and the team had used 90% methanolic, 70% acetone, 80% ethanolic extracts of leaves and stems (Borah et al., 2011); Chai and the team had used hexane, chloroform, ethyl acetate, butanolic, and aqueous fractions of leaves and callus methanol extracts (Chai et al., 2016); Sharma and the team 30% hydroethanolic extract of the whole plant (Sharma et al., 2013); Khan and the team had used separate Methanolic and hexane extracts of leaves and stems (Khan et al., 2018); Azizah and the team had used ethanolic and aqueous extracts of aerial parts (Azizah et al., 2015); Muniandy and the team had used 90% hydroethanolic extract of stem (Muniandy et al., 2018b); Othman and the team had used ethanolic and aqueous extracts of aerial parts (Othman et al., 2016); Tiwari and the team had used juice (Tiwari et al., 2013); Rajamurugan and the team had used ethanolic extract of leaves (Rajamurugan et al., 2013); Jain and the team had used methanolic extract of leaves (Jain et al., 2016); Suganya and the team had used aqueous extract of leaves and stems (Suganya et al., 2019); Mohd Hazli and the team had used hexane, ethyl acetate, ethanolic, and aqueous extracts of leaves and stem (Mohd Hazli et al., 2019); Niraimathi and the team had used silver nanoparticles from aqueous extract of leaves (Niraimathi et al., 2013); Yap and the team had used 100% ethanolic, 70% ethanolic, 80% methanolic, ethyl acetate, and aqueous extracts of the whole plant (Yap et al., 2019); Kota and the team had used petroleum ether, ethyl acetate, chloroform, and methanolic extract of leaves (Kota et al., 2017); Sundar and the team had used petroleum ether and methanolic extracts of leaves (Sundar et al., 2019); Pathak and the team had used n-hexane and methanolic extracts of aerial parts (Pathak et al., 2020); Khan and the team had used the volatile oil of leaves and flowers (Khan et al., 2016); while Salvador and the team had used ethanolic extract and its four fractions; Acacetin 8-c-[*α*-L-rhamnopyranoyl-(1→2)-*β*-D-glucopyranoside]; 2″-*O*-α-L-rhamnopyranosyl-vitexin; 2″-*O*-*β*-D-glucopyranosyl vitexin and Vitexin (Salvador et al., 2006). Results from these researches left no doubt in the credibility and applicability of *Alternanthera sessilis* (L.) R.Br. ex DC. in reducing oxidative stress. Phytomolecules like Vitexin and vitexin analogs (An et al., 2012), Kaempferol and kaempferol analogs (Park et al., 2006), Quercetin and quercetin analogs (Zhang et al., 2011), Acacetin analogs (Li et al., 2019), Isorhamnetin-3-O-robinobioside (Boubaker et al., 2012), Stigmasterol (Liang et al., 2020), Campesterol (Yoshida and Niki, 2003), β-Sitosterol (Gupta et al., 2011), Spinasterol (Adebiyi et al., 2018), Ellagic acid (Priyadarsini et al., 2002), Ferulic acid (Graf, 1992), p-Coumaric acid (Kiliç and Yeşiloğlu, 2013), 4-Hydroxybenzoic acid (Velika and Kron, 2012), 2,5-Dihydroxybenzoic acid (Calderón Guzmán et al., 2007), Chlorogenic acid (Sato et al., 2011), Ionone (Liu et al., 2009), β-Carotene (Paiva and Russell, 1999), Ricinoleic acid (Park et al., 2020), Dopamine-betaxanthin (Cai et al., 2003), and 3-Methoxytyramine-betaxanthin (Cai et al., 2003) which were earlier been reported from *Alternanthera sessilis* (L.) R.Br. ex DC., may be responsible for its antioxidant action.

### Antiparkinsonism/Antidementia Property

Khamphukdee and the team had evaluated the antidementia activity of the ethanolic extract obtained from the whole plant of *Alternanthera philoxeroides* (Mart.) Griseb. They had noticed various mechanisms behind it like inhibition of lipid peroxidation in the whole brain, downregulation of neuroinflammatory cytokines (IL-1β, IL-6, and TNF-α), etc (Khamphukdee et al., 2021). Phytomolecules like Luteolin and luteolin analogs (Delgado et al., 2021), Vitexin (Malar et al., 2020; Zhang et al., 2021), Quercetin (Yao et al., 2010), Torosaflavone E (Khamphukdee et al., 2021), Demethyl torosaflavone D (Khamphukdee et al., 2021), β-Sitosterol (Kim et al., 2008), Stigmasterol (Park S. J. et al., 2012; Pratiwi et al., 2021), Ursolic acid (Habtemariam, 2019), Oleanolic acid and oleanolic acid analogs (Lin et al., 2021), Caffeic acid (Khan et al., 2013; Deshmukh et al., 2016), Quinic acid (Liu et al., 2020), p-Coumaric acid (Kim H.-B. et al., 2017), β-Carboline (Zhao et al., 2013; Li et al., 2018), Malic acid (Tian et al., 2021), Blumenol A (Emir et al., 2019), Phytol (Sathya et al., 2020), and Pheophytin A (Park et al., 2014) which were earlier reported from *Alternanthera philoxeroides* (Mart.) Griseb., may be responsible for this antidementia property.

Ittiyavirah and Hameed had evaluated the antiparkinsonian activity of silver nanoparticles and ethanolic extract obtained from the whole plant of *Alternanthera sessilis* (L.) R.Br. ex DC. They had observed that the silver nanoparticles as well as the ethanolic extract were able to impart neuroprotection with decrease in catalepsy as well as in muscle rigidity, along with locomotion improvement (Ittiyavirah and Hameed, 2015). Phytomolecules like Vitexin and vitexin analogs (Hu et al., 2018), Kaempferol and kaempferol analogs (Filomeni et al., 2012), Quercetin-3-methyl ether (Kim et al., 2009), Quercetin (Lv et al., 2012), Acacetin analogs (Kim S. M. et al., 2017), Stigmasterol (Haque and Moon, 2018), β-Sitosterol (Kim et al., 2008), Spinasterol (Jeong et al., 2010), Ellagic acid (Baluchnejadmojarad et al., 2017), Ferulic acid (Haque et al., 2015), p-Coumaric acid (Vauzour et al., 2010), 4-Hydroxybenzoic acid (Winter et al., 2017), Chlorogenic acid (Singh et al., 2018), and Ionone (Ma et al., 2014) which were previously been reported from *Alternanthera sessilis* (L.) R.Br. ex DC., may be responsible for the antiparkinsonian activity.

### Antiprotozoal Activity

Koolen and the team had isolated compounds like Alternamide A-B and Alternamine A-B from the aerial parts of *Alternanthera littoralis* P.Beauv. and evaluated for their antiprotozoal activity againt protozoal strains viz. *Trypanosoma cruzi trypomastigotes* and *Leishmania amazonensis*. They had observed that out of all the tested compounds, Alternamine A was the most efficient one (Koolen et al., 2017).

### Antispasmodic Activity

Garín-Aguilar and the team had antispasmodic activity of aqueous, hexane, methanolic extract, and fractions of methanol extract (F_1_-F_6_) obtained from the leaves of *Alternanthera sessilis* (L.) R.Br. ex DC. (Garín-Aguilar et al., 2013). while Saqib and Janbaz had used 70% ethanolic extract of the whole plant and its dichloromethane, aqueous fractions (Saqib and Janbaz, 2016). They had observed that *Alternanthera sessilis* (L.) R.Br. ex DC. possesses significant antispasmolytic activity. Phytomolecules like Vitexin and vitexin analogs (Ragone et al., 2007), Quercetin and quercetin analogs (Lozoya et al., 1994; Morales et al., 1994), Acacetin analogs (González-Trujano et al., 2012), Stigmasterol (Ammar et al., 2009), β-Sitosterol (Rehman et al., 2012), and Ellagic acid (Krenn et al., 2011) which were previously been reported from *Alternanthera sessilis* (L.) R.Br. ex DC., may be the contributors towards the antispasmodic activity of the extracts.

### Antiviral Activity

Rattanathongkom and the team had isolated Chikusetsusaponin IVa isolated from the whole plant of *Alternanthera philoxeroides* (Mart.) Griseb. and evaluated antiviral activity against various viral cell lines through *in vitro* and *in vivo* assays. They had observed the dose-dependent activity along with the potential of Chikusetsusaponin IVa in inhibiting the viral protein synthesis (Rattanathongkom et al., 2009).

### Central-Stimulating Activity

Mondal and the team had evaluated the central stimulating potential of the ethanolic extract obtained from the leaves of *Alternanthera sessilis* (L.) R.Br. ex DC. Results were quite significant (Mondal et al., 2014). Phytoconstituents acting on GABA receptors like Ricinoleic acid (Witt et al., 2002), Chlorogenic acid (Hara et al., 2014), p-Coumaric acid (Scheepens et al., 2014), Ferulic acid (Cheng et al., 2010; Sonar et al., 2019), Ellagic acid (Girish et al., 2013), Spinasterol (Socała et al., 2015), Stigmasterol (Karim et al., 2021), Acacetin analogs (Gálvez et al., 2015), Vitexin and vitexin analogs (Zhu et al., 2016; de Oliveira et al., 2020), and Quercetin and quercetin analogs (Goutman and Calvo, 2004; Kim et al., 2014) which were previously been reported from *Alternanthera sessilis* (L.) R.Br. ex DC., may be behind this GABA receptor mediated central-stimulating activity.

### Gastrointestinal Protective Activity

Astudillo-Vázquez and the team had evaluated the gastrointestinal protective potential of the aqueous and ethanolic extracts obtained from the whole plant of *Alternanthera sessilis* (L.) R.Br. ex DC. They noticed that the antidiarrheal property i.e. decreasing the gastrointestinal content is the major factor behind the gastrointestinal protective activity of *Alternanthera sessilis* (L.) R.Br. ex DC. (Astudillo-Vázquez et al., 2008). Phytomolecules like Vitexin and vitexin analogs (Figer et al., 2017), Kaempferol and kaempferol analogs (Beber et al., 2017; Campos-Vidal et al., 2021), Quercetin and quercetin analogs (de la Lastra et al., 1994), Stigmasterol (Sánchez-Mendoza et al., 2008), β-Sitosterol (Sánchez-Mendoza et al., 2008), Ellagic acid (Beserra et al., 2011), Ferulic acid (Shahid et al., 2018), p-Coumaric acid (Panda and Suresh, 2015), Chlorogenic acid (Ahmed et al., 2021), and β-Carotene (Mózsik et al., 1996) which were earlier reported from *Alternanthera sessilis* (L.) R.Br. ex DC., may be responsible for this gastrointestinal protective potential.

### Hepatoprotective Activity

Lin and the team had evaluated the hepatoprotective activity of the aqueous extract obtained from the whole plant of *Alternanthera sessilis* (L.) R.Br. ex DC. (Lin et al., 1994). while Bhuyan and the team had evaluated the hepatoprotective potential of the methanolic extract obtained from the whole plant (Bhuyan et al., 2017). Both these independent researches finally concluded that the *Alternanthera sessilis* (L.) R.Br. ex DC. is hepatoprotective. Phytomolecules like Vitexin and vitexin analogs (Duan et al., 2020), Kaempferol and kaempferol analogs (Wang M. et al., 2015; Wang et al., 2015c), Quercetin-3-methyl ether (Tseng et al., 2012), Quercetin and quercetin analogs (Miltonprabu et al., 2017), Acacetin analogs (Cho H.-I. et al., 2014), Stigmasterol (Carter et al., 2007), β-Sitosterol (Abdou et al., 2019), Ellagic acid (Girish and Pradhan, 2012), Ferulic acid (Rukkumani et al., 2004), p-Coumaric acid (Parvizi et al., 2020), 2,5-Dihydroxybenzoic acid (Pujari and Bandawane, 2021), Chlorogenic acid (Chen et al., 2019), and β-Carotene (Manda and Bhatia, 2003) which were previously reported from *Alternanthera sessilis* (L.) R.Br. ex DC., may be the contributory constituents towards the elicited hepatoprotective activity.

### Immunomodulatory Activity

Several research teams had independently assessed the immunomodulatory potential of *Alternanthera sessilis* (L.) R.Br. ex DC.: Biella and the team had used aqueous extract of the whole plant (Biella et al., 2008); Guerra and the team had used aqueous extract of aerial parts (Guerra et al., 2003); while Moraes and the team had used aqueous and ethanolic extract of leaves as well as tetrahydrofuran, dichloromethane, aqueous, petroleum ether soluble fraction (Moraes et al., 1994). These studies validated the immunomodulatory property of *Alternanthera sessilis* (L.) R.Br. ex DC. Phytomolecules like Vitexin and vitexin analogs (Rosa et al., 2016), Kaempferol and kaempferol analogs (Lin et al., 2011; Swarnalatha et al., 2015), Quercetin-3-methyl ether (Martino et al., 2016), Quercetin and quercetin analogs (Manjunath and Thimmulappa, 2021), Acacetin analogs (Zhao et al., 2014), Stigmasterol (Antwi et al., 2017b), β-Sitosterol (Desai et al., 2009), Ellagic acid (Abuelsaad et al., 2013), Ferulic acid (He F. et al., 2021), p-Coumaric acid (Pragasam et al., 2012), Chlorogenic acid (Guo et al., 2021), and β-Carotene (Jyonouchi et al., 2009) which were previously been reported from *Alternanthera sessilis* (L.) R.Br. ex DC., may be responsible for this immunomodulatory potential.

Moraes and the team had also evaluated the immunomodulatory activity of aqueous and ethanolic extract of leaves as well as tetrahydrofuran, dichloromethane, aqueous, petroleum ether soluble fractions obtained from *Alternanthera brasiliana* (L.) Kuntze and *Alternanthera littoralis* P.Beauv. (Moraes et al., 1994). Phytomolecules like Vitexin and vitexin analogs (Rosa et al., 2016), Kaempferol and kaempferol analogs (Lin et al., 2011; Swarnalatha et al., 2015), Quercetin and quercetin analogs (Manjunath and Thimmulappa, 2021), Tricin (Santos et al., 2017), Stigmasterol (Antwi et al., 2017b), β-Sitosterol (Desai et al., 2009), Ferulic acid (He F. et al., 2021), p-Coumaric acid (Pragasam et al., 2012), and Chlorogenic acid (Guo et al., 2021) which were previously reported from *Alternanthera brasiliana* (L.) Kuntze, may be responsible towards its immunomodulatory activity. Phytomolecules like Vitexin and vitexin analogs (Rosa et al., 2016), Kaempferol (Lin et al., 2011; Swarnalatha et al., 2015), Quercetin-3-methyl ether (Martino et al., 2016), Quercetin and quercetin analogs (Manjunath and Thimmulappa, 2021), Acacetin analogs (Zhao et al., 2014), Stigmasterol (Antwi et al., 2017b), and Hydroxytyrosol (Shan and Miao, 2022) which were previously reported from *Alternanthera littoralis* P.Beauv., may be responsible for its immunomodulatory activity.

### Insecticidal Property

Coutinho and the team had evaluated the insecticidal potential of the ethanolic extract obtained from the leaves of *Alternanthera brasiliana* (L.) Kuntze. against *Drosophila melanogaster* (Harwich strain). They found that the tested concentrations of the ethanolic extract were having a mild insecticidal effect, and that too after 24–48 h exposure (Coutinho et al., 2017). Phytomolecules like Kaempferol and kaempferol analogs (Zhang et al., 2016), Quercetin and quercetin analogs (Mesbah et al., 2007), Stigmasterol (Gade et al., 2017), β-Sitosterol (Zolotar et al., 2002), Spinasterol (Ahmed et al., 2020), and Ferulic acid (Yang et al., 2017) which were previously isolated from *Alternanthera brasiliana* (L.) Kuntze., may be responsible for this insecticidal property.

### Lithotriptic/Antiurolithiatic Activity

Dhanya and the team had evaluated the antiurolithiatic activity of Kalka—fine paste of macerated fresh plant material of *Alternanthera sessilis* (L.) R.Br. ex DC. while Babu and the team had used ethanolic extract of the whole plant for the assessment of antiurolithiatic activity (Dhanya et al., 2017; Babu et al., 2021). Results obtained by both these independent studies are quite significant and reflects the potential of *Alternanthera sessilis* (L.) R.Br. ex DC. as lithotriptic agent. Phytomolecules like Kaempferol and kaempferol analogs (Cechinel-Zanchett et al., 2020), Quercetin and quercetin analogs (Dinnimath et al., 2017), Stigmasterol (Lobine et al., 2020), and Ferulic acid (Zhao et al., 2019) which were previously been reported from *Alternanthera sessilis* (L.) R.Br. ex DC., may be responsible for this antiurolithiatic activity.

### Larvicidal Activity

Babu and the team had also evaluated the larvicidal property of ethanolic extract obtained from the whole plant of *Alternanthera sessilis* (L.) R.Br. ex DC. They found that the ethanolic extract was having a dose dependent percent mortality against mosquito larvae (Babu et al., 2021). Phytomolecules like Stigmasterol (Gade et al., 2017), β-Sitosterol (Angajala and Subashini, 2018), and Ferulic acid (Pavela, 2011), which were earlier isolated from *Alternanthera sessilis* (L.) R.Br. ex DC., may be responsible behind this larvicidal activity.

### Nootropic Activity

Gupta and Singh had evaluated the nootropic activity of methanolic extract obtained from the leaves of *Alternanthera sessilis* (L.) R.Br. ex DC. And results were quite promising (Gupta and Singh, 2012b). Phytomolecules like Kaempferol and kaempferol analogs (Das et al., 2018), Quercetin and quercetin analogs (Halder et al., 2015), Ellagic acid (Bansal et al., 2017; Kiasalari et al., 2017), and Ferulic acid (Yang et al., 2016; Mhillaj et al., 2017) which had been previously isolated from *Alternanthera sessilis* (L.) R.Br. ex DC., may be the contributing phytomolecules towards this nootropic activity.

### Photoprotective Activity

Alencar Filho and the team had evaluated the photoprotective effect of the gel prepared from 5% w/w of extract *Alternanthera brasiliana* (L.) Kuntze enriched in flavonoids. They had observed that the stabilization of the ROS and resonating permission are the mechanisms behind this photoprotective activity of the gel extract (Alencar Filho et al., 2020). Phytomolecules like Kaempferol and kaempferol analogs (Monici et al., 1994), Quercetin and quercetin analogs (Saija, 2003; Gonçalves et al., 2019), Tricin (Moon et al., 2018), Stigmasterol (Bayer et al., 2011), β-Sitosterol (Bayer et al., 2011), Ferulic acid (Lin et al., 2005; Peres et al., 2018), p-Coumaric acid (Biswas et al., 2021), and Chlorogenic acid (Wang et al., 2021) which were earlier reported from *Alternanthera brasiliana* (L.) Kuntze, may be responsible for this photoprotective property of the gel extract.

### Sedative Property

Oyemitan and the team had evaluated the sedative action of the ethanolic extract obtained from the leaves of *Alternanthera brasiliana* (L.) Kuntze. They had observed that the ethanolic extract was expressing the sedative property by acting on stimulatory or central excitatory channels (Oyemitan et al., 2015). Phytomolecules like Quercetin and quercetin analogs (Nakhaee et al., 2021), β-Sitosterol (Aguirre-Hernández et al., 2007), and Ferulic acid (Tu et al., 2012) which were previously been reported from *Alternanthera brasiliana* (L.) Kuntze., may be responsible for this sedative action.

### Wound Healing Property

Barua and the team had reported several studies validating the wound healing property of *Alternanthera brasiliana* (L.) Kuntze (Barua et al., 2009; Barua C. et al., 2012; Baru et al., 2012; Barua C. C. et al., 2012). Phytomolecules like Vitexin and vitexin analogs (Bektas et al., 2020), Kaempferol and kaempferol analogs (Petpiroon et al., 2015; Özay et al., 2019), Quercetin and quercetin analogs (Gomathi et al., 2003), Tricin (Han et al., 2016), β-Sitosterol (Abbas et al., 2019), Ferulic acid (Ghaisas et al., 2014), p-Coumaric acid (Kong et al., 2013; Boeing et al., 2020), and Chlorogenic acid (Bagdas et al., 2015) which had been isolated from *Alternanthera brasiliana* (L.) Kuntze previously, may be responsible for this wound healing property.

Muniandy and the team had evaluated the wound healing action of the 90% hydroethanolic extract obtained from the stem of *Alternanthera sessilis* (L.) R.Br. ex DC. while Jalalpure and the team had used chloroform extract obtained from the leaves *Alternanthera sessilis* (L.) R.Br. ex DC. Both these teams had independently ascertained the wound healing property of *Alternanthera sessilis* (L.) R.Br. ex DC. (Jalalpure et al., 2008; Muniandy et al., 2018b). Phytomolecules like Vitexin and vitexin analogs (Bektas et al., 2020), Kaempferol and kaempferol analogs (Petpiroon et al., 2015; Özay et al., 2019), Quercetin and quercetin analogs (Gomathi et al., 2003), Acacetin analogs (Bhat et al., 2013), β-Sitosterol (Abbas et al., 2019), Ellagic acid (Mo et al., 2014), Ferulic acid (Ghaisas et al., 2014), p-Coumaric acid (Kong et al., 2013; Boeing et al., 2020), and Chlorogenic acid (Bagdas et al., 2015), β-Carotene (Gerber and Erdman, 1982), and Ricinoleic acid (Nada et al., 2018) which had earlier reported from *Alternanthera sessilis* (L.) R.Br. ex DC., may be responsible for this wound healing property.

**FIGURE 4:**
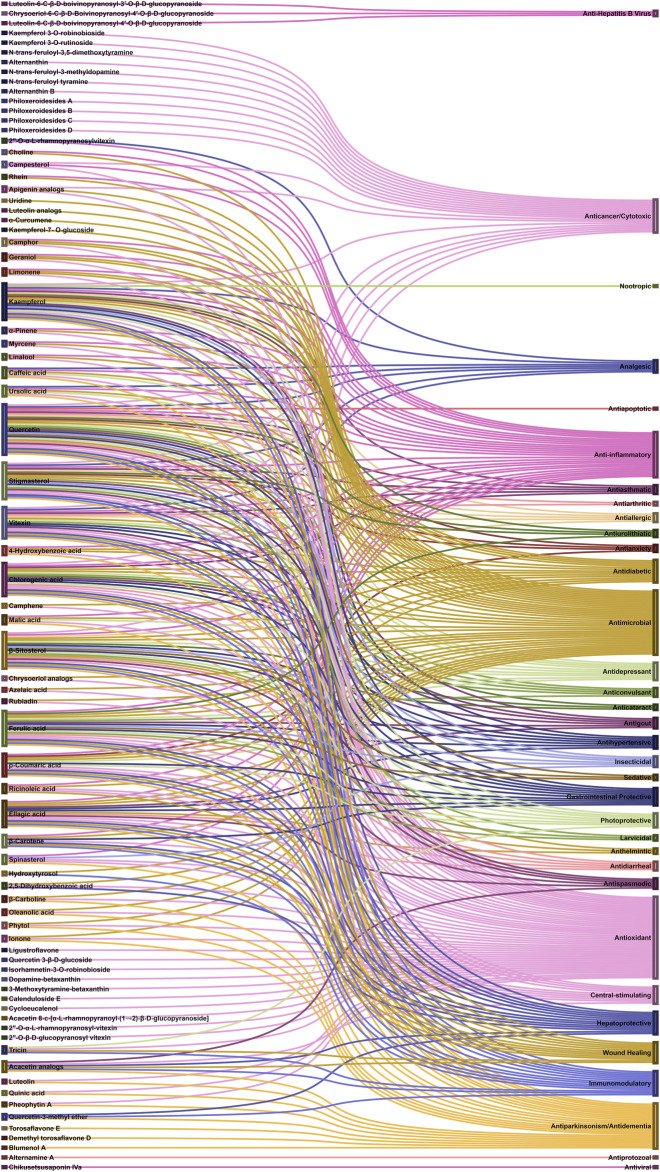
Bioactive Molecules and their elicited pharmacological activities. This information was collected as a cross-sectional literature review while exploring the possible bioactive molecules behind the pharmacological activities of the crude extracts obtained from various species of Alternanthera Genus.

After this exhaustive cross-literature review for the bioactive compounds that may be responsible elements behind the potent pharmacological actions elicited by the extracts, we have summarized those in a smart interactive illustration (Figure 4).

It is indispensable to confirm if traditional claims of *Alternanthera* species have been proven by systematic scientifically designed pharmacological (preclinical or clinical) studies. Traditional claims and reported pharmacological activities of various species are presented in Table 3, and observations are as follows:

a) Traditional claims of some species (*Alternanthera brasiliana* (L.) Kuntze, *Alternanthera caracasana* Kunth, *A. dentata* (now reclaimed as *Alternanthera brasiliana* (L.) Kuntze), *A. ficoides* (now reclaimed as *Alternanthera sessilis* (L.) R.Br. ex DC.), *Alternanthera littoralis* P.Beauv., *A. maritima* (now reclaimed as *Alternanthera littoralis* P.Beauv.), *Alternanthera nodiflora* R.Br*.*, *Alternanthera paronychioides* A.St.-Hil., *Alternanthera porrigens* (Jacq.) Kuntze, *Alternanthera pungens* Kunth, *Alternanthera sessilis* (L.) R.Br. ex DC., *A. tenella* (now reclaimed as *Alternanthera sessilis* (L.) R.Br. ex DC.)*,* and *A. triandra* (now reclaimed as *Alternanthera sessilis* (L.) R.Br. ex DC.)) have not been validated scientifically.
b) Traditionally used species like *Alternanthera caracasana* Kunth and *Alternanthera porrigens* (Jacq.) Kuntze have not been investigated for any pharmacological activities. These species hold great potential for future research intending to validate traditional claims.
c) Species (*Alternanthera brasiliana* (L.) Kuntze, *Alternanthera paronychioides* A.St.-Hil., *Alternanthera philoxeroides* (Mart.) Griseb., and *Alternanthera sessilis* (L.) R.Br. ex DC.) have been screened for those pharmacological actions which are not claimed traditionally. These species may have been chosen following a chemotaxonomical or ecological approach.
d) Literature did not reveal any traditional use of three species (*Alternanthera bettzickiana* (Regel) G.Nicholson, *Alternanthera hirtula* (Mart.) R.E.Fr., and *Alternanthera praelonga* A.St.-Hil.) but evaluated for varied pharmacological activities.

**TABLE 3:** Relationship between reported scientific pharmacological activities of *Alternanthera* species and their traditional claims.

Sr No	Species name	Traditional uses	Scientifically validated traditional claims	Traditional claims not validated scientifically	Other pharmacological activities
1	*Alternanthera bettzickiana* (Regel) G.Nicholson	—	—	—	Antibacterial, anticancer, antimicrobial, antioxidant
2	*Alternanthera brasiliana* (L.) Kuntze	In the treatment of headaches, cough, colds, grippe, fever, and diarrhea	Analgesic, antioxidant	Antidiarrhoeal, antipyretic	Allelopathic, antianxiety, antibacterial, anticancer, anticonvulsant, antifungal, anti-inflammatory, insecticide, sedative, and wound healing
3	*Alternanthera caracasana* Kunth	In the treatment of dysentery, diarrhea, and fever	—	Anti-dysentery, antidiarrhoeal, and antipyretic	—
4	*Alternanthera dentata* (Now reclaimed as *Alternanthera brasiliana* (L.) Kuntze)	In the treatment of inflammation, pain	—	Analgesic, anti-inflammatory	Antimicrobial, antioxidant
5	*Alternanthera ficoidea* (L.) P.Beauv	In the treatment of heart and cancer problems	Antioxidant	Anticancer, cardiotonic	—
6	*Alternanthera hirtula* (Mart.) R.E.Fr	—	—	—	Anticancer, antioxidant
7	*Alternanthera littoralis* P.Beauv	In the treatment of infectious and inflammatory diseases	Antioxidant	Anti-inflammatory	—
8	*Alternanthera maritima* (now reclaimed as *Alternanthera littoralis* P.Beauv.)	In the treatment of inflammation, viral infections, cancer, malaria, and diarrhea	Anti-inflammatory, antimicrobial	Antiviral, antidiarrhoeal, and anticancer	—
9	*Alternanthera nodiflora* R.Br	In the treatment of skin problems, degenerative and microbial infections	Antimicrobial	Skin protection	—
10	*Alternanthera paronychioides* A.St.-Hil	In the treatment of hyperuricemia, rheumatic arthritis, nephritis, gout, cystitis, diabetes, and systemic neuralgia	Antioxidant	Antihyperuricemia, antiarthritic, antigout, renal protective, antidiabetic, anti-inflammatory, and analgesic	Antiapoptotic
11	*Alternanthera philoxeroides* (Mart.) Griseb	In the treatment of influenza	Antioxidant, antiviral	—	α-glucosidase, inhibitory, analgesic, antianxiety, antiarthritic, anticancer, antidepressant, antidiabetic, anti-HBV, anti-inflammatory, antimicrobial
12	*Alternanthera porrigens* (Jacq.) Kuntze	In the treatment of hepatitis, kidney problems, influenza	—	Hepatoprotective, analgesic, antiviral, renal protective	—
13	*Alternanthera praelonga* A.St.-Hil	—	—	—	Anticancer, antioxidant
14	*Alternanthera pungens* Kunth	In the treatment of nasopharyngeal infections, pain, gonorrhea, menstrual disorder, dysentery, cholera, and many parasitic diseases	Anti-inflammatory, antimicrobial, antioxidant	Analgesic, anti- dysentery	—
15	*Alternanthera repens* (now reclaimed as *Alternanthera sessilis* (L.) R.Br. ex DC.)	—	—	—	Antibacterial, antidiarrhoeal, antispasmodic, gastrointestinal protective
16	*Alternanthera sessilis* (L.) R.Br. ex DC.)	In the treatment of stomach pain, ulcer, and gastric problems	Analgesic, antioxidant	Antiulcer, gastroprotective	α-glucosidase inhibitory, anthelmintic, anti-allergic, antiarthritic, antiasthmatic, antibacterial, anticancer, anticataract, antidepressant antidiabetic, antifungal, antihypertensive, anti-inflammatory, antimicrobial, anti-parkinsonism, hepatoprotective, nootropic, and wound healing
17	*Alternanthera tenella* (Now reclaimed as *Alternanthera sessilis* (L.) R.Br. ex DC.))	In the treatment of urinary problems, fever, menstruation problem, inflammations, and ovarian diseases	Anti-inflammatory, antimicrobial, antioxidant	Renal protective, antipyretic	Immunomodulatory, inhibition of lymphocyte activation, and anticancer
18	*Alternanthera triandra* (Now reclaimed as *Alternanthera sessilis* (L.) R.Br. ex DC.))	In the treatment of fever, lactation problem	—	Antipyretic	—

### Toxicological Studies

Hydroalcoholic extract of *Alternanthera brasiliana* (L.) Kuntze and *Alternanthera bettzickiana* (Regel) G.Nicholson leaves was orally administered (200 mg/kg dose) for 14 days in mice to observe any change in behavior of animals (Kasthuri and Ramesh, 2018). Further, hematological and histopathological changes were also observed. Sub-acute toxicity study suggested that both extracts samples did not show any harmful side effects. Hydroethanolic leaf extract of *Alternanthera bettzickiana* (Regel) G.Nicholson displayed a progressively powerful cytotoxic impact on DLA cell lines than *Alternanthera brasiliana* (L.) Kuntze extract.

The oral acute toxicity study was conducted on 95% ethanolic extract of *Alternanthera philoxeroides* (Mart.) Griseb. at the dose of 500 mg/kg in male and female rodents (Thanabhorn et al., 2005). The ethanolic extract did not show mortality and gross morphological alterations in the organs of rodents. Oral administration of 1,000 mg/kg/day for 14 days showed no significant changes in the body and inner organs weights, hematological and clinical parameters.

### Clinical Studies

The studies have shown antiretroviral activity of *Alternanthera pungens* Kunth herbal tea due to antioxidant potential when administered to HIV patients (Djohan et al., 2009). Blood samples were taken from fasted patients who received an *Alternanthera pungens* Kunth tea for 12 months every day before dinner. The markers of oxidative stress (malondialdehyde and advanced oxidation protein end products), plasma T lymphocytes, transaminases, and creatinine were determined in the blood sample. A significant decrease in concentrations of markers of oxidative stress and an increase in plasma levels of CD4 and CD8 T cells after this period were observed. Further, no signs of hepatic and renal toxicity were seen in HIV patients.

In another case study, the potential of *Alternanthera sessilis* (L.) R.Br. ex DC., *Momordica charantia* L.*,* and *Colocasia esculenta* (L.) Schott were investigated in reducing postprandial blood glucose levels in healthy human subjects and patients with type II diabetes (Bachok et al., 2014). The results of the clinical report suggested that *Alternanthera sessilis* (L.) R.Br. ex DC. reduced the non-significant glucose level in 3 h in comparison to standard control diet in healthy and diseased subjects. This case study was conducted in India with eight healthy subjects and six diabetic subjects.

## Conclusion

Scrutiny of available literature reveals that out of 139 species of the genus *Alternanthera:*

a) Nine species have been investigated phytochemically,
b) Fifteen species possess strong ethnopharmacological records,
c) Twelve species have been scientifically evaluated in the *in vitro* or *in vivo* experimental models for various pharmacological activities,
d) Three species have been subjected to toxicity studies for establishing safety profiles,
e) Two species have been examined for clinical studies.

To date, 129 compounds have been isolated from 9 species of *Alternanthera.* 129 bioactive compounds were classified in 11 phytochemical classes, covering information about 40 flavonoids, 17 triterpenoid/saponins, 15 sterols, 12 alkaloids, 10 phenolic compounds, 3 ionone, 1 benzopyran, 3 hydroxycinnamic acids, 4 anthraquinone, 8 volatile oils and 17 miscellaneous compounds. Flavonoids (∼32%) constitute the main class of phytoconstituents in the genus *Alternanthera* followed by triterpenoids (∼13%). The isolated triterpenoids such as oleanolic acid, ursolic acid, and flavonoids such as luteolin, apigenin, vitexin, kaempferol, quercetin aglycones and their glycosides from the genus have proven therapeutic value. In terms of the phytochemical exploration, the most explored species of Alternanthera genus were *Alternanthera philoxeroides* (Mart.) Griseb. (**52** compounds), *Alternanthera sessilis* (L.). R.Br. ex DC. (**45** compounds), *Alternanthera brasiliana* (L.). Kuntze (**32** compounds), and *Alternanthera littoralis* P.Beauv (**24** compounds). *Alternanthera sessilis* (L.) R.Br. ex DC. has so far yielded a diverse class of compounds, like benzopyran, flavonoids, sterols, triterpenoid/saponin, phenolic compounds, ionone, and miscellaneous compounds. Similarly, *Alternanthera philoxeroides* (Mart.) Griseb. has also yielded a diverse class of compounds like flavonoids, sterols, triterpenoid/saponins, phenolic compounds, anthraquinone, alkaloids, and miscellaneous compounds.While volatile oil related compounds were extracted only from *Alternanthera pungens* Kunth, ionone analogues were isolated from *Alternanthera sessilis* (L.) R.Br. ex DC. only and hydroxycinnamic acids were reported only from *Alternanthera bettzickiana* (Regel) G.Nicholson. Researchers could explore rest of the species of Alternanthera genus to check if containing ionone analogues, volatile oils, and hydroxycinnamic acids. Further, the species of Alternanthera genus which were least explored in terms of phytochemical characterization is also leading for possible opportunities for the researchers.

To the best of our knowledge, the phytochemial characterization of *Alternanthera paronychioides* A.St.-Hil., *Alternanthera caracasana* Kunth, *Alternanthera nodiflora* R.Br., and *Alternanthera ficoidea* (L.) P.Beauv. was not yet done, leaving an ample scope for the researchers.

Some phytoconstituents like quercetin, vitexin, chlorogenic acid, kaempferol, ferulic acid, β-sitosterol, p-coumaric acid, caffeic acid, quinic acid, etc had been reported from more than one species of Alternanthera. Probably, we could say that these phytoconstituents may be common secondary metabolites in Alternanthera genus. So, we recommend the researchers to explore the rest of the Alternanthera species for these common metabolites. These metabolites could serve as biomarkers for them.

As twelve species of *Alternanthera* have been investigated scientifically for pharmacological activities, only 9 species of the genus have been explored phytochemically. Few medicinally promising *Alternanthera* species have not been taken into consideration for phytochemical studies. The existing literature demonstrates that 5 species of genus *Alternanthera* such as.

*Alternanthera brasiliana* (L.) Kuntze, *Alternanthera caracasana* Kunth, *Alternanthera ficoidea* (L.) P.Beauv., *Alternanthera nodiflora* R.Br.*,* and *Alternanthera paronychioides* A.St.-Hil. have been scientifically reported to exhibit various pharmacological activities, but these species have never been subjected to bioactivity directed fractionation to isolate bioactive phytoconstituents using appropriate chromatographic techniques. Therefore, natural product scientists should expand their research activities on *Alternanthera* species to isolate more bioactive compounds which can be developed as safer and efficacious lead molecules or potent analogs of bioactive markers. Further, it seems necessary to mention a major research gap in phytochemical studies that no emphasis has been given to standardizing these plants based on marker compounds. Appropriate analytical methods need to be developed using HPLC, HPTLC, or LC-MS for the standardization of *Alternanthera* species. Molecular docking and QSAR studies on selective bioactive markers of these species are also lacking. It has been observed that crude uncharacterized extracts of *Alternanthera* species have been used in most pharmacological studies. This observation attracts attention towards the isolation of bioactive compounds from *Alternanthera* following the bioactivity-guided fractionation approach. Highlighting a mechanistic approach for pharmacological activities is another area of research to be covered. Alternamide A-B and Alternamine A-B were evaluated only for antiprotozoal activity while Chikusetsusaponin IVa was checked for antiviral activity only, leaving a wide scope for the researchers.

Amongst 139 species of *Alternanthera,* only 12 species have shown medicinal value in preclinical studies, and out of these only *Alternanthera pungens* Kunth and *Alternanthera sessilis* (L.) R.Br. ex DC. have been investigated clinically for antiretroviral and antidiabetic activities, respectively. The toxicity studies have been conducted on 3 species such as *Alternanthera bettzickiana* (Regel) G.Nicholson, *Alternanthera brasiliana* (L.) Kuntze*,* and *Alternanthera philoxeroides* (Mart.) Griseb. to establish their safety profile. Please be noted that as per the latest guidelines and recommendations of the ethnopharmacology team, the scientific names of the plants have been reassessed and considered the name given on https://mpns.science.kew.org/mpns-portal/. So the universally recognized name has been mentioned rather than the synonym indicated in the cited articles.

It is finally concluded that a well-planned roadmap of research activities is needed to be designed on traditionally used and medicinally promising plants of genus *Alternanthera,* so that their products and preparations may emerge out to be clinically potential and safe medicines in the treatment of various ailments.
